# Global development assistance for early childhood care and education in 134 low- and middle-income countries, 2007–2021

**DOI:** 10.1136/bmjgh-2024-015991

**Published:** 2024-11-20

**Authors:** Yiqun Luan, Dominic Hodgkin, Jere Behrman, Alan Stein, Linda Richter, Jorge Cuartas, Chunling Lu

**Affiliations:** 1 Heller School for Social Policy and Management, Brandeis University, Waltham, Massachusetts, USA; 2 Schneider Institutes for Health Policy and Research, Heller School for Social Policy and Management, Brandeis University, Waltham, Massachusetts, USA; 3 Department of Economics, University of Pennsylvania, Philadelphia, Pennsylvania, USA; 4 Blavatnik School of Government, University of Oxford, Oxford, UK; 5 MRC/Wits Rural Public Health and Health Transitions Research Unit (Agincourt), Faculty of Health Sciences, School of Public Health, University of the Witwatersrand, Johannesburg, South Africa; 6 Africa Health Research Institute, Durban, KwaZulu-Natal, South Africa; 7 DSI-NRF Centre of Excellence in Human Development, University of the Witwatersrand, Johannesburg, South Africa; 8 Stellenbosch Institute for Advanced Study (STIAS), Stellenbosch, South Africa; 9 Department of Applied Psychology, New York University, New York, New York, USA; 10 Centro de Estudios sobre Seguridad y Drogas (CESED), Universidad de los Andes, Bogotá, Colombia; 11 Division of Global Health Equity, Brigham and Women's Hospital, Boston, Massachusetts, USA; 12 Department of Global Health and Social Medicine, Harvard Medical School, Boston, Massachusetts, USA

**Keywords:** child health, paediatrics

## Abstract

**Introduction:**

Low- and middle-income countries (LMICs) often dedicate limited domestic funds to expand quality early childhood care and education (ECCE), making complementary international donor support potentially important. However, research on the allocation of international development assistance for ECCE has been limited.

**Methods:**

We analysed data from the Creditor Reporting System on aid projects to assess global development assistance for ECCE in 134 LMICs from 2007 to 2021. By employing keyword-searching and funding-allocation methods, we derived two estimates of ECCE aid: a lower-bound estimate comprising projects primarily focusing on ECCE and an upper-bound estimate comprising projects with both primary and partial ECCE focus, as well as those that could benefit ECCE but did not include ECCE keywords. We also assessed aid directed to conflict-affected countries and to ECCE projects integrating COVID-19-related activities.

**Results:**

Between 2007 and 2021, the lower-bound ECCE aid totaled US$3646 million, comprising 1.7% of the total US$213 279 million allocated to education. The World Bank led in ECCE aid, contributing US$1944 million (53.3% out of total ECCE aid). Low-income countries received less ECCE aid per child before 2016, then started to catch up but experienced a decrease from US$0.8 (2020) per child to US$0.6 (2021) per child. Funding for ECCE projects with COVID-19 activities decreased from a total of US$50 million in 2020 to US$37 million in 2021, representing 11.4% and 6.6% of annual total ECCE aid, respectively. Over 15 years, conflict-affected countries received an average of US$0.3 per child, a quarter of the aid received by non-conflict-affected countries (US$1.2 per child).

**Conclusion:**

Although ECCE aid increased significantly between 2007 and 2021, its proportion of total educational aid fell short of UNICEF’s suggested 10% minimum. Recommendations include increasing the share of ECCE aid in total educational aid, increasing aid to low-income and conflict-affected countries, and investing more in preparing ECCE programmes for future global crises.

**WHAT IS ALREADY KNOWN ON THIS TOPIC**

- Quality early childhood care and education (ECCE) is widely recognised as fundamental for fostering lifelong individual and societal benefits.
- The United Nations target universal access to ECCE by 2030 in its Sustainable Development Goals.
- The scaling up of ECCE in low- and middle-income countries (LMICs) faces challenges stemming from insufficient allocation of domestic financial resources to ECCE, underscoring the value of international donors to narrowing this financial gap.

**WHAT THIS STUDY ADDS**

- Between 2007 and 2021, ECCE aid in 134 LMICs totaled US$3646 million, representing 1.7% of the total educational aid.
- Before 2016, low-income countries received less annual international ECCE aid per child compared with middle-income countries, but the gap began to narrow thereafter; however, between 2020 and 2021, low-income countries experienced a decline in funding, while middle-income countries continued to see an increase.
- On average, ECCE aid per child allocated to conflict-affected countries amounted to only a quarter of that received by non-conflict-affected countries.
- Additionally, funding for ECCE projects with COVID-19-related activities declined both in amount and as a percentage of total ECCE aid between 2020 and 2021.

**HOW THIS STUDY MIGHT AFFECT RESEARCH,PRACTICE OR POLICY**

- Meeting the Sustainable Development Goal of universal access to ECCE would be facilitated by greater allocation of development assistance and more targeted support for children living in low-income nations and those affected by global crises.

## Introduction

 Extensive interdisciplinary research underscores the significant connection between children’s engagement in quality early childhood care and education (ECCE) and a wide array of lifelong individual benefits, as well as broader socioeconomic development.^1^,^5^ Access to early learning opportunities, including ECCE programmes, is one of the five key components of the Nurturing Care Framework for early childhood development (health, nutrition, security and safety, early learning and responsive caregiving).^6^ Quality ECCE programmes have the potential to mitigate early adversities, foster young children’s cognitive, language and socioemotional skills, and prepare them for lifelong learning.^7^

Recognising the crucial role of ECCE, the United Nations set universal access to quality ECCE as part of its educational agenda in the Sustainable Development Goals (SDGs).^8^ Equally important to the other four components of Nurturing Care, access to early learning opportunities, including ECCE, is also recognised as a fundamental right for young children as highlighted in the first joint global report (2024) by UNESCO and UNICEF.^9^ However, despite the global gross enrolment rate for preprimary education rising significantly from 46% in 2010 to 61% in 2020, the average rate in low-income countries (LICs) remains as low as 20%.^10^ Additionally, a series of global crises—including the COVID-19 pandemic, regional armed conflicts and the growing climate crisis—have further exacerbated low ECCE enrolment rates, resulting in unprecedented potential developmental loss for both individuals and societies.^11^,^13^

To promote children’s development amid these global threats, urgent actions are needed to expand and enhance the five components of Nurturing Care, including early learning opportunities such as access to high-quality ECCE programmes. However, in low- and middle-income countries (LMICs), these efforts have long been hindered by limited financial resources for education and a skewed allocation of educational funds towards higher educational levels.^14 15^ Despite evidence showing that every dollar invested in ECCE for the most disadvantaged children can yield a return as high as US$17,^15^ sub-Saharan African countries still allocate, on average, only 0.3% of their public educational budgets on preprimary education.^14^ While domestic governments and households contribute >80% of total educational spending in many LMICs, international donors remain crucial in bridging financial gaps to increase access, improve quality and catalyse reforms.^14 16^ To encourage support for ECCE, UNICEF recommends that at least 10% of educational aid in LMICs be directed to the ECCE sector.^17^

Tracking investments in the five components of Nurturing Care and their socioeconomic facilitators is important for policymakers in LMICs and international donors to understand the resources needed to attain important goals. Previous studies have made extensive efforts in tracking aid for child and maternal care,^18^,^21^ but studies quantifying ECCE aid in LMICs remain limited. Using data from the Organisation for Economic Cooperation and Development (OECD), one study^22^ demonstrated a relatively stable level in aid between 2005 and 2014, in contrast to a substantial increase in funding for primary and secondary education. Another study^15^ estimated that annual aid for ECCE between 2012 and 2015 amounted to a mere US$74 million, constituting only 0.6% of total educational aid. Yet another study^23^ observed fluctuations in ECCE aid between 2007 and 2013, followed by a consistent increase from 2014 to 2016. While valuable, these studies were limited by the short duration of the periods covered, a constrained analysis of aid levels and trends and identification strategies that may have overlooked ECCE aid disbursements in the OECD database.

Our study seeks to enhance understanding of ECCE aid in 134 LMICs between 2007 and 2021. We systematically assessed bilateral and multilateral aid flows from national donors, multilateral institutions and private sources. We examined levels and trends of ECCE aid in terms of the total amount and per age-eligible child for ECCE (hereafter referred to as ‘ECCE-age’ children) at global, regional and country levels. We analysed the pattern of ECCE aid by donors, recipients, aid sectors, implementing agencies and flow types. We also investigated ECCE aid to projects that included COVID-19 prevention and mitigation activities and ECCE aid to conflict-affected countries. Our study aims to establish a baseline for ECCE aid in LMICs, identify active donors and underinvested recipients, examine aid mechanisms and assess the alignment of ECCE aid with global challenges.

## Methods

### Data sources

We extracted global development-assistance data from OECD’s Creditor Reporting System (CRS),^24^ where development assistance refers to aid intended to support the socioeconomic development and welfare of LMICs.^25^ The CRS database is a publicly accessible source that records information on aid projects. These projects are mandatorily reported by all OECD Development Assistance Committee (DAC) member countries and voluntarily reported by non-DAC states, multilateral institutions and private entities.^26 27^ We downloaded the 2007–2020 CRS data in May 2022 and the 2021 CRS data in January 2023. The CRS data included information on all aid projects implemented in 155 recipient countries or territories. We excluded 21 recipients classified as high-income or without income classifications according to the World Bank’s 2020 standard.^28^ This resulted in 134 LMIC recipients being included in our study (online supplemental table S1).

Donors disbursed aid to the 134 LMICs across various years. Online supplemental table S2a–d presents a breakdown of donors and the corresponding years in which each donor reported aid during our study period. We estimated ECCE aid using each year’s available aid data from all reporting donors in that year. Following previous studies and OECD’s recommendations, we excluded CRS data before 2007 because of the high missing data rate; for 2007 onwards, the completeness of the data reached nearly 100%.^2629^,^31^ To assess ECCE aid per ECCE-age child, we calculated the total number of ECCE-age children for each country using data from UNESCO Institute for Statistics.^32^ This calculation involved aggregating each country’s eligible population for early childhood educational development programmes (typically children aged 0–2 years) and preprimary educational programmes (typically children aged 3 years to the primary school entry age).^33^ For cases with missing data in these eligible populations, we applied imputations as detailed in online supplemental text S1.

### Defining and estimating aid for early childhood care and education

#### Defining aid for early childhood care and education

We adhered to UNESCO’s definition of ECCE, which encompasses *programmes designed with a holistic approach to support children’s early cognitive, physical, social and emotional development, characterised by a learning environment and an educational component*.^33^ Furthermore, quality ECCE programmes may also incorporate health (eg, immunisation) and nutrition (eg, free meal) components, serving as platforms to promote early childhood development.^34^ ECCE programmes could be conducted in either formal or non-formal organised settings. Examples of ECCE programmes include daycare, preprimary schools and community-based and centre-based childcare and educational programmes designed for young children in group settings.^33 35^ We defined ECCE aid as development assistance that supports ECCE programmes in LMICs.

#### Estimating aid for early childhood care and education

The CRS database categorises each aid project into a sector first and subsequently into a purpose to specify the intended socioeconomic area of support for the recipients. For ECCE aid, the CRS database creates a ‘basic education’ sector and an ‘early childhood education’ purpose (purpose code: 11240) to allow donors to report aid projects focused on formal and non-formal preschool education.^27^ However, our random check of project descriptions found that some projects with this purpose may exclusively focus on education subsequent to ECCE. Therefore, we conducted a manual review of each project under the 11 240 purpose and excluded projects exclusively focused on educational levels beyond ECCE (171 out of 6941 projects).

We also found that projects reported in other CRS sectors and purposes, such as the ‘other social infrastructure and services’ sector, sometimes involve ECCE activities. This finding emphasises the need for a multisectoral approach when searching for ECCE aid projects. Consequently, we conducted a comprehensive search across all CRS sectors and purposes, excluding the 11 240 purpose. Online supplemental table S3 lists the CRS sectors we used for searching ECCE projects.

To identify projects with ECCE activities from other CRS sectors and purposes, we adopted the methodologies used in previous studies on estimating development assistance on health.^18^,^2029^ We began by creating a list of keywords related to ECCE based on a review of existing ECCE literature. These keywords were then used to search in the titles, short descriptions and long descriptions of aid projects. We categorised the keywords into two groups: (1) ECCE-specific keywords, such as ‘preschool’, which explicitly refer to ECCE programmes and (2) general care/education keywords, such as ‘safe learn’, which refer to care and educational activities without specifying the age of the beneficiaries or educational levels. To improve the search results from the general care/education keywords, we compiled a list of age-related terms, such as ‘under five’ and ‘child/boy/girl’. We then searched for the presence of these terms among the projects that were exclusively identified by the general care/education keywords. Only projects that contained either the ECCE-specific keywords or the combination of the general care/education keywords and the age-related terms were retained for further manual review. We translated and searched the keywords and terms in nine languages. Online supplemental table S4a,b presents the keywords and terms used in this study.

Next, we manually reviewed the projects that fulfilled our search strategy’s criteria. We made decisions on whether a project either (1) primarily focused on ECCE, (2) partially focused on ECCE, (3) focused on childcare or child education without specifying children’s age or educational level. We excluded false positive projects that did not have ECCE or children’s care and educational activities. Online supplemental figure S1 illustrates each step of our project search and review process.

Previous studies have noted that using keyword searches in the CRS database may lead to the omission of certain relevant projects due to the imperfect sensitivity of this strategy.^18 19 36^ To assess our search sensitivity, we randomly selected 10% of aid projects from 2020 within four CRS educational sectors—‘basic education’, ‘secondary education’, ‘postsecondary education’ and ‘education, level unspecified’. We separately applied manual review and keyword search to these selected projects and compared results. We found that our keyword search missed approximately 2.6% of projects manually identified as having ECCE activities, accounting for only 0.04% of the corresponding total funds. This exercise confirmed the robustness of our search strategy.

Following the review, we generated two estimates of ECCE aid: a lower-bound and an upper-bound estimate. The lower-bound estimate included total aid disbursements to projects categorised under the CRS ‘early childhood education’ purpose (except for those 171 projects exclusively focused on educational activities beyond ECCE) and projects deemed as primarily focused on ECCE. The upper-bound estimate included the lower-bound estimate, allocated disbursements from projects partially focused on ECCE and allocated disbursements from projects focused on childcare and child education without specifying children’s age and educational levels. Furthermore, we considered that aid projects under the CRS ‘education, level unspecified’ sector and the ‘general budget support’ sector may also benefit ECCE and allocated these disbursements to the upper-bound estimate.

The allocation was conducted in the following ways. For projects that include ECCE along with educational activities beyond ECCE (eg, primary education), we allocated projects’ disbursements to the country’s ECCE population based on the country’s year-specific proportion of ECCE-age children relative to its total eligible population for the educational levels specified in the project. Given that countries generally have higher (often much higher) expenditures per child for educational activities beyond ECCE than for ECCE-age activities, this allocation almost surely overestimates the allocation to ECCE-age activities. For childcare and child educational projects without children’s age and educational-level specification, we allocated disbursements based on the country’s year-specific proportion of its ECCE-age children to its total eligible population from ECCE to tertiary education. Again, this allocation almost surely overestimates the allocation to ECCE-age activities. We did not allocate disbursements of projects involving ECCE and non-formal educational sector activities, such as projects primarily focused on women empowerment that integrate ECCE activity as a component, due to the lack of detailed information at the activity level. We included the total aid disbursements of these projects in our upper-bound estimate. Lastly, donors may report project recipients as ‘regional’ or ‘bilateral’ without specifying which country is eligible to receive the funds. Based on previous studies,^19 30^ we allocated ‘regional’ funds among LMICs within the region according to their year-specific shares of ECCE-age children and allocated ‘bilateral’ funds to every country in proportion to its year-specific share of the total ECCE-age children.

Online supplemental table S5 presents details of all the allocation strategies we used in constructing the upper-bound estimate. Our procedure aligned with previous studies^2029^,^31 37^ by using donors’ gross disbursements rather than commitments to quantify ECCE aid. Out of the 31 268 gross disbursements from projects primarily and partially focused on ECCE, we excluded 132 disbursements with negative values, as they are inconsistent with OECD’s definition of gross disbursement, which employs positive values to reflect gross aid flows from donors to recipients.^38^ Additionally, we excluded 1476 disbursements with missing monetary values.

### Defining and estimating ECCE aid to projects with COVID-19 activities

We paid special attention to aid for ECCE programmes with COVID-19 activities. The CRS database used a purpose ‘COVID-19 control’ to track projects with activities related to COVID-19 immunisation, testing, prevention, treatment and postrecovery therapies and a hashtag variable ‘#COVID-19/COVID-19’ to track projects aimed at mitigating socioeconomic impacts of the pandemic.^27 39^ However, our project review indicated that projects listed under the ‘COVID-19 control’ purpose or projects marked with the hashtag may not necessarily specify COVID-19 activities in their descriptions. Therefore, we compiled a list of COVID-19 keywords and searched for their presence in titles and descriptions among all the ECCE projects we identified for 2020 and 2021 (see keywords in online supplemental table S4c). We manually reviewed each project that fulfilled the search requirements and eliminated false positives. We compared the total ECCE aid in 2020 and 2021 with aid disbursements to ECCE projects with COVID-19 activities.

### Defining and estimating ECCE aid to projects in conflict-affected countries

There is extensive evidence demonstrating the pervasive negative effect of armed conflicts on children’s development.^40 41^ This adverse impact on children is partially mediated by the lack of access to ECCE.^13^ We estimated ECCE aid invested in conflict-affected countries throughout the study period. Consistent with previous studies,^20^ we defined conflict-affected countries as those who were either in conflicts or in postconflict stages in the period between 2007 and 2021 and defined ‘conflict’ as major armed battles with at least 1000 battle-related deaths in 1 year.

We identified a country as being in its postconflict stage if its conflict occurred between 2004 and 2006 but ended before 2007. We used Uppsala University Conflict Database^42^ for battle-related deaths to identify conflict-affected countries. We also supplemented our list of conflict-affected countries with the World Bank’s classification of fragile and conflict-affected situations.^43^ We identified 28 conflict-affected countries within the study period (online supplemental table S6). We compared the average ECCE aid per child in conflict-affected countries with the corresponding amount in non-conflict-affected countries.

### Analysing ECCE aid

Using both the lower-bound and upper-bound estimates, we tracked levels and trends of ECCE aid in terms of total amount and per ECCE-age child between 2007 and 2021 at global, regional and country levels. We also assessed levels and trends by recipient countries grouped into low-income, lower-middle-income and upper-middle-income categories.

We identified the top donors and recipient countries, tracked the CRS sectors to which ECCE aid was allocated, and identified the channel agencies that were responsible for implementing ECCE aid projects in the field. In cases where aid projects had missing information regarding channel agencies, we substituted their missing information with the most frequently employed agency of the same donor throughout the study period. For donors who did not report channel agencies for any ECCE projects, we assumed that the donors implemented the projects themselves.

Additionally, we examined the flow types of ECCE aid. The CRS database categorises aid into official development assistance (ODA) grants, ODA loans, other official flows and private development finance. ODA grants are non-repayable funds to recipient countries, while ODA loans require repayment that offer favourable terms such as lower interest rates or extended repayment periods. Other official flows are aid transactions with developmental goals but do not meet the criteria of ODA grants or loans. We included ‘private development finance’ in our analysis due to its primary development-focused nature, as compared with commercial revenues.^26 44^

Furthermore, we calculated each donor’s ECCE aid as a percentage of its development assistance to education (DAE) disbursed during the SDG study years (2016–2021). We compared these percentages to UNICEF’s recommendation, which suggests that donors allocate a minimum of 10% of their DAE to the ECCE sector.^17^ For recipient countries with available data on ECCE gross enrolment rates in 2020,^45^ we matched each country’s rank in enrolment with that of country’s rank in cumulative ECCE aid per child received throughout the study period.

We also examine countries’ cumulative ECCE aid per child against their most recent available data on the percentages of young children (36–59 months) developmentally on track as measured by the Early Childhood Development Index (ECDI).^46^ The ECDI is a 10-item index summarising caregiver-reported information on children’s achievement of some universal developmental milestones across countries. A child is deemed ‘developmentally on track’ if the child is on track in at least three of the four ECDI domains, including literacy-numeracy, physical development, social-emotional development and learning. Within each domain, a child is considered to be on track if the child has at least half of the relevant skills.^47^ Seventy-nine LMICs have available data on the percentages of children developmentally on track.

This article presents the lower-bound estimates as the main findings in the text, while the online supplemental file 1 document presents the upper-bound results. All disbursements were expressed in constant 2020 US dollars. We used STATA V.18.0 for analyses.

## Results

### General levels and trends of ECCE aid

**Figure 1:**
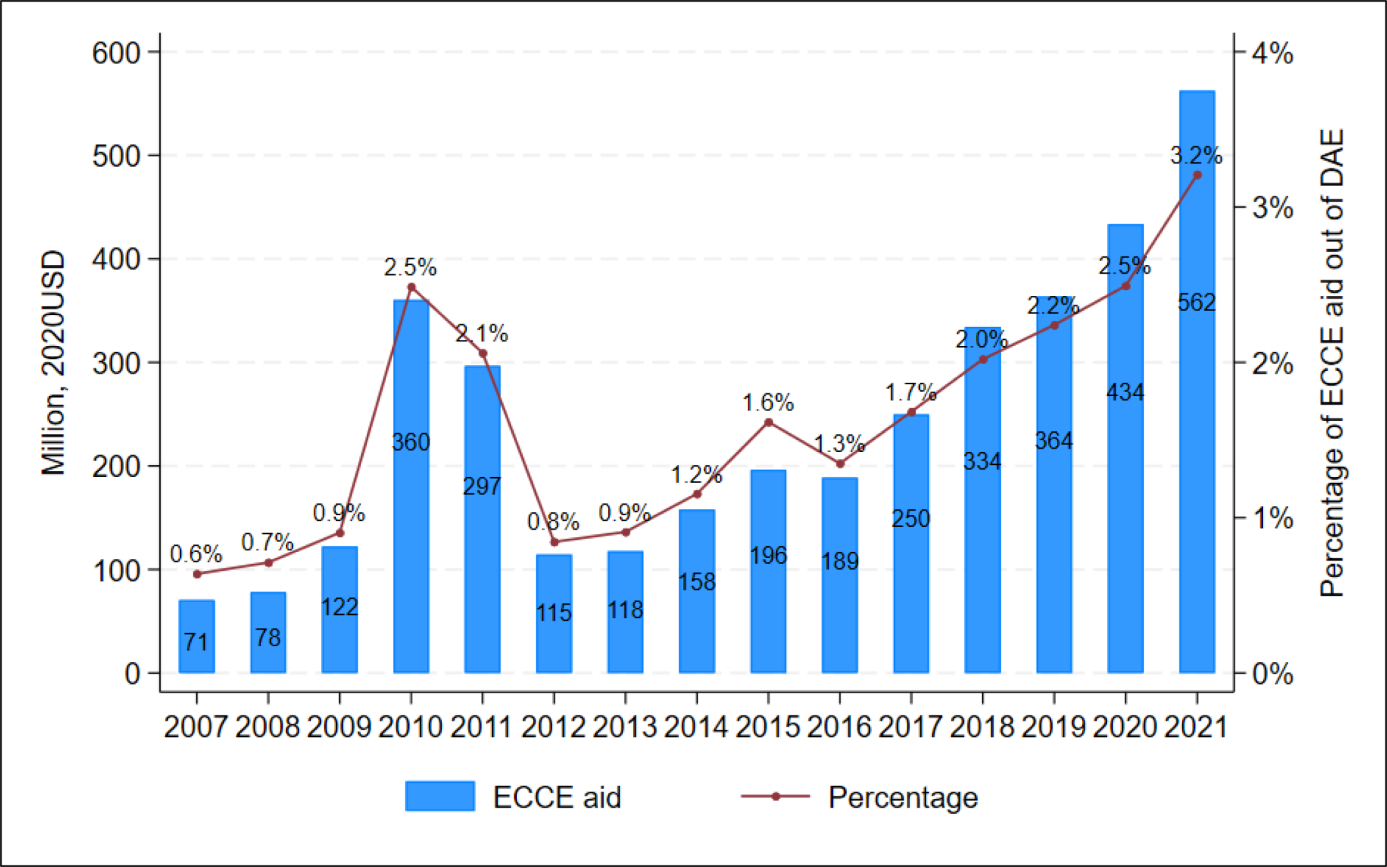
Annual early childhood care and education (ECCE) aid and its percentage of development assistance to education (DAE), 2007–2021. Data source: our estimates of the lower-bound ECCE aid.

**Figure 2:**
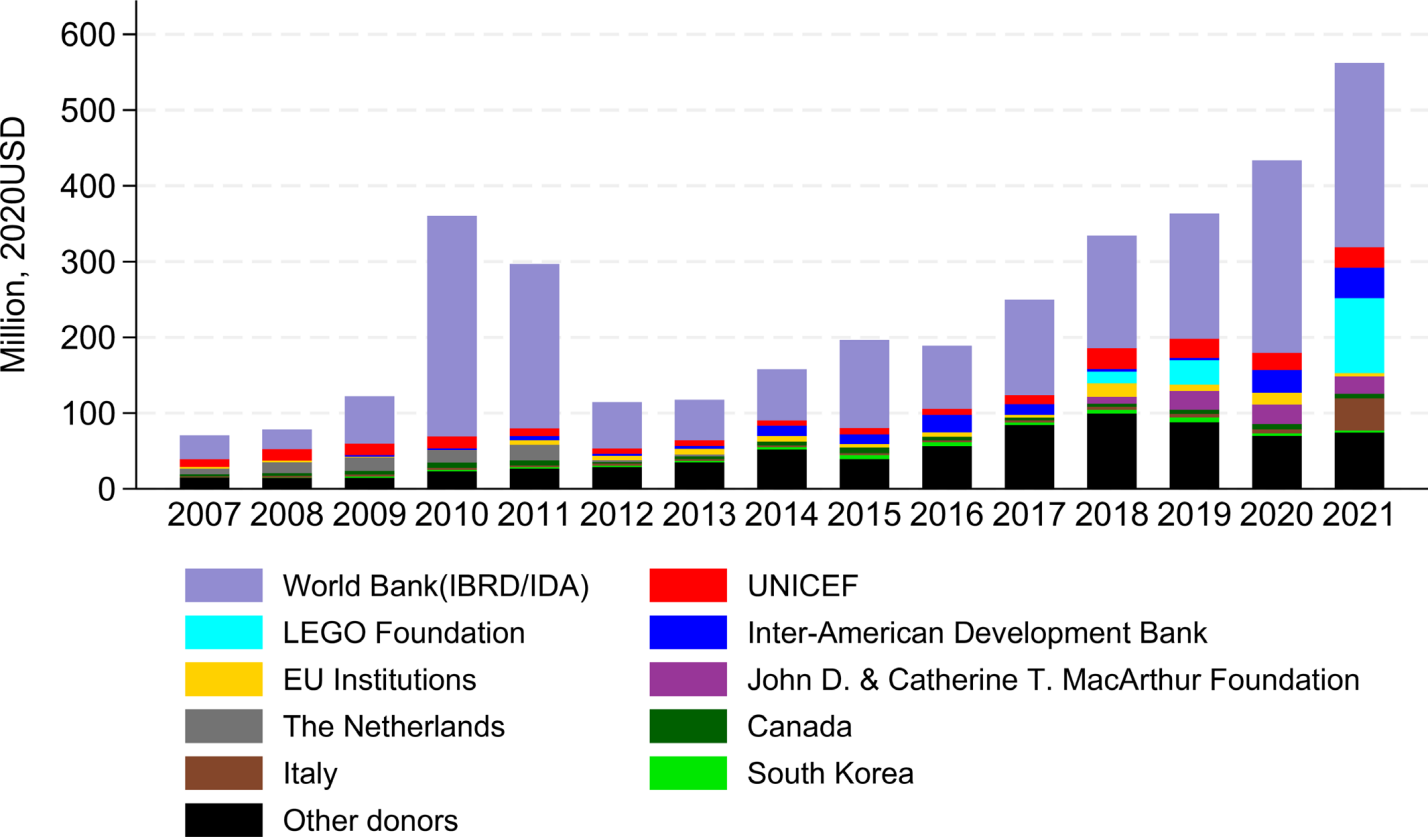
Annual early childhood care and education (ECCE) aid by top 10 donors and other donors, 2007–2021. Data source: our estimates of the lower-bound ECCE aid. EU, European Union; IBRD, International Bank for Reconstruction and Development; IDA, International Development Association.

Between 2007 and 2021, a total of US$3646 million in ECCE aid was allocated to 134 LMICs through 7977 projects. Figure 1 illustrates that the annual ECCE aid increased more than fivefold from US$71 million in 2007 to US$360 million in 2010. It then experienced a slight decrease to US$297 million in 2011, followed by a sharp decline to US$115 million in 2012. However, since 2013 it has been on an upward trajectory, reaching US$562 million in 2021. The peak in 2010 and 2011 was primarily due to large disbursements from the World Bank to Brazil and Turkey, amounting to US$243 million and US$181 million, respectively (figure 2). On average, ECCE aid accounted for 1.7% of the total DAE (US$2 13 279 million), with yearly percentages ranging from 0.6% in 2007 to 3.2% in 2021 (figure 1). Globally, ECCE aid per ECCE-age child increased substantially from US$0.1 in 2007 to US$0.8 in 2021, with an annual growth rate of 15.3% (table 1). For children enrolled in ECCE programmes, the aid amount per child rose from US$1.5 to US$3.4, implying an annual growth rate of 6%.

**Table 1:** ECCE aid per ECCE-age child by recipient country region and income group, 2007–2021

Category	Number of countries	2007	2008	2009	2010	2011	2012	2013	2014	2015	2016	2017	2018	2019	2020	2021	Average	Annual growth rate (%)
Global	134	0.1	0.1	0.2	0.5	0.4	0.2	0.2	0.2	0.3	0.3	0.4	0.5	0.5	0.6	0.8	0.4	15.3
Income group																		
LICs	27	0.1	0.1	0.1	0.1	0.1	0.1	0.1	0.2	0.1	0.3	0.2	0.5	0.4	0.8	0.6	0.2	18.7
LMs	55	0.1	0.1	0.2	0.2	0.2	0.1	0.2	0.2	0.2	0.2	0.2	0.3	0.4	0.4	0.6	0.2	15.4
UMs	52	0.2	0.1	0.3	1.4	1.1	0.3	0.2	0.2	0.5	0.3	0.7	0.8	0.7	1.0	1.3	0.6	15.0
Region																		
East Asia and Pacific	22	0.1	0.1	0.2	0.3	0.1	0.1	0.2	0.3	0.3	0.2	0.2	0.3	0.2	0.2	0.3	0.2	9.7
Europe and Central Asia	18	0.4	0.1	0.1	6.7	4.0	0.2	0.3	0.4	0.4	0.2	0.6	0.6	0.9	0.7	1.7	1.1	11.5
Latin America and the Caribbean	26	0.5	0.3	0.9	2.3	2.4	0.9	0.5	0.6	1.5	1.1	1.5	1.7	1.0	1.8	2.0	1.3	10.9
Middle East and North Africa	13	0.2	0.2	0.3	0.2	0.6	0.1	0.1	0.1	0.1	0.2	0.8	0.7	1.4	1.3	2.9	0.6	22.6
South Asia	8	0.0	0.1	0.0	0.1	0.1	0.1	0.1	0.1	0.1	0.1	0.1	0.2	0.4	0.2	0.3	0.1	19.1
Sub-Saharan Africa	47	0.1	0.1	0.1	0.1	0.1	0.1	0.1	0.2	0.1	0.3	0.2	0.5	0.5	0.8	0.7	0.3	18.4

Annual growth rate=(ECCE aid_2021/ECCE aid_2007)1/14–1.

ECCE, early childhood care and education; LIC, low-income country; LM, lower-middle-income country; UM, upper-middle-income country.

### ECCE aid by recipient countries’ income group

Across all country-income groups, there was a noticeable increase in total ECCE aid during the study period. However, the LIC group received less aid than the middle-income groups for most of the years, reaching a share of only 12.3% (US$449 million) of total ECCE aid. Of concern, between 2020 and 2021, LICs saw a reduction in their funding from US$103 million to US$77 million, while middle-income countries continued to see increasing amounts (online supplemental figure S2). When considering population size, LICs consistently received less ECCE aid per ECCE-age child before 2016 but started to catch up afterwards. However, from 2020 to 2021, LICs then saw a decrease from US$0.8 to US$0.6 per child, while lower-middle-income and upper-middle-income countries increased from US$0.4 to US$0.6 and from US$1.0 to US$1.3, respectively (table 1).

### ECCE aid by region of recipient countries

Over the span of 15 years, Latin America and the Caribbean region received the largest share of total ECCE aid (30.7%, US$1118 million), followed by sub-Saharan Africa (21.8%, US$794 million), East Asia and Pacific (14.0%, US$511 million), Middle East and North Africa (12.7%, US$461 million), South Asia (10.6%, US$386 million) and Europe and Central Asia (10.3%, US$377 million) (online supplemental figure S3). However, when considering population size, the South Asia, East Asia and Pacific and sub-Saharan Africa regions received a smaller amount of aid per child compared with the other regions. Notably, sub-Saharan Africa was the only region that experienced a decline in its aid per child from 2020 to 2021, dropping from US$0.8 to US$0.7 (table 1).

### ECCE aid by CRS sector

The educational sector received the largest share of ECCE aid, totaling US$3454 million, accounting for 94.7% of the total ECCE aid. It was followed by the social and economic infrastructure sector, which received US$149 million (4.1%), the emergency and humanitarian sector with US$18 million (0.5%), the health sector with US$9 million (0.3%) and other sectors with US$17 million (0.5%). Notably, the social-infrastructure and economic-infrastructure sector gradually increased its annual share from 2.3% in 2007 (US$2 million) to 10.1% in 2021 (US$57 million) (online supplemental figure S4), with the Inter-American Development Bank and the World Bank each contributing US$60 million during the study period.

Within the CRS emergency and humanitarian sector, a total of 149 ECCE projects were funded over the study period. The EU Institutions, as a single donor category in CRS, was the most prominent donor, contributing US$5 million, followed by UNICEF with US$4 million, and the USA with US$2 million. These projects were primarily focused on material-relief assistance and emergency-support services, including the provision of food assistance in ECCE settings, establishing ECCE classes, training teaching personnel for young refugee children and offering psychosocial support to preschool-aged children in affected areas. However, when comparing the total aid disbursements to the CRS emergency and humanitarian sector across the study period (US$477 047 million), the ECCE projects with humanitarian characteristics (US$18 million) appeared almost negligible.

### ECCE aid by implementation channel

Of the 7977 ECCE projects, 1976 (24.8%) lacked information on implementation channels, amounting to US$714 million (19.6%) over the study period. Among projects with missing channels, UNICEF contributed 1851 projects. We replaced missing channel data using the method described above. For UNICEF, we treated the missing data as self-implemented projects. After replacing the missing values, it was found that the governments of recipient countries played the most significant role in implementing ECCE aid-funded projects on the ground, accounting for 60% (US$2185 million) of total ECCE aid. Non-governmental organisations (NGOs) and multilateral organisations were the second and third largest channels, implementing 20.8% (US$757 million) and 11.8% (US$429 million) of total ECCE aid, respectively (online supplemental figure S5). The relatively large proportion (60%) of ECCE aid implemented by governments is unsurprising, given that the World Bank contributed 53.3% of the total ECCE aid over the study period, with nearly all its aid directed to recipient governments.

### ECCE aid by flow type

Throughout the study period, the other official flows, defined as aid transactions with developmental goals but that do not meet the criteria of ODA grants or loans, constituted 41.6% (US$1516 million) of total ECCE aid. They were followed by ODA grants at 31.7% (US$1156 million), ODA loans at 14.2% (US$518 million) and private development finance at 12.5% (US$456 million). By country-income groups, ODA grants and loans accounted for the largest proportion of funds allocated to low-income and lower-middle-income countries, representing 81.5% and 74.5%, respectively. In contrast, 76.9% of the total ECCE aid for upper-middle-income countries was in the form of other official flows. It is noteworthy that private developmental finance steadily increased since 2013, accounting for 25.4% (US$143 million) of the ECCE aid in 2021 (online supplemental figure S6).

### ECCE aid by donor

In the CRS database, the number of donors reporting any type of aid to the 134 LMICs increased from 42 in 2007 to 138 in 2021, while the number of donors reporting ECCE aid rose from 23 to 53 over the same period, partially contributing to the upward trend in ECCE aid. The World Bank was the leading donor with a cumulative aid disbursement of US$1944 million, accounting for 53.3% of total ECCE aid disbursed by all donors (figure 2). Moreover, the World Bank financed nine out of the 10 projects with the largest project values, including a cumulative total of US$215 million to three lower-middle-income countries (Vietnam, Indonesia and Morocco) and US$603 million to three upper-middle-income countries (Brazil, Turkey and Mexico) (table 2). Together with the World Bank, the top 10 donors contributed 80% (US$2922 million) of the total ECCE aid. Among them, eight donors have available aid data between 2007 and 2021, including the World Bank, UNICEF, Inter-American Development Bank, EU Institutions, The Netherlands, Canada, Italy and South Korea, contributing 74% (US$2693 million) of total ECCE aid. The other two donors—LEGO Foundation and John D. & Catherine T. MacArthur Foundation—started reporting to CRS in 2018 and 2017, respectively. These two donors cumulatively contributed 6% (US$229 million) of total ECCE aid.

**Table 2:** Top 10 ECCE aid projects with the largest aid disbursements

Project title	ECCE activity	Disbursement (US$, million)	Recipient (income group)	Donor
Rio de Janeiro municipality fiscal consolidation for efficiency and growth development policy loan	Operate 10 preschool programmes to target low-income neighbourhoods	177.7	Brazil (UM)	World Bank
Restoring equitable growth and employment programmatic development policy loan	Hire 15 000 new preschool teachers and launch universal preschool education in 32 provinces	138.6	Turkey (UM)	World Bank
School readiness promotion project	Improve school readiness for children aged 5 years who are most vulnerable to failing in a school environment	86.8	Vietnam (LM)	World Bank
Mexico school-based management project	Reduce drop-out, repetition and failure rates among participating schools through the improvement of early childhood education	81.0	Mexico (UM)	World Bank
Second restoring equitable growth and employment programmatic development policy loan	Expansion of preschool education to 25 provinces	80.6	Turkey (UM)	World Bank
Programme to support the national early childhood plan and the policy for universalisation of early childhood education	Universalisation of early childhood education	67.2	Argentina (UM)	Inter-American Development Bank
Early childhood education and development project	Prepare poor children for primary school through an integrated early childhood education and development system	65.0	Indonesia (LM)	World Bank
Recife swap education and public management	Expand coverage of improved early child education and create conditions more conducive to learning in fundamental education	64.7	Brazil (UM)	World Bank
Improving early childhood development outcomes in rural Morocco	Improve access to quality early childhood development services, including early childhood education, in rural areas	63.6	Morocco (LM)	World Bank
Reducing inequality of educational opportunity project	Support to build capacity of early childhood education facilitators, supervisors and coordinators.	60.4	Mexico (UM)	World Bank

ECCE, early childhood care and education; LM, lower-middle-income country; UM, upper-middle-income country.

However, when considering each donor’s ECCE aid as a percentage of its total educational aid (DAE) disbursement across the SDG study years (2016–2021), the DAC member countries, on average, allocated only 1.9% of DAE to the ECCE sector. Greece (18.5%) was the sole DAC member country whose allocation exceeded the UNICEF-recommended 10% threshold. In contrast to the DAC member countries, multilateral institutions allocated an average of 9.3%, with UNICEF leading at 25.6% (online supplemental figure S7).

### ECCE aid by recipient country

**Figure 3:**
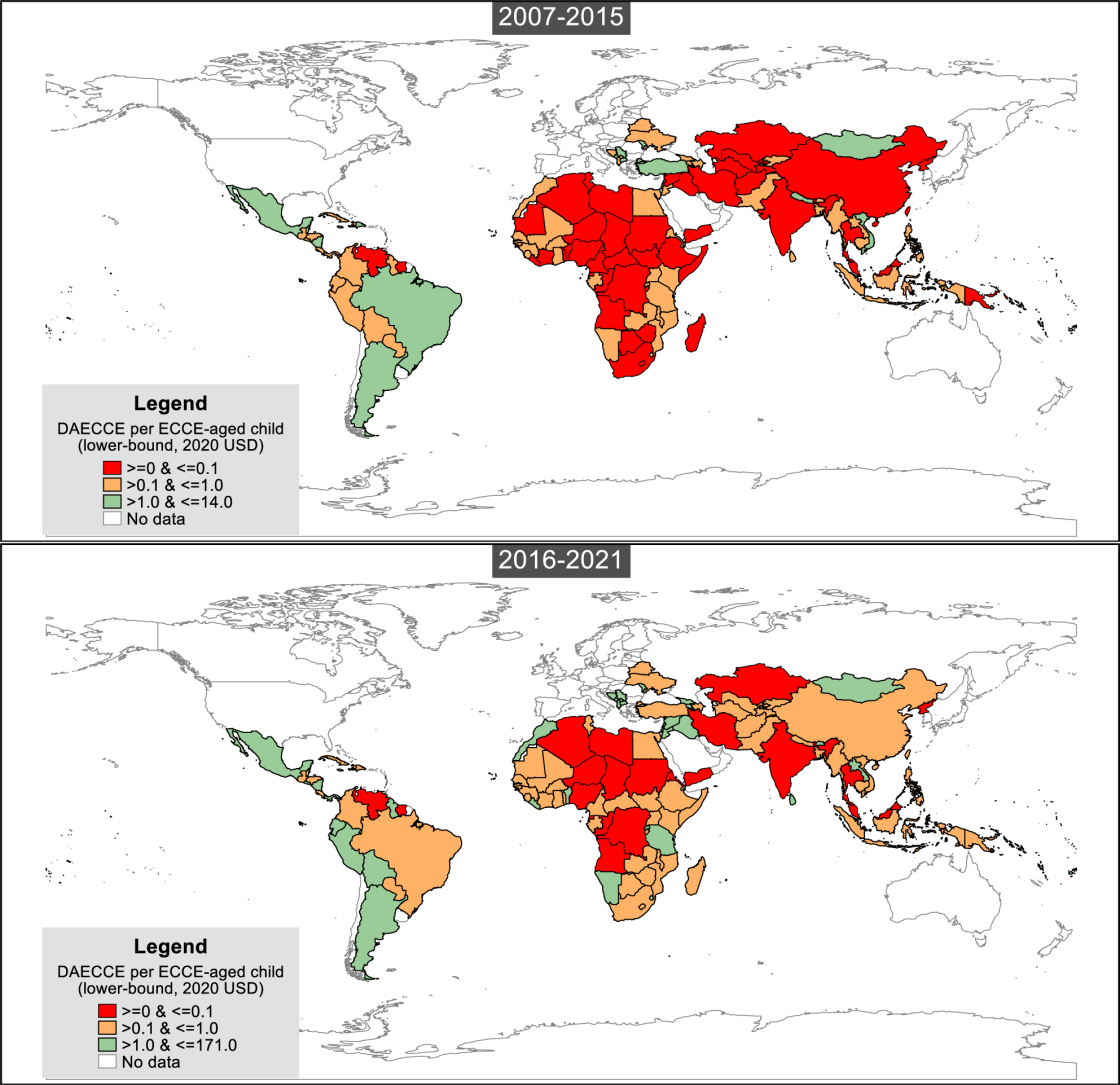
Average early childhood care and education (ECCE) aid per child by year group (2007–2015 versus 2016–2021). Data source: our estimates of the lower-bound ECCE aid.

Out of the 134 LMICs, 129 received ECCE aid directly from donors, while Grenada, Libya, Saint Lucia, Saint Vincent and the Grenadines and Suriname obtained ECCE aid through allocations of related regional or bilateral funds. Aggregated across the 15 study years, the top 10 recipients were middle-income countries, with Brazil leading with a total receipt of US$394 million. When considering the progress in ECCE aid per child, before the SDG study years (2007–2015), 52 recipient countries, half of which were in sub-Saharan Africa, received an average of <US$0.1. However, the number decreased to 23 during the SDG study period (2016–2021), with nine sub-Saharan Africa countries receiving <US$0.1 (figure 3). See online supplemental table S7 for each country’s yearly ECCE aid per child.

**Figure 4:**
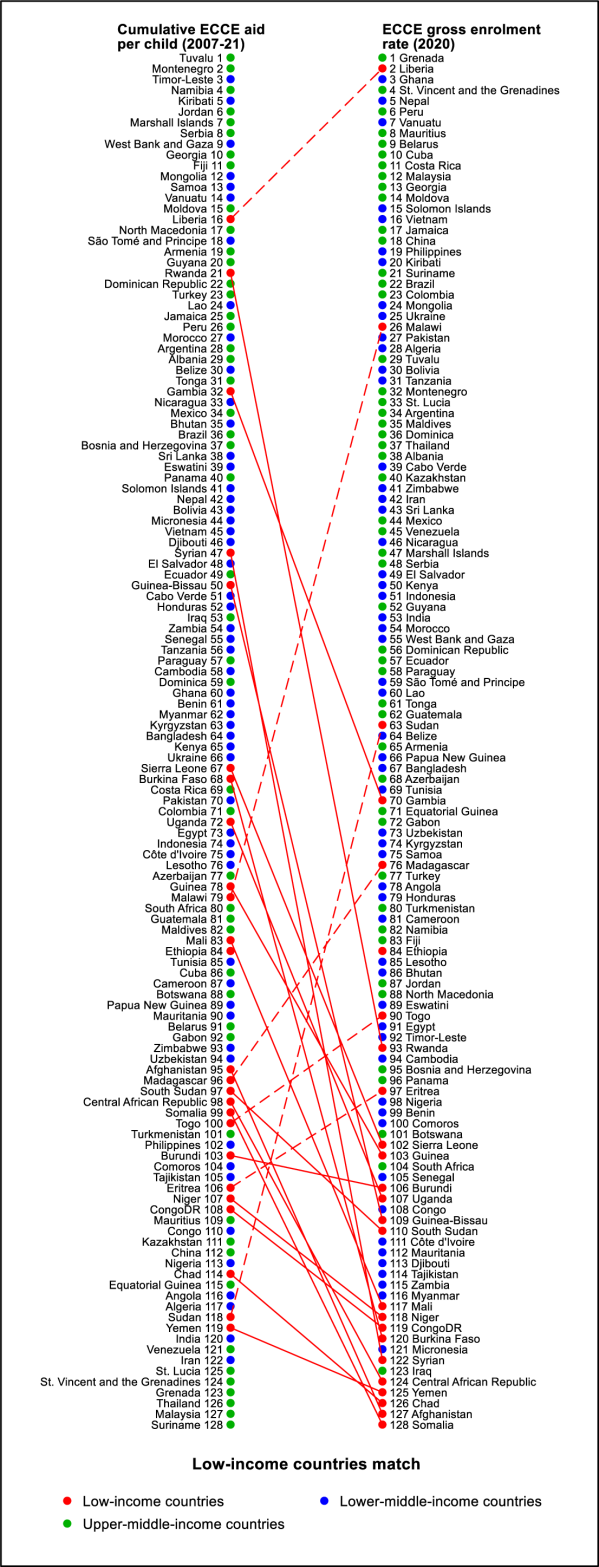
Low-income countries’ match between ranks in early childhood care and education (ECCE) aid per child and enrolment rate. Data source: UNESCO Institute for Statistics. School enrolment, preprimary (% gross). Our estimates of the lower-bound ECCE aid per child.

In figure 4 and online supplemental figures S8 and S9, we ranked 128 countries with available data on ECCE enrolment rates in 2020 in decreasing order on the right and ranked countries’ cumulative ECCE aid per child throughout the study period in a decreasing order on the left. Among the 25 low-income recipient countries in the lists, only six had a higher rank on the enrolment list compared with their ranking on the aid list. Notably, Sudan had the largest ranking gap among the six countries, ranking 63rd in terms of enrolment but 118th in aid. By contrast, Syria represented the other end of the spectrum, ranking 122nd in terms of enrolment rate but 47th in aid received.

**Figure 5:**
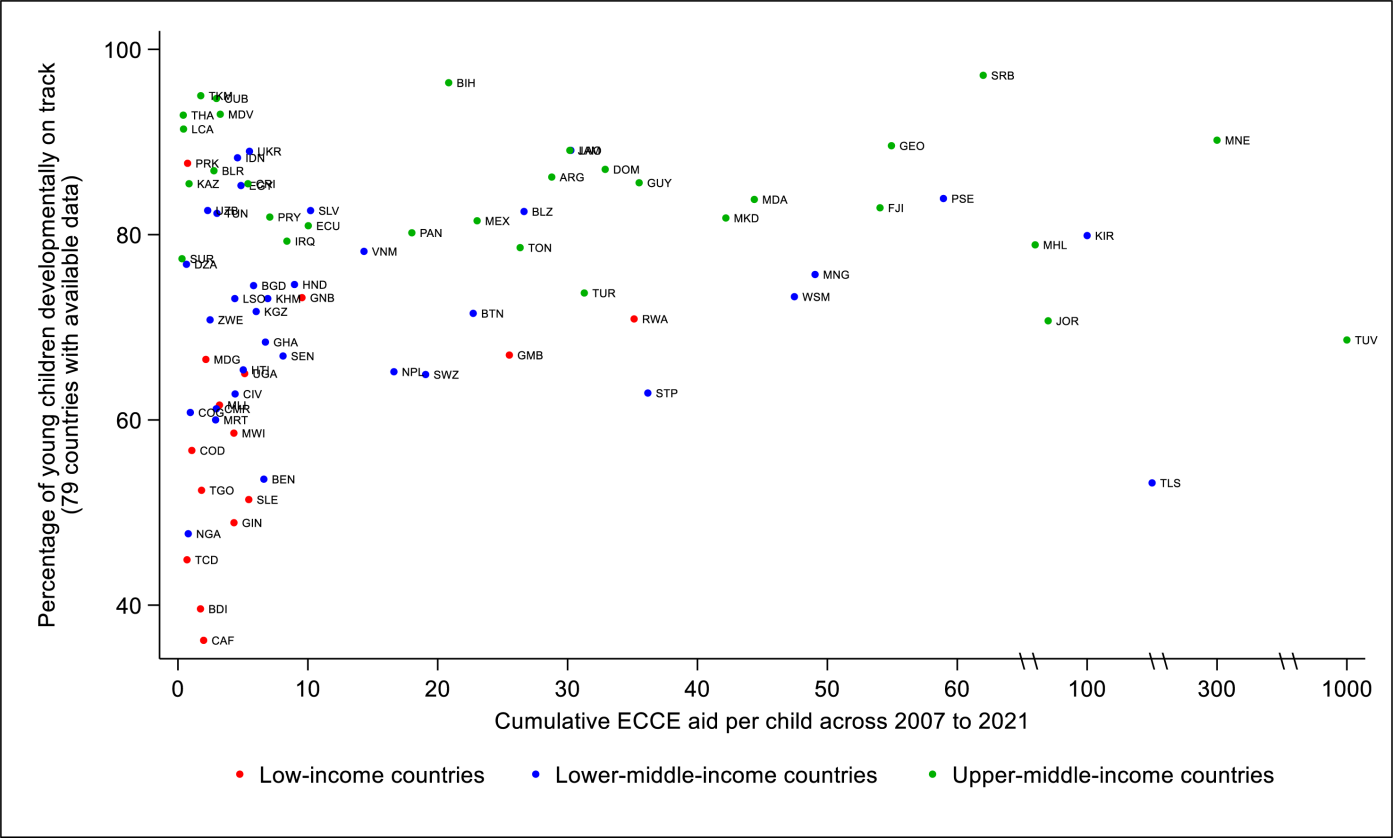
Cumulative early childhood care and education (ECCE) aid per child versus the percentage of young children (36–59 months) developmentally on track. Data source: UNICEF. Early childhood development—development status. Percentage of children on track in development status measured by Early Childhood Development Index. Our estimates of the lower-bound ECCE aid per child.

When examining countries’ cumulative ECCE aid per child against their percentages of children developmentally on track, most LICs with available data exhibit both low levels of aid and small percentages, forming a cluster in the bottom left corner of figure 5.

### ECCE aid with COVID-19 activities

ECCE aid allocated to projects with COVID-19 activities totaled US$87 million, with a decrease from US$50 million in 2020 to US$37 million in 2021, representing 11.4% and 6.6% of each year’s ECCE aid, respectively. The World Bank contributed the largest amount of ECCE aid to projects encompassing COVID-19 activities, constituting 9.3% (US$46 million) of its total ECCE aid over 2020 and 2021. Notably, the LEGO Foundation allocated US$36.4 million to ECCE projects including COVID-19 activities, accounting for 36.8% of its total ECCE aid over the 2 years. For recipient countries, Mexico received the largest amount of COVID-19-related ECCE aid, totaling US$40 million, which accounted for 94.1% of its total ECCE aid received during the 2 years. Panama and Kenya followed, receiving US$6 million and US$4 million, respectively, which represented 89.8% and 37.9% of their respective ECCE aid received during the same period (online supplemental tables S8 and S9).

### ECCE aid to conflict-affected countries

During the 15-year study period, the average ECCE aid per child in conflict-affected countries remained strikingly lower compared with non-conflict-affected countries. Conflict-affected countries received an average of US$0.3 per child, which was only a quarter of what non-conflict-affected countries received (US$1.2 per child). While non-affected countries saw a consistent increase in aid per child, conflict-affected countries experienced a slower and fluctuating growth (online supplemental figure S10).

### General levels and trends of upper-bound ECCE aid

The upper-bound ECCE aid showed a similar trend pattern to the lower-bound estimates. It totaled US$18 180 million across the 15-year period, with the annual aid spanning from US$664 million (2007) to US$1990 million (2021). When considering population size, the aid per ECCE-age child increased approximately threefold from US$1.0 (2007) to US$2.8 (2021). See online supplemental tables S10–S13 and online supplemental figure S11–S18 for more information on the upper-bound estimates.

## Discussion

Using data from the CRS, we systematically estimated ECCE aid allocated to 134 LMICs between 2007 and 2021. Total disbursements for aid projects primarily aimed at supporting ECCE saw substantial growth, increasing from US$71 million in 2007 to US$562 million in 2021. Despite this encouraging growth, the total ECCE aid over the 15-year period (US$3646 million) represents only 1.7% of total educational aid allocated to the 134 LMICs. This figure falls significantly short of UNICEF’s recommendation that at least 10% of education aid be directed to ECCE. Throughout the study period, the World Bank was the main engine for international ECCE financing. However, since the World Bank allocated aid based on requests from its member governments,^48 49^ the relatively small proportion of ECCE aid within total education aid reflects the low priority given to ECCE financing by these governments.

Of particular concern, for most of the study years, LICs received a smaller amount of ECCE aid per child than lower-middle-income and upper-middle-income countries. Moreover, the low-income group experienced a decline in ECCE aid per child between 2020 and 2021, while the other two country income groups maintained a steadily increasing trend during the same period. Although LICs may have lower price levels for services compared with middle-income countries, which might somewhat mitigate the impact of the small amount of ECCE aid, the low level and downward trend in aid reaffirm the significant challenges these countries face in obtaining external resources to improve access and quality for their ECCE programmes.^14^ Furthermore, considering that LICs had a much lower ECCE enrolment rate even before the COVID-19 pandemic (21% vs 40% in lower-middle-income countries, and 55% in upper-middle-income countries) and children in these settings may be disproportionately affected by ECCE closures caused by the pandemic,^11^ it remains fundamental to increase ECCE aid to enhance children’s early learning opportunities in these resource-limited settings. It is particularly important to address the problem that countries in the most need receive the least funding (eg, countries with small percentages of young children developmentally on track received small amounts of ECCE aid).

While overall ECCE aid increased between 2020 and 2021, we did not observe a corresponding rise in aid for ECCE programmes incorporating activities to address COVID-19 challenges in ECCE settings or to prepare ECCE programmes for future global crises. Recent evidence indicates that the closure of ECCE programmes in 196 countries between 2020 and 2021 may have resulted in a potential loss of 19 billion person-days of ECCE instruction with a total of 11 million additional children estimated to have fallen ‘off track’ in their learning and development.^11^ Such losses create a cycle of cumulative disadvantage for large numbers of children in the poorest countries. Ensuring that children have stable access to quality ECCE services, especially during global crises, should be an essential part of the United Nations’ quality education agenda. To achieve this goal, more aid allocations should be made towards basic infrastructure and worker/teacher training for ECCE. Previous studies^50^ have identified that the deficiencies in access to basic sanitation and hygiene facilities were the two conditions that made young children vulnerable to infectious diseases in household settings. Although there is limited research on crisis preparedness in ECCE settings, donors should consider allocations to equip ECCE with adequate infrastructure in hygiene/sanitation and especially online learning in future aid projects.

Despite studies showing that online learning during home confinement is positively associated with children’s development,^51^ the percentage of school-age children with internet access is deeply concerning—only 6% of children aged 3–17 years in LICs have access to the internet, compared with 60% in upper-middle-income countries.^52^ This disparity represents a significant opportunity for both governments and international donors to promote ECCE and foster long-term socioeconomic development by investing in digital infrastructure for young children and cultivating a generation with adequate digital literacy. Additionally, ensuring an adequate number of capable ECCE workers/teachers is essential for delivering quality at scale.^16^ However, during the study period, we only observed US$493 million disbursements (13.5% of the total ECCE disbursements) with teacher-training components. The full potential of ECCE aid in building a resilient ECCE system against global crises is more likely to be realised when donors collaborate with recipient governments to integrate strong teacher training and support components into aid projects.

Furthermore, our study unveiled a concerning trend in ECCE aid to conflict-affected countries and underscored a significant gap in integrating ECCE education into the emergency and humanitarian aid spectrum. Exposure to armed conflict has been extensively researched and is known to adversely impact early childhood development.^13 40 41^ It has been estimated that five consecutive years of exposure to armed conflict can lead to a 10.4% decrease in the probability of a child being developmentally on track.^13^ Despite this clear evidence, our study found that ECCE aid per child in conflict-affected countries was only a quarter of that in non-conflict-affected countries. Although our calculations confirmed that conflict-affected countries generally received less aid per capita than their counterparts, the low level of ECCE aid per child still represents an important opportunity for international donors to continue contributing to the goal of universal access to ECCE by 2030 by increasing investments in children in conflict-affected settings. Furthermore, we observed that ECCE was largely ignored in the profile of donor’s humanitarian aid projects. Given that many emergencies reflect underlying development crises facing LMICs,^53^ we issue a strong call for donors to shift their humanitarian aid philosophy from the United Nations’ definition of ‘life-saving’ to ‘quality life-saving’ and place greater emphasis on mitigating toxic stress on children through ECCE programmes in the design of humanitarian aid.

While our study focused on donors’ contributions to ECCE, we note the importance of investing in the other four components of Nurturing Care, as well as a wide range of socioeconomic programmes that influence early childhood development, such as poverty alleviation and family social protection. The intentional educational component embedded in ECCE programmes though makes them unique in introducing young children to educational environments outside the home, fostering essential socioemotional skills for future social life, and preparing them academically for success in primary education. Furthermore, we acknowledge the primary role of governments and households in ECCE spending, the global increasing trend of ECCE enrolment as well as the adjustment of the number of years included for ECCE in many LMICs (either increased or decreased) during the study period. Our future research will investigate the associations between investments in ECCE (including governments and household spending) and the coverage and the years included for ECCE in LMICs.

Our study has limitations. First, due to limited information, we did not include ECCE aid contributed by some major emerging economies, such as China and Brazil, as well as by some private entities (eg, NGOs and private foundations) that did not report to CRS. It has been shown that NGOs and private foundations have been playing important roles in financing ECCE. Our estimates show that they have significantly increased ECCE aid from US$24 million in 2016 to US$143 million in 2021. Second, the keyword-search strategy could not capture all projects on ECCE aid because of either the imperfect sensitivity of the strategy or typos and errors in project descriptions reported by donors. However, our validation test estimated that the keyword search strategy probably missed about 2.6% of projects (0.04% of funds), indicating that our findings are robust. Third, our allocation strategies in the upper-bound estimates lack sufficient empirical evidence. Due to limited information at the activity level, we allocated aid to a country’s ECCE sector by multiplying project values by the country’s percentage of ECCE-age population. Aid from projects involving both ECCE and non-educational activities was not allocated. This approach may introduce bias, either due to a country’s skewed investment in education beyond ECCE or a biased estimation of the ECCE-age population size, for instance, in cases of international migration due to conflicts.

## Conclusion

We note three key lessons to be drawn from this study. First, given the firm evidence that investing in ECCE is a highly effective approach to ease children’s burden caused by disadvantaged contexts,^14 54 55^ the necessity of prioritising ECCE in the educational financing framework should be clearly advocated to the international donor community. Although ECCE aid disbursements increased throughout our study period, LICs received a much smaller amount than their richer counterparts and experienced a decline in funds from 2020 to 2021. International donors should adjust their preferences and allocate more resources to support ECCE services in the most resource-limited settings.

Second, there had been a decline in ECCE aid towards projects with COVID-19 activities, reflecting a diminishing effort in preparing children for current and further global crises. Given the unaffordable losses in children’s development potential due to ECCE programme closures,^11^ the donor community should innovate their funding mechanisms by emphasising activities aimed at mitigating the adverse impact of infectious disease epidemics and making ECCE programmes well-prepared for the future challenges, like the looming climate crisis.

Third, conflict-affected countries received much less investment compared with more stable countries. While donors may prioritise providing aid in countries with stable political and socioeconomic environments, it is crucial to acknowledge the peacebuilding role of ECCE programmes in protecting human and social capital for future development.^56^ Therefore, we strongly urge donors to significantly increase their ECCE aid in such settings to enhance children’s resilience and contribute to addressing the underlying developmental challenges in unstable situations.

## Supplementary material

10.1136/bmjgh-2024-015991 online supplemental file 1

## Data Availability

Data are available in a public, open access repository.
