# Supplementary material for: Global development assistance for early childhood care and education in 134 low- and middle-income countries, 2007–2021
Source: BMJ Glob Health. 2024 Nov 20;9(11):e015991. doi: 10.1136/bmjgh-2024-015991 (PMC11580267; doi:10.1136/bmjgh-2024-015991)
Supplement: online supplemental file 1 [file bmjgh-9-11-s001.pdf]

Supplementary Online Content

Global development assistance for early childhood care and education in 134 low- and middle-income countries, 2007-2021

Yiqun Luan[1], Dominic Hodgkin[2], Jere R. Behrman[3], Alan Stein[4,5,6], Linda M. Richter[7,8], Jorge Cuartas[9,10], Chunling Lu[11,12*]

1. Heller School for Social Policy and Management, Brandeis University, Waltham, MA, USA
2. Schneider Institutes for Health Policy and Research, Heller School for Social Policy and Management, Brandeis University, Waltham, MA, USA
3. Department of Economics, University of Pennsylvania, Philadelphia, PA, USA
4. Blavatnik School of Government, University of Oxford, Oxford, UK
5. MRC/Wits Rural Public Health and Health Transitions Research Unit (Agincourt), Faculty of Health Sciences, School of Public Health, University of the Witwatersrand, Johannesburg, South Africa
6. Africa Health Research Institute, Durban, KwaZulu-Natal, South Africa
7. DSI-NRF Centre of Excellence in Human Development, University of Witwatersrand, Johannesburg, South Africa
8. Stellenbosch Institute for Advanced Study (STIAS), Stellenbosch, South Africa
9. Department of Applied Psychology, New York University, New York, NY, USA
10. Centro de Estudios sobre Seguridad y Drogas (CESED), Universidad de los Andes, Bogotá, Colombia
11. Division of Global Health Equity, Brigham & Women's Hospital, Boston, MA, USA
12. Department of Global Health and Social Medicine, Harvard Medical School, Boston, MA, USA

*Chunling Lu, Ph.D. (corresponding author)
Division of Global Health Equity, Brigham & Women's Hospital
Department of Global Health and Social Medicine, Harvard Medical School
641 Huntington Ave, Boston, Massachusetts 02115, USA
Phone : (617) 521-3369, Fax : (617) 521-3393
E-mail : chunling_lu@hms.harvard.edu

Table S1. 134 low- and middle-income recipient countries included in this study

Low-income countries (27):
Afghanistan, Burkina Faso, Burundi, Central African Republic, Chad, Democratic Republic of the Congo, Eritrea, Ethiopia, Gambia, Guinea, Guinea-Bissau, Democratic People's Republic of Korea, Liberia, Madagascar, Malawi, Mali, Mozambique, Niger, Rwanda, Sierra Leone, Somalia, South Sudan, Sudan, Syria, Togo, Uganda, Yemen

Lower-middle-income countries (55):
Algeria, Angola, Bangladesh, Belize, Benin, Bhutan, Bolivia, Cabo Verde, Cambodia, Cameroon, Comoros, Congo, Côte d'Ivoire, Djibouti, Egypt, El Salvador, Eswatini, Ghana, Haiti, Honduras, India, Indonesia, Iran, Kenya, Kiribati, Kyrgyzstan, Lao, Lesotho, Mauritania, Micronesia, Mongolia, Morocco, Myanmar, Nepal, Nicaragua, Nigeria, Pakistan, Papua New Guinea, Philippines, Samoa, Senegal, Solomon Islands, Sri Lanka, São Tomé and Principe, Tajikistan, Tanzania, Timor-Leste, Tunisia, Ukraine, Uzbekistan, Vanuatu, Vietnam, West Bank and Gaza, Zambia, Zimbabwe

Upper-middle-income countries (52):
Albania, Argentina, Armenia, Azerbaijan, Belarus, Bosnia and Herzegovina, Botswana, Brazil, China, Colombia, Costa Rica, Cuba, Dominica, Dominican Republic, Ecuador, Equatorial Guinea, Fiji, Gabon, Georgia, Grenada, Guatemala, Guyana, Iraq, Jamaica, Jordan, Kazakhstan, Kosovo, Lebanon, Libya, Malaysia, Maldives, Marshall Islands, Mauritius, Mexico, Moldova, Montenegro, Namibia, North Macedonia, Panama, Paraguay, Peru, Serbia, South Africa, St. Lucia, St. Vincent and the Grenadines, Suriname, Thailand, Tonga, Turkey, Turkmenistan, Tuvalu, Venezuela

Recipient countries excluded (21):
Anguilla[†], Antigua and Barbuda[⁋], Barbados[⁋], Chile[⁋], Cook Islands[†], Croatia[⁋], Mayotte[†], Montserrat[†], Nauru[⁋], Niue[†], Oman[⁋], Palau[⁋], Saint Helena[†], Saudi Arabia[⁋], Seychelles[⁋], St. Kitts and Nevis[⁋], Tokelau[†], Trinidad and Tobago[⁋], Turks and Caicos Islands[⁋], Uruguay[⁋], Wallis and Futuna[†]

†. No World Bank 2020 income classification[1].
⁋. Categorized as high-income according to World Bank 2020 income classification.

Reference:

1. World Bank. World Bank country and lending groups. https://datahelpdesk.worldbank.org/knowledgebase/articles/906519-world-bank-country-and-lending-groups. (Accessed date: August 28, 2024)

Table S2a. OECD-DAC country donors and years with available data on aid to 134 LMICs between 2007 and 2021

Donor	Year with available aid data	Donor	Year with available aid data
Australia	2007-2021	Japan	2007-2021
Austria	2007-2021	Korea	2007-2021
Belgium	2007-2021	Lithuania	2014-2021
Canada	2007-2021	Luxembourg	2007-2021
Czech Republic	2011-2021	Netherlands	2007-2021
Denmark	2007-2021	New Zealand	2007-2021
Estonia	2013-2021	Norway	2007-2021
EU Institutions	2007-2021	Poland	2013-2021
Finland	2007-2021	Portugal	2007-2021
France	2007-2021	Slovak Republic	2013-2021
Germany	2007-2021	Slovenia	2010-2021
Greece	2007-2021	Spain	2007-2021
Hungary	2014-2021	Sweden	2007-2021
Iceland	2011-2021	Switzerland	2007-2021
Ireland	2007-2021	United Kingdom	2007-2021
Italy	2007-2021	United States	2007-2021

Table S2b. Non-OECD-DAC country donors and years with available data on aid to 134 LMICs between 2007 and 2021

**Donor**	**Year with available aid data**	**Donor**	**Year with available aid data**
Azerbaijan	2014-2020	Malta	2015-2021
Bulgaria	2015-2021	Monaco	2021
Chinese Taipei	2015-2021	Qatar	2019-2021
Croatia	2014-2021	Romania	2014-2021
Cyprus	2014-2015, 2018-2021	Saudi Arabia	2015-2021
Israel	2015-2021	Thailand	2015-2021
Kazakhstan	2013-2021	Timor-Leste	2014-2015
Kuwait	2010-2021	Turkey	2015-2021
Latvia	2015-2021	United Arab Emirates	2009-2021
Liechtenstein	2015-2021		

Table S2c. Multilateral donors and years with available data on aid to 134 LMICs between 2007 and 2021

Donor	Year with available aid data	Donor	Year with available aid data
Adaptation Fund	2010-2021	International Development Association	2007-2021
African Development Bank	2007-2021	International Finance Corporation	2012-2021
African Development Fund	2007-2021	International Investment Bank	2019-2021
Arab Bank for Economic Development in Africa	2011-2015, 2019-2020	International Labour Organization	2012-2021
Arab Fund (AFESD)	2008-2021	Islamic Development Bank	2007-2021
Asian Development Bank	2007-2021	Montreal Protocol	2015
Asian Infrastructure Investment Bank	2016-2021	New Development Bank	2021
Black Sea Trade & Development Bank	2020-2021	Nordic Development Fund	2009-2021
Caribbean Development Bank	2015-2021	North American Development Bank	2021
Center of Excellence in Finance	2016-2019	OPEC Fund for International Development	2009-2021
Central American Bank for Economic Integration	2020-2021	OSCE	2010-2021
Central Emergency Response Fund	2017-2021	United Nations Conference on Trade and Development	2020-2021
Climate Investment Funds	2012-2021	United Nations Industrial Development Organization	2020-2021
Council of Europe Development Bank	2010-2021	UN Institute for Disarmament Research	2019-2021
Development Bank of Latin America	2017-2021	UN Peacebuilding Fund	2007-2021
European Bank for Reconstruction and Development	2009-2021	UNAIDS	2007-2021
Eurasian Fund for Stabilization and Development	2020-2021	UN Capital Development Fund	2020-2021
Food and Agriculture Organization	2013, 2017-2019, 2021	UNDP	2007-2021
Global Alliance for Vaccines and Immunization	2007-2021	UNECE	2008-2017
Global Environment Facility	2007-2021	UNEP	2015-2016
Global Fund	2007-2021	UNFPA	2007-2021
Global Green Growth Institute	2013-2021	UNHCR	2011-2021
Green Climate Fund	2015-2021	UNICEF	2007-2021
IDB Invest	2016-2021	UNRWA	2007-2021

IFAD	2007-2021	WFP	2008-2021
IMF (Concessional Trust Funds)	2007-2021	World Health Organization	2009-2021
Inter-American Development Bank	2007-2021	World Tourism Organization	2016-2020
International Atomic Energy Agency	2015-2021	WTO - International Trade Centre	2020-2021
International Bank for Reconstruction and Development	2007-2021	WHO-Strategic Preparedness and Response Plan	2021

Table S2d. Private donors and years with available data on aid to 134 LMICs between 2007 and 2021

**Donor**	**Year with available data**	**Donor**	**Year with available data**
Arcadia Fund	2015-2021	Jacobs Foundation	2016-2021
Arcus Foundation	2015-2021	John D. & Catherine T. MacArthur Foundation	2017-2021
BBVA Microfinance Foundation	2017-2021	La Caixa Banking Foundation	2017-2021
Bernard van Leer Foundation	2017-2021	Laudes Foundation	2013-2021
Bezos Earth Fund	2020-2021	LEGO Foundation	2018-2019, 2021
Bill & Melinda Gates Foundation	2009-2021	Margaret A. Cargill Foundation	2016-2020
Bloomberg Family Foundation	2017-2021	MasterCard Foundation	2017-2021
Botnar Foundation	2017-2020	MAVA Foundation	2013-2021
Carnegie Corporation of New York	2017-2021	McKnight Foundation	2018-2020
Charity Projects Ltd (Comic Relief)	2013-2021	MetLife Foundation	2013-2020
Children's Investment Fund Foundation	2017-2021	Michael & Susan Dell Foundation	2016-2021
Citi Foundation	2017-2021	Norwegian Postcode Lottery	2018-2021
Conrad N. Hilton Foundation	2013-2021	Oak Foundation	2017-2021
David & Lucile Packard Foundation	2017-2021	Omidyar Network Fund, Inc.	2017-2020
Dutch Postcode Lottery	2016-2021	Open Society Foundations	2016-2020
Fondation Botnar	2021	People's Postcode Lottery	2016-2021
Ford Foundation	2017-2021	Rockefeller Foundation	2018-2021
Gatsby Charitable Foundation	2013-2020	Susan T. Buffett Foundation	2017-2020
Gordon and Betty Moore Foundation	2017-2021	Swedish Postcode Lottery	2017-2021
Grameen Crédit Agricole Foundation	2017-2020	UBS Optimus Foundation	2019-2021
H&M Foundation	2013-2021	Wellcome Trust	2017-2021
Howard G. Buffett Foundation	2017-2020	William & Flora Hewlett Foundation	2017-2020
IKEA Foundation	2015-2021	World Diabetes Foundation	2016-2021
International Commission on Missing Persons	2021	German Postcode Lottery	2021

Note:

1. The classification of donor types was based on DAC and CRS code list. https://www.oecd.org/dac/financing-sustainable-development/development-finance-standards/dacandcrscodelists.htm (Accessed date: August 28, 2024)

Table S3. CRS sectors used for searching ECCE aid projects

I. Social Infrastructure and Services: This main category relates essentially to efforts to develop the human resource potential of developing countries.		*II. Economic Infrastructure and Services: This major heading groups assistance for networks, utilities and services that facilitate economic activity.*	
**Sector name**	**Sector code**	**Sector name**	**Sector code**
Education	110	Transport & Storage	210
Education, Level Unspecified	111	Communications	220
Basic Education[†]	112	Energy	230
Secondary Education	113	Energy Policy	231
Post-Secondary Education	114	Energy generation, renewable sources	232
Health	120	Energy generation, non-renewable sources	233
Health, General	121	Hybrid energy plants	234
Basic Health	122	Nuclear energy plants	235
Non-communicable diseases (NCDs)	123	Energy distribution	236
Population Policies/Programmes & Reproductive Health	130	Banking & Financial Services	240
Water Supply & Sanitation	140	Business & Other Services	250
Government & Civil Society	150		
Government & Civil Society-general	151		
Conflict, Peace & Security	152		
Other Social Infrastructure & Services	160		
III. Production Sectors: This main heading groups contributions to all directly productive sectors.		*IV. Multisector/Cross-Cutting: This main heading includes support for projects which straddle several sectors.*	
**Sector name**	**Sector code**	**Sector name**	**Sector code**
Agriculture, Forestry, Fishing	310	General Environment Protection	410
Agriculture	311	Other Multisector	430
Forestry	312		
Fishing	313		
Industry, Mining, Construction	320		
Industry	321		
Mineral Resources & Mining	322		
Construction	323		
Trade Policies & Regulations	330		
Trade Policies & Regulations	331		
Tourism	332		
V. Commodity Aid and General Program Assistance		*VI. Action Relating to Debt*	

Sector name	Sector code	Sector name	Sector code
General Budget Support	510	Action Relating to Debt	600
Development Food Assistance	520		
Other Commodity Assistance	530		
VII. Humanitarian Aid: Humanitarian aid is assistance designed to save lives, alleviate suffering and maintain and protect human dignity during and in the aftermath of emergencies.		*VIII. Administrative Costs of Donors, Refugees in Donor Countries, Unallocated/Unspecified*	
**Sector name**	**Sector code**	**Sector name**	**Sector code**
Emergency Response	720	Administrative Costs of Donors	910
Reconstruction Relief & Rehabilitation	730	Refugees in Donor Countries	930
Disaster Prevention & Preparedness	740	Unallocated / Unspecified	998

†. We did not apply keyword search to projects reported under the "Early childhood education" purpose of the "Basic Education" sector.

Reference:

1. OECD. DAC and CRS code lists. https://www.oecd.org/dac/financing-sustainable-development/development-finance-standards/dacandcrscodelists.htm Accessed date: August 28, 2024.
2. OECD. DAC Working Party on Development Finance Statistics. Converged Statistical Reporting Directives for the Creditor Reporting System (CRS) and the Annual DAC Questionnaire. Annexes – modules A, B, and C (20 April 2021 edition). https://www.oecd.org/dac/financing-sustainable-development/development-finance-standards/data-collection-and-resources-for-data-reporters.htm Accessed date: August 28, 2024.

Table S4a. Keywords used to search for ECCE aid projects

Group 1: ECCE-specific keywords				
0 class	ECCE	Infant educat[†]	PIDI	Ready to learn
Anganwadi	ECD	Infant friendly learn	Play school	Reception class
CCD	ECE	Infant friendly school	Pre-academic	Reception year
Creche	Educa a Tu Hijo[†]	Infant friendly space	Pre-K	Sesame Street
Early brain	EEC	Infant friendly environ	Prekindergarten	Sustainable development goal 4.2
Early care	Grade 0	Infant learn	Preparatory year	Tahderiyyah[†]
Early child program	Grade R	Infant school	Preprimary	Year R
Early develop	ICDS	Kinder Catch-up Education (KCE)	Preschool	
Early educat[†]	Infant care	Kindergarten	PPE	
Early learn	Infant center	Mi Primer Empleo Digno[†]	Proyecto Integral de Desarrollo Infantil[†]	
Early year educat[†]	Infant develop	PEDAKOS[†]	R class	
Group 2: general care/education keywords				
Behavior develop	Child-to-child	Language	Nurturing learn	Psychological
Brain develop	Cognition	Learn and play	Physical develop	Psychosocial
Caring for child	Comprehensive care	Learn habit	Physical exercise	Readiness program
Center-based	Comprehensive develop	Learn opportunit[†]	Physical growth	Role-play
Child and family attention center	Day care	Learn potential	Physical well-being	Safe learn
Child and family care center	Day center	Learn space	Play activit[†]	Satellite center
Child and family center	Drop-in	Learn through play	Play group	Social interact
Child care	Educat[†]	Literacy	Play interact	Stimulation
Child center	Education game	Motor develop	Play-and-learn	Supervised neighborhood play
Child develop	Education television	Motor skill	Play-based	Verbal comprehen[†]
Child educat[†]	Emotion	Neurodevelop	Playful learn	Verbal mean
Child friendly learn	Initial educat[†]	Number concept	Plaything	
Child friendly space	Interactive learn	Nursery	Pre learn	
Child learn	Interactive play	Nurturing care	Pre-read	

Note:

1. We compiled keywords based on literature review listed in the "Reference".
2. We performed search with keywords in English and other major languages: Spanish, French, Portuguese, Italian, Dutch, German, Norwegian, and Swedish.
3. We performed keywords searching using (1) all lower case; (2) all upper case; (3) capitalize the first letter of each word; (4) capitalize the first letter of the term.
4. For terms consisting of two or more words, we also searched in their hyphenated forms, such as "safe-learn", and in the form without space if appliable, such as "childcare" for "child care".
5. For abbreviated keywords, such as ECE, we also searched them with hyphen, slash, parentheses, and space, such as "-ECE", "/ECE", "(ECE)", " ECE ".
†. We truncated words to represent their variant spellings, such as using "educat" to represent "educate", "educated", "education", and "educational".
‡. "Educa a Tu Hijo" is an ECCE program in Cuba; "Mi Primer Empleo Digno" is a public works Project in Bolivia, includes financing a daily stipend equivalent of Bs 500 per woman with children under six to cover daycare for children during training; "PEDAKOS" is an ECCE program in Kosovo; "Proyecto Integral de Desarrollo Infantil" is an ECCE program in Bolivia; "Tahderiyyah" is a preschool program supported by the UNICEF Country Office in the Philippines to reach disadvantaged children in remote.

Reference

1. Grantham-McGregor, S., Cheung, Y. B., Cueto, S., Glewwe, P., Richter, L., Strupp, B., & International Child Development Steering Group. (2007). Developmental potential in the first 5 years for children in developing countries. The lancet, 369(9555), 60-70.
2. Walker, S. P., Wachs, T. D., Gardner, J. M., Lozoff, B., Wasserman, G. A., Pollitt, E., ... & International Child Development Steering Group. (2007). Child development: risk factors for adverse outcomes in developing countries. *The lancet*, *369*(9556), 145-157.
3. Engle, P. L., Black, M. M., Behrman, J. R., De Mello, M. C., Gertler, P. J., Kapiriri, L., ... & International Child Development Steering Group. (2007). Strategies to avoid the loss of developmental potential in more than 200 million children in the developing world. *The lancet*, *369*(9557), 229-242.
4. Walker, S. P., Wachs, T. D., Grantham-McGregor, S., Black, M. M., Nelson, C. A., Huffman, S. L., ... & Richter, L. (2011). Inequality in early childhood: risk and protective factors for early child development. The lancet, 378(9799), 1325-1338.
5. Engle, P. L., Fernald, L. C., Alderman, H., Behrman, J., O'Gara, C., Yousafzai, A., ... & Global Child Development Steering Group. (2011). Strategies for reducing inequalities and improving developmental outcomes for young children in low-income and middle-income countries. *The Lancet*, *378*(9799), 1339-1353.
6. Black, M. M., Walker, S. P., Fernald, L. C., Andersen, C. T., DiGirolamo, A. M., Lu, C., ... & Lancet Early Childhood Development Series Steering Committee. (2017). Early childhood development coming of age: science through the life course. *The Lancet*, *389*(10064), 77-90.
7. Britto, P. R., Lye, S. J., Proulx, K., Yousafzai, A. K., Matthews, S. G., Vaivada, T., ... & Lancet Early Childhood Development Series Steering Committee. (2017). Nurturing care: promoting early childhood development. *The Lancet*, *389*(10064), 91-102.
8. Richter, L. M., Daelmans, B., Lombardi, J., Heymann, J., Boo, F. L., Behrman, J. R., ... & Lancet Early Childhood Development Series Steering Committee. (2017). Investing in the foundation of sustainable development: pathways to scale up for early childhood development. *The lancet*, *389*(10064), 103-118.
9. Sayre, R. K., Devercelli, A. E., Neuman, M. J., & Wodon, Q. (2015). Investing in early childhood development: review of the World Bank's recent experience.
10. Results for Development Institute (2016). Financing Early Childhood Development: An Analysis of International and Domestic Sources in Low- and Middle-Income Countries. https://r4d.org/resources/financing-early-childhood-development-analysis-international-domestic-sources/ Accessed date: May 10, 2021.
11. Arregoces, L., Hughes, R., Milner, K. M., Hardy, V. P., Tann, C., Upadhyay, A., & Lawn, J. E. (2019). Accountability for funds for Nurturing Care: what can we measure?. *Archives of disease in childhood*, *104*(Suppl 1), S34-S42.
12. RESULTS. Investing in Every Child's Early Years: World Bank Contributions. https://results.org/blog/investing_in_every_childs_early_years_world_bank_contributions/ Accessed date: May 28, 2020.
13. World Health Organization, United Nations Children's Fund, World Bank Group. Nurturing care for early childhood development: a framework for helping children survive and thrive to transform health and human potential. Geneva: World Health Organization; 2018. https://www.who.int/publications-detail-redirect/9789241514064 Accessed date: June 17, 2021.
14. United Nations Children's Fund, A World Ready to Learn: Prioritizing quality early childhood education, UNICEF, New York, April 2019. https://www.unicef.org/reports/a-world-ready-to-learn-2019 Accessed date: May 26, 2021
15. Aboud, F. E., & Yousafzai, A. K. (2015). Global health and development in early childhood. *Annual review of psychology*, *66*, 433-457.
16. Brown, T. W., van Urk, F. C., Waller, R., & Mayo-Wilson, E. (2014). Centre-based day care for children younger than five years of age in low-and middle-income countries. *Cochrane Database of Systematic Reviews*, (9).
17. Subramanian, S. (2019). India's Policy on Early Childhood Education: Lessons for a Gender-Transformative Early Childhood in India. Echidna Global Scholars Program, Policy

Paper. *Center for Universal Education at The Brookings Institution*.
18. Gustafsson-Wright, E., Smith, K., & Gardiner, S. (2017). *Public-private partnerships in early childhood development: the role of publicly funded private provision*. Working Paper]. Washington DC: Center for Universal Education at Brookings.
19. UNESCO. Global Education Monitoring Report 2020. Inclusion and Education: All Means All. https://en.unesco.org/gem-report/report/2020/inclusion Accessed date: August 28, 2024
20. UNESCO. International Standard Classification of Education (ISCED 2011). http://uis.unesco.org/en/topic/international-standard-classification-education-isced Accessed date: August 28, 2024
21. Brown, T. W., van Urk, F. C., Waller, R., & Mayo-Wilson, E. (2014). Centre-based day care for children younger than five years of age in low-and middle-income countries. Cochrane Database of Systematic Reviews, (9).
22. United Nations Educational, Scientific and Cultural Organization (UNESCO). (2020). Global education monitoring report 2020: Inclusion and education: All means all. 92310038.
23. O'Donnell, M., Ross, K., & Bourgault, S. (2021). A Review of Multilateral Development Banks' Investments in Childcare. CGD Policy Paper, 223.

Table S4b. Age-related terms

Child	0 to 3	3 to 5	6 month	Age 3
Kid	0 to 4	3 to 6	12 month	Age 4
Boy	0 to 5	3 to 7	24 month	Age 5
Girl	0 to 6	4 to 5	36 month	Age 6
Newborn	0 to 7	4 to 6	48 month	Age 7
Toddler	1 to 2	4 to 7	59 month	Age of 0
Infant	1 to 3	5 to 6	60 month	Age of 1
Infancy	1 to 4	5 to 7	26 week	Age of 2
1 year	1 to 5	6 to 7	52 week	Age of 3
2 year	1 to 6	Under 1	104 week	Age of 4
3 year	1 to 7	Under 2	156 week	Age of 5
4 year	2 to 3	Under 3	208 week	Age of 6
5 year	2 to 4	Under 4	256 week	Age of 7
6 year	2 to 5	Under 5	260 week	
7 year	2 to 6	Under 6	Age 0	
0 to 1	2 to 7	Under 7	Age 1	
0 to 2	3 to 4	0 month	Age 2	

Note:
1. We performed search with keywords in English and other major languages: Spanish, French, Portuguese, Italian, Dutch, German, Norwegian, and Swedish.
2. In addition to searching Arabic numerals, we replaced numbers with words, such as "under five" for "under 5" and searched: (1) all lower case; (2) all upper case; (3) capitalize the first letter of each word; (4) capitalize the first letter of the term.
3. For terms consisting of two or more words, we also searched in their hyphenated forms, such as "age-of-three".

Table S4c. COVID-19 keywords

Pandemic	Covid	Corona virus	Wuhan virus	Wuhan pneumonia
-Ncov	SARS-CoV-2	Mask		

Note:

1. We performed search with keywords in English and other major languages: Spanish, French, Portuguese, Italian, Dutch, German, Norwegian, and Swedish.
2. We performed keywords searching using (1) all lower case; (2) all upper case; (3) capitalize the first letter of each word; (4) capitalize the first letter of the term.

Table S5. Allocation strategies used in this study

Recipient	Project type					
	Projects primarily focused on ECCE	Projects focused on ECCE and higher-than-ECCE education	Projects focused on ECCE and non-education activities	Projects focused on childcare or child education without specifying children's age and education level	Projects reported under the CRS "Education, level unspecified" sector	Projects reported under the CRS "General budget support" sector
**Country**	No allocation	Allocated by country-year-specific proportion of ECCE-age population within the population of ECCE and the education level mentioned in the project[1-6]	No allocation	Allocated by country-year-specific proportion of ECCE-age population within the population of ECCE to tertiary education[1-6]	Allocated by country-year-specific proportion of ECCE-age population within the population of ECCE to tertiary education[1-6]	***Step 1:*** Allocated to country's education sector by country-year-specific proportion of government spending on education within the total general government spending[7]<br>***Step 2:*** Allocated to country's ECCE by country-year-specific proportion of ECCE-age population within the population of ECCE to tertiary education[1-6]
**Regional**	Allocated to each country in the region by country-year-specific proportion within region's ECCE-age population[1,2]	Allocated to each country in the region by country-year-specific proportion of ECCE-age population within region's population of ECCE and the education level mentioned in the project[1-6]	Allocated to each country in the region by country-year-specific proportion within region's ECCE-age population[1,2]	Allocated to each country in the region by country-year-specific proportion of ECCE-age population within region's population of ECCE to tertiary education[1-6]	Allocated to each country in the region by country-year-specific proportion of ECCE-age population within region's population of ECCE to tertiary education[1-6]	***Step 1:*** Allocated to each country in the region by country-year-specific proportion within total population in the region[8]<br>***Step 2:*** Allocated to country's education sector by country-year-specific proportion of government spending on education within the total general government spending[7]<br>***Step 3:*** Allocated to country's ECCE by country-year-specific proportion of ECCE-age population within the population of ECCE to tertiary education[1-6]
**Bilateral**	Allocated to each country by country-year-specific proportion within 134 LMICs' ECCE-age population[1,2]	Allocated to each country by country-year-specific proportion of ECCE-age population within 134 LMICs' population of ECCE and the education level mentioned in the project[1-6]	Allocated to each country by country-year-specific proportion within 134 LMICs' ECCE-age population[1,2]	Allocated to each country by country-year-specific proportion of ECCE-age population within 134 LMICs' population of ECCE to tertiary education[1-6]	Allocated to each country by country-year-specific proportion of ECCE-age population within 134 LMICs' population of ECCE to tertiary education[1-6]	***Step 1:*** Allocated to each country by country-year-specific proportion within total population in 134 LMICs[8]<br>***Step 2:*** Allocated to country's education sector by country-year-specific proportion of government spending on education within the total general government spending[7]<br>***Step 3:*** Allocated to country's ECCE by country-year-specific proportion of ECCE-age population within the population of ECCE to tertiary education[1-6]

Note: We imputed missing data for the related population and government spending. See **Text A1 in the Appendix** for detailed information on the imputation.
Data sources:

1. School age population, early childhood educational development programs, both sexes (UNESCO. http://data.uis.unesco.org/# [Accessed date: August 28, 2024])
2. School age population, pre-primary education, both sexes (UNESCO. http://data.uis.unesco.org/# [Accessed date: August 28, 2024])
3. School age population, primary education, both sexes (UNESCO. http://data.uis.unesco.org/# [Accessed date: August 28, 2024])
4. School age population, secondary education, both sexes (UNESCO. http://data.uis.unesco.org/# [Accessed date: August 28, 2024])
5. School age population, post-secondary non-tertiary education, both sexes (UNESCO. http://data.uis.unesco.org/# [Accessed date: August 28, 2024])
6. School age population, tertiary education, both sexes (UNESCO. http://data.uis.unesco.org/# [Accessed date: August 28, 2024])
7. Government expenditure on education, total (% of government expenditure) (World Bank. https://data.worldbank.org/indicator/SE.XPD.TOTL.GB.ZS?view=chart [Accessed date: August 28, 2024])
8. Population, total (World Bank. https://data.worldbank.org/indicator/SP.POP.TOTL?view=chart [Accessed date: August 28, 2024])

Table S6. Conflict-affected countries

**28 countries:**<br>Afghanistan, Azerbaijan, Burkina Faso, Burundi, Cameroon, Central African Republic, Chad, Colombia, Congo DR, Ethiopia, Haiti, Iraq, Libya, Mali, Mozambique, Myanmar, Nepal, Niger, Nigeria, Pakistan, Somalia, South Sudan, Sri Lanka, Sudan, Syria, Uganda, Ukraine, Yemen

Table S7. Recipient country's annual lower-bound ECCE aid per ECCE-aged child, 2007-2021 (2020USD)

Recipient country	2007	2008	2009	2010	2011	2012	2013	2014	2015	2016	2017	2018	2019	2020	2021	Average
**Low-income countries (27)**																
Afghanistan	0.02	0.03	0.02	0.10	0.37	0.01	0.04	0.10	0.21	0.08	0.07	0.11	0.14	0.18	0.75	0.15
Burkina Faso	0.07	0.14	0.10	0.25	0.39	0.09	0.45	0.37	0.36	0.28	0.21	0.49	0.83	0.37	1.00	0.36
Burundi	0.02	0.08	0.10	0.17	0.07	0.03	0.14	0.09	0.15	0.14	0.15	0.22	0.16	0.11	0.11	0.12
Central African Republic	0.03	0.02	0.02	0.02	0.04	0.12	0.17	0.03	0.02	0.23	0.05	0.16	0.91	0.06	0.11	0.13
Chad	0.04	0.07	0.05	0.02	0.13	0.00	0.01	0.01	0.02	0.04	0.05	0.09	0.06	0.05	0.05	0.05
Congo DR	0.06	0.08	0.10	0.08	0.03	0.04	0.03	0.03	0.02	0.07	0.09	0.15	0.07	0.09	0.13	0.07
Eritrea	0.20	0.04	0.04	0.35	0.17	0.09	0.04	0.01	0.02	0.11	0.05	0.14	0.10	0.04	0.08	0.10
Ethiopia	0.04	0.05	0.06	0.08	0.07	0.05	0.06	0.12	0.11	0.15	0.15	0.58	0.42	0.60	0.56	0.21
Gambia	0.23	0.15	0.10	0.05	0.05	0.30	0.01	0.03	0.02	0.08	0.54	4.88	3.91	9.77	5.41	1.70
Guinea	0.01	0.03	0.07	0.05	0.14	2.40	0.40	0.01	0.02	0.04	0.13	0.17	0.10	0.56	0.19	0.29
Guinea-Bissau	0.01	0.26	0.51	0.03	3.02	0.35	0.92	0.80	0.43	0.06	0.19	1.36	0.74	0.42	0.46	0.64
Korea DP	0.04	0.02	0.01	0.05	0.07	0.00	0.00	0.22	0.15	0.05	0.02	0.03	0.03	0.02	0.03	0.05
Liberia	0.02	0.02	0.03	0.02	0.02	0.00	0.01	0.09	0.29	21.48	0.46	6.02	9.20	6.46	0.27	2.96
Madagascar	0.02	0.10	0.03	0.04	0.01	0.07	0.07	0.08	0.07	0.08	0.09	0.34	0.28	0.35	0.54	0.14
Malawi	0.02	0.08	0.04	0.10	0.08	0.06	0.45	0.30	0.34	0.38	0.44	0.33	0.65	0.48	0.55	0.29
Mali	0.07	0.04	0.09	0.22	0.24	0.22	0.08	0.14	0.09	0.14	0.37	0.42	0.28	0.34	0.44	0.21
Mozambique	0.09	0.11	0.32	0.30	0.29	0.14	0.21	2.82	0.31	0.26	0.44	0.77	0.57	0.71	0.42	0.52
Niger	0.12	0.20	0.19	0.15	0.02	0.01	0.01	0.02	0.07	0.05	0.05	0.23	0.10	0.07	0.07	0.09
Rwanda	0.01	0.07	0.07	0.18	0.01	0.20	0.81	0.36	0.10	0.17	0.71	1.09	1.86	23.57	5.89	2.34
Sierra Leone	0.08	0.06	0.43	0.20	0.02	0.02	0.06	0.01	0.16	0.30	0.22	1.04	0.74	0.20	1.91	0.36
Somalia	0.06	0.02	0.02	0.02	0.00	0.03	0.01	0.01	0.02	0.04	0.08	0.42	0.29	0.35	0.46	0.12
South Sudan					0.00	0.00	0.01	0.01	0.02	0.08	0.05	0.11	0.16	0.22	1.48	0.14
Sudan	0.02	0.02	0.08	0.06	0.00	0.00	0.01	0.01	0.02	0.04	0.05	0.10	0.05	0.05	0.04	0.04
Syria	0.03	0.03	0.02	0.03	0.02	0.01	0.03	0.08	0.10	0.14	0.08	2.81	1.95	3.26	2.68	0.75
Togo	0.07	0.02	0.18	0.17	0.02	0.04	0.05	0.06	0.04	0.07	0.12	0.34	0.22	0.31	0.11	0.12
Uganda	0.14	0.10	0.32	0.17	0.18	0.20	0.48	0.23	0.28	0.43	0.29	0.51	0.30	0.29	1.21	0.34
Yemen	0.03	0.03	0.03	0.03	0.02	0.00	0.02	0.01	0.01	0.02	0.03	0.07	0.10	0.04	0.08	0.03
**Lower-middle-income countries (55)**																
Algeria	0.01	0.06	0.02	0.19	0.01	0.00	0.00	0.01	0.01	0.03	0.10	0.07	0.07	0.03	0.05	0.04
Angola	0.06	0.02	0.03	0.02	0.00	0.02	0.01	0.02	0.02	0.04	0.05	0.11	0.11	0.07	0.09	0.04
Bangladesh	0.07	0.27	0.20	0.29	0.30	0.31	0.29	0.22	0.07	0.04	0.05	0.30	2.41	0.42	0.59	0.39
Belize	0.42	0.02	0.52	2.81	3.27	0.02	0.01	0.01	14.17	0.03	0.06	1.58	3.38	0.22	0.14	1.78

Benin	0.01	0.09	0.07	0.07	0.04	0.03	0.02	0.08	0.11	0.11	0.51	0.37	2.18	1.65	1.26	0.44
Bhutan	0.09	0.07	0.01	0.11	0.64	1.01	1.22	0.01	0.01	0.03	0.99	2.04	11.18	1.88	3.43	1.52
Bolivia	0.28	0.09	0.32	0.44	0.33	0.96	0.64	1.99	0.37	6.47	3.12	0.38	0.44	0.29	0.37	1.10
Cabo Verde	0.01	0.02	0.02	0.02	0.00	0.00	0.47	2.42	0.54	0.26	0.25	1.83	1.00	1.86	0.67	0.63
Cambodia	0.31	0.57	0.32	0.25	0.26	0.18	0.54	0.36	1.00	0.70	0.34	0.66	0.63	0.14	0.65	0.46
Cameroon	0.04	0.04	0.12	0.11	0.02	0.03	0.02	0.02	0.03	0.05	0.07	0.24	0.58	0.27	1.32	0.20
Comoros	0.04	0.03	0.13	0.10	0.05	0.00	0.01	0.01	0.02	0.04	0.19	0.21	0.08	0.15	0.54	0.11
Congo	0.01	0.02	0.06	0.19	0.02	0.00	0.06	0.01	0.02	0.04	0.05	0.22	0.17	0.04	0.04	0.06
Côte d'Ivoire	0.02	0.06	0.12	0.18	0.03	0.01	0.06	0.04	0.07	0.08	0.82	0.66	1.20	0.90	0.14	0.29
Djibouti	0.01	0.53	0.24	0.02	1.38	1.86	1.79	0.08	1.27	0.24	0.09	0.74	0.18	1.18	3.02	0.84
Egypt	0.20	0.39	0.67	0.25	0.42	0.32	0.32	0.23	0.01	0.03	0.10	0.88	0.62	0.28	0.11	0.32
El Salvador	0.02	0.27	0.16	1.20	0.28	0.75	0.08	0.07	0.13	0.82	0.53	1.29	1.56	0.87	2.17	0.68
Eswatini	1.07	0.02	0.02	0.02	0.36	1.74	1.42	1.11	2.99	3.89	2.94	0.91	1.12	0.73	0.73	1.27
Ghana	0.56	0.21	0.08	0.13	0.03	0.10	0.05	0.09	0.13	0.16	0.14	0.39	0.81	2.64	1.20	0.45
Haiti	0.54	0.03	0.41	0.11	0.03	0.05	0.03	0.13	0.05	0.05	0.31	0.08	0.20	0.25	2.74	0.34
Honduras	2.30	0.04	0.06	0.60	0.37	1.53	1.38	0.36	0.18	0.17	0.19	0.28	0.21	0.37	0.94	0.60
India	0.01	0.03	0.01	0.02	0.00	0.00	0.00	0.01	0.01	0.04	0.08	0.07	0.08	0.05	0.09	0.03
Indonesia	0.18	0.46	0.82	1.47	0.36	0.23	0.11	0.04	0.03	0.08	0.08	0.10	0.16	0.29	0.17	0.31
Iran	0.02	0.03	0.02	0.02	0.00	0.00	0.03	0.01	0.01	0.03	0.03	0.07	0.10	0.04	0.04	0.03
Kenya	0.06	0.13	0.04	0.07	0.08	0.06	0.29	0.67	0.44	0.30	0.93	0.79	0.49	0.39	0.83	0.37
Kiribati	0.03	0.02	0.08	0.06	0.00	3.10	0.00	4.47	0.05	0.18	30.57	46.37	18.65	0.29	5.98	7.32
Kyrgyzstan	0.04	0.11	0.09	0.07	0.00	0.08	0.50	0.17	0.37	0.23	0.11	0.16	0.66	1.42	2.00	0.40
Lao	0.11	0.20	0.25	0.19	0.16	0.12	0.18	1.68	5.78	4.06	3.48	7.93	2.95	2.72	0.44	2.02
Lesotho	0.02	0.02	0.02	0.03	0.00	0.00	0.23	0.18	0.20	0.32	0.51	0.41	0.80	0.57	1.08	0.29
Mauritania	0.07	0.13	0.10	0.11	0.07	0.02	0.01	0.02	0.18	0.12	0.08	0.68	0.55	0.43	0.33	0.19
Micronesia	0.03	0.02	0.08	0.06	0.00	0.00	0.00	0.01	0.05	12.60	0.34	0.49	0.65	0.13	0.59	1.00
Mongolia	0.21	2.00	1.44	1.22	6.00	5.01	4.27	3.74	5.53	6.49	2.88	2.87	1.89	2.10	3.41	3.27
Morocco	0.73	0.20	0.56	0.29	0.17	0.12	0.17	0.10	0.19	0.22	0.14	0.41	3.93	2.28	19.68	1.95
Myanmar	0.52	0.37	0.18	0.13	0.23	0.63	0.37	0.19	0.16	0.93	0.59	0.76	0.36	0.32	0.41	0.41
Nepal	0.08	0.12	0.40	0.13	0.14	2.77	2.70	3.38	3.85	1.47	0.17	0.40	0.21	0.17	0.62	1.11
Nicaragua	1.75	0.81	1.31	1.84	7.54	0.67	0.52	0.20	0.12	0.23	1.26	0.76	1.13	3.23	2.96	1.62
Nigeria	0.02	0.05	0.05	0.06	0.03	0.01	0.02	0.02	0.02	0.04	0.05	0.12	0.08	0.07	0.14	0.05
Pakistan	0.07	0.17	0.13	0.24	0.42	0.33	0.23	0.24	0.02	0.50	0.19	0.65	0.73	0.46	0.87	0.35
Papua New Guinea	0.03	0.02	0.08	0.06	0.02	0.00	0.00	0.01	0.09	0.06	0.44	0.52	0.65	0.13	0.79	0.19
Philippines	0.01	0.02	0.03	0.21	0.03	0.26	0.03	0.36	0.03	0.06	0.51	0.06	0.06	0.04	0.06	0.12

Samoa	0.03	0.02	0.08	0.06	0.00	0.00	3.58	0.01	2.74	0.49	1.11	0.49	0.65	0.13	38.07	3.16
Senegal	0.42	0.23	0.29	0.25	0.24	0.29	0.28	0.32	0.26	0.40	0.20	0.40	1.51	1.22	1.78	0.54
Solomon Islands	0.03	0.02	0.08	0.06	1.00	0.00	0.00	3.93	3.86	0.06	1.93	1.33	1.75	0.90	2.75	1.18
Sri Lanka	0.07	0.66	0.06	0.47	0.68	0.09	0.14	0.38	0.29	1.06	2.31	3.66	3.35	4.65	1.98	1.32
São Tomé and Principe	0.01	0.48	0.02	0.18	0.09	0.48	3.40	2.09	19.75	0.31	0.49	0.42	2.28	0.91	5.28	2.41
Tajikistan	0.03	0.10	0.04	0.02	0.08	0.01	0.16	0.11	0.13	0.09	0.12	0.05	0.07	0.23	0.33	0.11
Tanzania	0.08	0.11	0.19	0.11	0.07	0.07	0.19	0.18	0.28	0.47	0.38	0.42	0.33	2.17	2.83	0.52
Timor-Leste	0.61	0.70	1.32	1.71	1.70	1.50	2.25	15.79	14.78	16.15	18.45	22.70	19.62	16.60	15.52	9.96
Tunisia	0.03	0.04	0.04	0.19	0.09	0.03	0.00	0.01	0.02	0.14	0.26	0.21	0.76	0.56	0.61	0.20
Ukraine	0.01	0.03	0.07	0.08	0.03	0.26	0.16	0.20	0.16	0.10	0.09	0.25	1.11	0.35	2.61	0.37
Uzbekistan	0.06	0.08	0.03	0.07	0.01	0.13	0.12	0.06	0.07	0.08	0.07	0.08	0.17	0.34	0.92	0.15
Vanuatu	0.03	3.83	0.08	2.01	1.84	1.38	4.04	6.32	1.42	7.91	3.38	5.82	5.95	0.13	2.29	3.09
Vietnam	0.10	0.26	0.06	0.05	0.05	0.19	2.58	3.40	3.44	1.19	1.31	0.35	0.50	0.31	0.55	0.95
West Bank and Gaza	0.90	1.67	1.99	1.94	25.59	0.92	0.59	2.46	1.55	2.39	2.97	3.30	2.34	8.48	1.85	3.93
Zambia	0.07	0.13	0.10	0.11	0.35	0.22	0.68	0.69	0.56	0.66	0.78	0.77	0.91	0.74	1.45	0.55
Zimbabwe	0.01	0.11	0.08	0.02	0.04	0.20	0.01	0.28	0.02	0.07	0.34	0.33	0.31	0.32	0.33	0.16
**Upper-middle-income countries (52)**																
Albania	2.06	0.07	0.16	0.22	0.03	0.08	1.39	0.78	0.45	0.10	0.78	4.51	3.55	5.86	8.19	1.88
Argentina	0.98	0.79	1.39	1.51	1.51	2.09	0.74	0.01	0.39	0.41	0.49	1.53	1.54	6.32	9.08	1.92
Armenia	0.07	0.11	0.31	0.02	5.64	0.00	0.31	0.06	2.28	2.98	3.65	2.79	5.73	6.59	5.47	2.40
Azerbaijan	0.01	0.01	0.09	1.34	0.09	0.44	0.51	0.21	0.10	0.37	0.13	0.32	0.30	0.13	0.31	0.29
Belarus	0.11	0.01	0.03	0.17	0.03	0.07	0.20	0.23	0.09	0.14	0.50	0.15	0.36	0.40	0.25	0.18
Bosnia and Herzegovina	0.12	1.12	0.81	0.09	2.00	0.38	1.62	0.41	0.30	0.72	0.92	3.35	2.94	2.47	3.60	1.39
Botswana	0.05	0.02	0.02	0.02	0.00	0.00	0.01	0.35	0.38	0.80	0.24	0.64	0.27	0.08	0.05	0.20
Brazil	0.02	0.05	1.40	6.19	5.86	1.20	0.57	0.87	0.71	0.97	0.90	1.17	1.51	1.09	0.16	1.51
China	0.02	0.02	0.02	0.02	0.01	0.01	0.00	0.01	0.01	0.03	0.03	0.17	0.12	0.19	0.20	0.06
Colombia	0.01	0.05	0.22	0.16	0.12	0.28	0.24	0.09	0.13	0.10	0.55	0.63	0.49	0.64	1.56	0.35
Costa Rica	0.02	0.02	0.03	0.06	0.41	1.11	1.12	0.21	0.17	0.07	0.13	0.39	0.37	0.55	0.73	0.36
Cuba	0.26	0.25	1.14	0.15	0.11	0.02	0.02	0.11	0.11	0.26	0.17	0.13	0.14	0.05	0.03	0.20
Dominica	0.02	0.02	0.03	0.04	0.02	0.02	0.01	0.01	0.01	0.03	0.06	0.05	0.04	0.04	6.34	0.45
Dominican Republic	5.26	5.43	5.67	7.11	4.44	0.14	0.13	0.14	0.17	1.29	0.85	1.02	0.54	0.35	0.35	2.19
Ecuador	0.13	0.16	0.10	0.49	0.10	0.25	0.64	0.47	0.10	1.15	1.32	3.03	1.57	0.13	0.40	0.67
Equatorial Guinea	0.01	0.02	0.30	0.02	0.00	0.00	0.01	0.01	0.02	0.04	0.05	0.09	0.05	0.04	0.04	0.05
Fiji	0.03	2.92	1.85	2.20	6.00	0.42	9.56	0.46	0.62	0.06	0.34	7.20	10.71	6.18	5.51	3.60
Gabon	0.47	0.56	0.02	0.02	0.02	0.08	0.08	0.32	0.02	0.04	0.31	0.35	0.08	0.04	0.29	0.18

Georgia	0.04	0.27	0.01	1.00	0.20	0.43	0.63	2.83	1.41	1.58	25.77	4.93	5.93	6.43	3.48	3.66
Grenada	0.02	0.02	0.03	0.04	0.02	0.02	0.01	0.01	0.01	0.03	0.06	0.05	0.04	0.04	0.03	0.03
Guatemala	0.04	0.18	0.17	0.50	0.12	0.07	0.01	0.02	0.02	0.03	0.09	0.16	0.77	0.47	1.08	0.25
Guyana	0.07	0.03	0.20	0.03	0.01	0.00	1.40	1.24	1.01	0.44	1.77	2.59	5.05	2.80	18.85	2.37
Iraq	0.24	0.02	0.11	0.04	0.08	0.00	0.04	0.01	0.03	0.06	0.03	0.42	3.88	2.00	1.44	0.56
Jamaica	0.33	0.23	0.10	0.15	0.04	0.02	0.08	13.35	10.44	0.57	1.71	0.78	1.87	0.25	0.24	2.01
Jordan	0.08	0.09	0.35	0.39	0.13	0.22	0.06	0.07	0.54	0.57	22.92	2.10	10.57	17.13	38.02	6.22
Kazakhstan	0.03	0.01	0.03	0.02	0.00	0.05	0.28	0.12	0.01	0.03	0.03	0.03	0.03	0.05	0.13	0.06
Kosovo	0.01	0.01	2.78	1.84	0.11	0.12	0.08	0.15	0.31	0.27	1.10	3.68	11.64	9.47	26.73	3.89
Lebanon	0.51	0.05	0.05	0.62	0.04	0.09	1.19	1.50	4.13	6.19	0.90	8.71	1.11	1.16	22.97	3.28
Libya	0.01	0.03	0.01	0.19	0.01	0.00	0.00	0.01	0.00	0.03	0.03	0.05	0.07	0.03	0.04	0.03
Malaysia	0.01	0.03	0.01	0.02	0.00	0.00	0.00	0.01	0.01	0.03	0.02	0.03	0.09	0.08	0.05	0.03
Maldives	0.01	0.21	0.01	0.35	0.25	0.00	0.00	0.01	0.01	0.03	0.03	0.96	0.88	0.48	0.04	0.22
Marshall Islands	0.03	0.02	0.08	0.06	0.00	0.00	8.27	0.01	0.05	0.06	0.34	0.49	51.29	0.13	27.42	5.88
Mauritius	0.01	0.02	0.02	0.02	0.00	0.00	0.01	0.01	0.02	0.25	0.38	0.09	0.05	0.04	0.04	0.06
Mexico	1.04	0.54	0.57	0.06	1.15	0.92	0.52	0.13	4.45	1.49	4.04	4.17	0.75	3.06	0.15	1.54
Moldova	0.02	0.01	0.12	0.04	0.86	1.81	2.77	18.37	3.99	2.92	2.02	6.77	2.37	1.35	0.93	2.96
Montenegro	0.65	0.01	0.14	0.05	0.14	0.06	6.13	1.56	108.43	2.09	0.67	124.25	3.20	7.52	5.22	17.34
Namibia	0.20	0.70	0.34	0.02	0.00	0.26	0.01	0.32	0.58	0.46	0.44	55.52	19.28	35.11	0.33	7.57
North Macedonia	1.06	0.02	0.38	0.57	1.60	0.53	2.97	1.21	1.99	0.43	5.56	2.04	7.02	8.14	8.67	2.81
Panama	0.12	0.12	0.17	0.22	0.25	0.02	0.03	0.01	0.06	0.59	0.50	0.69	0.92	13.66	0.65	1.20
Paraguay	0.29	0.32	0.31	0.92	0.49	0.19	0.16	0.10	0.29	0.07	0.08	0.04	0.07	3.71	0.03	0.47
Peru	0.02	0.17	0.13	0.40	0.53	0.44	0.64	2.54	3.63	3.87	1.72	0.72	0.41	0.17	14.50	1.99
Serbia	2.65	1.76	0.83	0.25	6.17	3.12	2.43	3.26	0.12	0.68	0.47	2.78	19.70	0.54	31.63	5.09
South Africa	0.05	0.07	0.05	0.04	0.00	0.01	0.05	0.04	0.06	0.12	0.13	0.55	0.86	0.22	1.75	0.27
St. Lucia	0.02	0.02	0.03	0.04	0.02	0.02	0.01	0.01	0.01	0.03	0.06	0.05	0.04	0.04	0.03	0.03
St. Vincent and the Grenadines	0.02	0.02	0.03	0.04	0.02	0.02	0.01	0.01	0.01	0.03	0.06	0.05	0.04	0.04	0.03	0.03
Suriname	0.01	0.02	0.01	0.02	0.00	0.00	0.00	0.01	0.01	0.03	0.06	0.04	0.03	0.03	0.03	0.02
Thailand	0.03	0.02	0.02	0.02	0.00	0.00	0.00	0.01	0.01	0.04	0.05	0.08	0.05	0.03	0.06	0.03
Tonga	0.03	0.02	0.08	0.06	0.00	12.44	0.00	0.01	0.05	5.30	0.56	6.42	0.65	0.13	0.59	1.76
Turkey	0.68	0.02	0.03	18.27	10.53	0.06	0.08	0.15	0.07	0.11	0.08	0.04	0.20	0.42	0.56	2.09
Turkmenistan	0.06	0.07	0.08	0.08	0.03	0.08	0.08	0.02	0.01	0.29	0.22	0.20	0.21	0.22	0.12	0.12
Tuvalu	0.03	0.02	40.34	2.08	0.00	0.00	0.00	0.01	0.05	0.06	0.34	939.21	3.73	0.13	82.09	71.21
Venezuela	0.01	0.02	0.01	0.03	0.00	0.03	0.00	0.01	0.01	0.03	0.06	0.07	0.06	0.03	0.13	0.03

Table S8. Top 10 donors for lower-bound ECCE aid with COVID-19 activities over 2020 and 2021

Donor	ECCE aid with COVID-19 activities (US$, million)	ECCE aid (US$, million)	As percentage
World Bank	46.0	496.8	9.3%
LEGO Foundation	36.4	99.0	36.8%
EU Institutions	1.6	20.4	7.7%
United Kingdom	0.7	13.6	5.1%
Conrad N. Hilton Foundation	0.5	13.4	3.7%
Spain	0.4	3.0	12.6%
Inter-American Development Bank	0.3	70.1	0.5%
Canada	0.2	13.0	1.6%
Italy	0.2	47.6	0.4%
Open Society Foundations	0.1	2.4	5.5%

Table S9. Top 10 recipient countries for lower-bound ECCE aid with COVID-19 activities over 2020 and 2021

Donor	ECCE aid with COVID-19 activities (US$, million)	ECCE aid (US$, million)	As percentage
Mexico	40.1	42.6	94.1%
Panama	6.0	6.7	89.8%
Kenya	3.9	10.3	37.9%
Iraq	3.5	23.1	15.2%
Uganda	3.5	13.8	25.1%
Bangladesh	3.4	17.6	19.4%
Nigeria	2.9	7.1	40.3%
South Sudan	2.5	3.2	78.4%
South Africa	2.4	15.9	15.1%
Burkina Faso	1.8	5.6	31.2%

Table S10. Top 10 and other donors for the upper-bound ECCE aid, 2007-2021

**Donor name**	**ECCE aid (million, 2020USD)**	**As percentage of total ECCE aid (%)**
World Bank (IBRD/IDA)	3,792.8	20.9%
EU Institutions	1,742.6	9.6%
IMF (Concessional Trust Funds)	1,429.6	7.9%
United Kingdom	1,290.4	7.1%
United States	1,089.8	6.0%
Germany	947.6	5.2%
Japan	926.6	5.1%
Inter-American Development Bank	852.5	4.7%
Canada	655.9	3.6%
United Arab Emirates	652.2	3.6%
Other donors	4,800.1	26.4%
**Sum**	**18,180.1**	**100.0%**

Table S11. Top 10 and other recipient country for the upper-bound ECCE aid, 2007-2021

**Recipient country**	**ECCE aid (million, 2020USD)**	**As percentage of total ECCE aid (%)**
Egypt	701.66	3.9%
Ethiopia	695.75	3.8%
Brazil	670.25	3.7%
Turkey	662.45	3.6%
Bangladesh	656.98	3.6%
Argentina	573.38	3.2%
India	570.70	3.1%
Tanzania	561.91	3.1%
Jordan	508.67	2.8%
Indonesia	457.20	2.5%
Other recipients	12,121.14	66.7%
**Sum**	**18,180.10**	**100%**

Table S12. Upper-bound ECCE aid per ECCE-age child by recipient country region and income group, 2007-2021

Category	Number of countries	2007	2008	2009	2010	2011	2012	2013	2014	2015	2016	2017	2018	2019	2020	2021	Annual growth rate (%)
Global	134	1.0	1.1	1.7	1.9	2.0	1.4	1.7	1.4	1.4	1.5	1.8	2.0	2.0	2.6	2.8	7.6%
Income group																	
LICs	27	2.0	2.6	3.4	2.8	3.3	2.0	2.0	1.8	1.7	1.9	2.0	3.9	3.0	4.1	3.7	4.4%
LMs	55	0.8	1.0	1.6	1.3	1.5	1.3	1.7	1.2	1.2	1.3	1.2	1.2	1.6	2.1	2.5	8.3%
UMs	52	0.9	0.7	1.0	2.6	2.4	1.4	1.3	1.4	1.4	1.6	2.9	2.3	2.4	2.6	2.9	8.7%
Region																	
East Asia and Pacific	22	0.5	0.7	0.9	1.0	0.6	0.6	1.2	0.8	0.7	0.6	0.8	0.8	0.7	1.0	1.1	6.1%
Europe and central Asia	18	3.6	1.7	1.3	8.9	6.0	1.7	1.4	2.6	1.9	2.6	7.9	4.4	4.1	8.9	9.1	6.9%
Latin America and the Caribbean	26	1.3	1.2	2.4	5.1	4.8	3.5	2.9	2.9	3.0	3.4	3.5	3.5	3.1	3.9	3.8	7.9%
Middle east and north Africa	13	1.4	2.1	3.0	1.9	2.5	2.6	4.1	2.8	3.8	4.8	4.9	6.8	5.4	4.3	9.6	14.4%
South Asia	8	0.5	0.4	0.6	0.6	0.8	0.7	1.0	0.7	0.6	0.8	0.6	0.8	1.5	0.9	0.9	3.7%
Sub-Saharan Africa	47	1.7	2.1	3.3	2.5	3.1	2.1	1.8	1.6	1.6	1.5	1.8	2.0	2.2	3.8	3.3	5.0%

Note: Annual growth rate = (ECCE aid_2021 / ECCE aid_2007) ^ (1/14) – 1

Table S13. Each recipient country's annual upper-bound ECCE aid per ECCE-aged child, 2007-2021 (2020USD)

Recipient country	2007	2008	2009	2010	2011	2012	2013	2014	2015	2016	2017	2018	2019	2020	2021	Average
**Low-income country (27)**																
Afghanistan	5.42	5.32	5.56	4.99	6.41	3.72	9.92	2.07	2.23	1.74	1.73	3.14	5.11	4.02	2.74	*4.28*
Burkina Faso	3.58	4.55	8.16	4.47	7.32	4.56	3.07	2.84	3.58	2.77	2.24	3.61	3.55	6.14	3.51	*4.26*
Burundi	2.41	3.04	2.64	3.28	2.04	1.02	1.90	2.11	1.23	0.94	1.18	1.11	1.44	1.04	3.98	*1.96*
Central African Republic	2.26	1.01	2.16	1.85	4.54	2.56	0.89	3.88	2.72	3.74	4.91	4.13	4.87	4.94	2.72	*3.14*
Chad	1.03	1.20	1.90	0.71	0.93	0.57	0.26	0.48	1.26	2.20	2.82	3.81	2.49	3.25	2.05	*1.66*
Congo DR	1.07	0.66	2.28	1.16	1.31	0.59	0.82	0.40	0.38	0.62	0.72	3.49	2.45	3.21	3.61	*1.52*
Eritrea	0.55	0.23	4.88	0.55	1.79	4.28	0.32	0.68	0.45	0.56	0.33	0.70	6.69	0.43	0.82	*1.55*
Ethiopia	1.27	2.96	5.08	3.36	5.44	3.13	1.30	1.09	0.64	0.75	0.75	1.62	1.98	2.70	1.28	*2.22*
Gambia	24.35	1.43	2.85	0.75	3.09	2.88	3.21	0.76	4.93	0.53	6.10	10.40	7.22	16.80	51.98	*9.15*
Guinea	2.43	1.98	0.38	0.29	1.02	3.64	1.18	2.02	1.73	2.58	2.96	2.64	1.04	5.39	0.73	*2.00*
Guinea-Bissau	2.30	2.84	5.71	1.67	6.78	0.52	1.33	8.12	2.87	3.59	2.36	2.51	1.87	1.95	3.57	*3.20*
Korea DP	0.10	0.04	0.04	0.09	0.14	0.07	0.25	2.40	0.36	0.35	0.27	0.29	0.28	0.25	0.25	*0.35*
Liberia	0.27	16.64	9.12	12.86	5.81	1.21	0.64	2.18	7.37	31.93	1.91	6.86	9.94	12.19	4.82	*8.25*
Madagascar	1.39	2.63	0.28	0.41	0.63	0.42	0.39	2.54	1.89	1.57	2.21	1.54	1.24	6.17	2.16	*1.70*
Malawi	2.71	4.76	1.19	4.54	1.40	3.64	1.99	1.13	1.58	2.30	1.72	1.98	2.18	3.52	1.83	*2.43*
Mali	3.98	4.29	4.80	5.52	7.36	1.07	4.83	4.82	3.33	2.71	3.33	3.21	5.77	4.08	5.26	*4.29*
Mozambique	5.01	7.05	7.71	7.92	6.70	5.48	3.90	6.76	3.87	2.09	1.97	2.04	2.67	6.14	2.63	*4.80*
Niger	1.43	1.37	1.10	0.75	2.12	2.53	1.69	3.57	2.87	1.60	1.72	1.52	3.42	2.81	3.94	*2.16*
Rwanda	11.73	6.07	5.36	9.74	10.70	2.33	2.82	1.01	1.39	3.30	2.82	2.24	3.33	28.64	8.87	*6.69*
Sierra Leone	3.22	3.50	4.19	3.25	1.92	1.88	2.38	4.44	8.54	3.31	5.50	5.04	4.31	16.48	13.14	*5.41*
Somalia	0.11	0.08	1.29	1.39	1.19	0.63	0.31	1.12	1.82	3.78	2.15	4.62	3.33	4.31	3.45	*1.97*
South Sudan					0.52	0.51	0.45	0.53	1.06	3.23	1.66	1.73	1.23	2.96	9.63	*1.57*
Sudan	0.62	0.80	2.10	1.40	1.21	0.40	0.24	0.32	0.51	0.39	1.45	1.28	3.93	1.22	8.63	*1.63*
Syria	0.25	0.28	0.10	0.31	0.49	0.15	3.40	2.03	3.15	4.03	8.66	10.75	10.74	8.42	8.55	*4.09*
Togo	0.15	3.32	3.25	4.32	1.73	0.43	1.14	0.29	1.19	1.21	5.06	5.01	1.70	6.56	0.79	*2.41*
Uganda	1.03	0.79	1.56	1.76	1.61	1.25	1.49	1.27	1.18	1.22	1.33	1.76	1.99	3.39	4.54	*1.74*

Yemen	0.20	1.52	1.99	1.89	0.78	1.26	1.53	2.16	3.58	6.30	6.55	34.12	2.25	0.69	1.50	*4.42*
**Lower-middle-income countries (55)**																
Algeria	0.16	0.12	0.19	0.31	0.22	0.22	0.24	1.04	0.49	9.75	0.38	0.44	0.36	0.28	0.29	*0.97*
Angola	0.41	0.90	0.16	0.28	0.32	0.21	2.62	1.06	1.07	0.65	0.38	0.39	0.50	0.50	0.56	*0.67*
Bangladesh	0.37	0.48	0.43	1.29	2.97	3.72	5.10	4.66	2.53	1.15	1.86	2.29	5.88	2.42	1.41	*2.44*
Belize	4.11	3.51	0.70	6.03	5.31	1.22	1.32	2.20	22.09	3.28	10.83	9.15	9.82	1.61	1.16	*5.49*
Benin	2.85	4.05	5.75	5.77	3.78	3.99	4.33	1.96	0.89	1.11	3.36	2.77	3.49	8.53	3.98	*3.77*
Bhutan	7.07	2.68	1.45	2.36	3.39	3.49	2.98	1.17	0.81	1.40	2.34	12.02	32.33	12.20	5.20	*6.06*
Bolivia	1.14	1.57	1.75	1.89	2.52	2.22	1.69	2.98	3.29	7.65	4.40	1.83	1.72	1.69	3.49	*2.66*
Cabo Verde	15.62	13.17	10.42	27.12	31.13	26.74	30.43	42.91	19.76	10.88	37.89	11.97	15.57	41.22	25.00	*23.99*
Cambodia	0.97	1.11	0.65	1.12	2.29	1.92	3.53	3.05	3.30	3.38	5.27	5.29	1.80	1.75	6.86	*2.82*
Cameroon	0.33	2.36	2.61	1.32	0.39	0.30	0.25	0.22	0.45	0.45	6.09	3.54	3.85	4.84	2.71	*1.98*
Comoros	2.03	5.57	13.63	9.41	4.46	3.46	6.91	3.08	3.22	2.59	3.02	5.95	3.64	5.60	6.40	*5.26*
Congo	0.52	0.74	0.72	2.42	3.06	0.30	0.44	0.39	0.36	0.45	0.50	0.84	15.31	0.56	0.72	*1.82*
Côte d'Ivoire	0.14	0.30	5.95	1.74	13.10	4.17	3.78	3.22	2.33	1.51	3.23	2.29	2.39	5.27	2.77	*3.48*
Djibouti	13.54	15.09	17.96	9.44	18.80	25.30	13.35	11.60	8.67	8.97	12.36	12.30	15.88	23.22	12.75	*14.62*
Egypt	1.04	2.37	3.68	0.86	0.86	0.81	9.87	1.51	5.86	4.67	0.59	2.20	2.22	1.10	12.69	*3.36*
El Salvador	0.86	2.19	2.88	3.37	2.54	3.07	1.39	1.27	1.27	1.80	1.89	2.54	2.44	2.26	4.31	*2.27*
Eswatini	1.20	0.61	0.61	0.18	1.64	2.52	1.84	1.36	3.43	18.92	18.38	4.01	3.09	2.27	1.13	*4.08*
Ghana	3.75	5.15	6.20	6.35	8.90	10.80	1.70	2.36	9.36	5.75	4.17	4.76	2.51	18.65	1.94	*6.16*
Haiti	1.56	3.22	5.74	9.17	5.35	2.93	1.18	1.52	2.34	2.56	2.36	5.33	2.03	4.43	3.97	*3.58*
Honduras	4.69	6.15	6.16	5.22	5.96	7.85	3.32	2.66	3.19	2.35	1.50	1.91	2.20	11.06	6.36	*4.71*
India	0.13	0.12	0.12	0.18	0.25	0.07	0.10	0.09	0.14	0.53	0.28	0.38	0.80	0.33	0.40	*0.26*
Indonesia	1.83	2.63	2.92	2.52	0.78	0.53	0.97	0.33	0.44	0.39	0.32	0.49	0.44	1.41	0.41	*1.09*
Iran	0.18	0.19	0.16	0.12	0.12	0.09	0.18	0.13	0.14	0.36	0.27	0.40	0.48	0.31	0.41	*0.24*
Kenya	1.61	0.57	2.29	0.52	2.91	2.09	3.00	1.34	1.47	1.07	1.90	1.41	1.14	5.67	4.05	*2.07*
Kiribati	2.99	29.53	26.48	31.46	5.88	10.79	5.79	7.30	3.75	9.21	37.42	54.46	22.77	28.91	94.86	*24.77*
Kyrgyzstan	0.76	5.50	3.56	4.87	4.39	2.75	4.93	7.99	3.65	2.79	3.51	1.13	2.11	12.52	9.28	*4.65*
Lao	0.92	3.07	2.87	11.74	4.85	8.45	4.82	2.92	6.79	6.04	5.30	10.15	7.36	7.29	4.77	*5.82*

Lesotho	0.17	0.32	2.07	7.60	4.28	8.59	5.75	0.37	0.59	4.03	11.71	2.21	1.30	3.08	1.41	*3.57*
Mauritania	2.21	0.83	0.23	2.34	3.79	2.12	3.54	1.32	7.63	4.41	2.96	3.96	3.44	8.47	7.37	*3.64*
Micronesia	4.62	5.41	0.26	3.78	3.14	3.17	1.52	2.20	2.19	17.98	7.07	14.12	9.28	6.38	8.91	*6.00*
Mongolia	2.00	3.28	1.97	18.92	12.02	14.28	11.81	6.09	7.53	8.37	33.89	18.87	11.44	29.26	8.05	*12.52*
Morocco	1.80	2.19	6.45	3.80	8.43	5.11	2.74	3.44	0.98	1.14	6.07	1.69	8.38	7.98	29.65	*5.99*
Myanmar	1.14	1.24	0.71	0.88	0.60	1.56	15.85	3.16	1.97	3.68	2.14	1.13	1.41	2.91	1.68	*2.67*
Nepal	3.62	3.75	8.39	5.86	5.18	6.80	4.55	4.63	6.34	4.52	2.09	3.40	3.08	7.95	3.42	*4.91*
Nicaragua	9.23	10.97	12.12	10.85	13.40	4.24	5.99	4.19	11.48	0.94	1.88	1.65	1.86	8.88	4.21	*6.79*
Nigeria	0.20	0.25	0.25	0.29	0.18	0.15	0.22	0.28	0.39	0.54	0.78	0.41	0.38	0.46	0.57	*0.36*
Pakistan	1.77	0.33	1.34	0.93	0.82	0.75	0.73	0.95	0.71	1.03	0.54	1.05	1.26	1.34	2.22	*1.05*
Papua New Guinea	0.39	0.41	0.41	0.80	7.61	0.74	0.77	0.52	1.09	1.55	3.99	4.38	1.66	5.44	11.93	*2.78*
Philippines	0.20	0.36	0.64	1.80	2.03	1.59	0.82	2.11	0.96	0.30	2.10	1.49	0.46	1.67	2.01	*1.24*
Samoa	9.28	10.08	29.55	106.42	46.92	26.65	21.87	6.13	11.19	13.94	9.55	10.22	4.71	76.31	121.73	*33.64*
Senegal	5.33	6.70	7.21	5.24	8.95	9.01	3.46	2.36	1.14	2.25	1.16	1.53	2.96	9.79	6.38	*4.90*
Solomon Islands	1.11	1.03	1.08	25.98	11.46	12.45	9.97	5.83	6.50	3.33	4.46	4.58	4.76	11.90	45.22	*9.98*
Sri Lanka	1.07	1.73	1.08	1.04	1.09	0.50	2.33	0.96	0.81	3.48	2.81	4.12	3.77	5.03	2.35	*2.14*
São Tomé and Principe	4.45	3.47	2.85	1.60	3.34	3.20	6.43	4.06	21.40	5.37	4.62	2.55	7.81	27.53	39.58	*9.22*
Tajikistan	0.40	0.49	1.76	2.38	1.19	2.00	1.64	1.30	5.36	3.22	2.09	1.61	2.95	11.89	3.68	*2.80*
Tanzania	4.21	4.02	7.86	4.49	2.73	3.59	4.60	2.06	1.94	1.33	1.00	0.87	0.92	3.33	6.50	*3.30*
Timor-Leste	8.79	9.57	13.10	11.03	20.59	10.02	28.19	42.19	27.78	28.82	29.88	33.56	30.68	29.95	18.83	*22.86*
Tunisia	3.85	0.39	4.06	9.42	5.54	36.26	2.73	10.23	5.00	6.33	5.67	1.83	12.33	8.51	23.65	*9.05*
Ukraine	0.43	0.41	0.38	0.57	0.52	0.99	0.78	6.42	1.75	6.20	1.20	1.25	3.05	21.92	19.56	*4.36*
Uzbekistan	0.20	0.33	0.66	2.21	1.71	0.41	0.41	0.40	0.44	0.55	0.36	0.46	0.52	2.47	3.16	*0.95*
Vanuatu	4.83	29.45	15.99	16.22	7.53	18.50	67.97	81.37	115.01	86.60	92.08	70.78	49.59	29.24	42.45	*48.51*
Vietnam	0.85	1.50	4.33	2.09	1.44	2.00	4.70	5.19	4.24	1.72	2.22	1.10	1.48	1.02	1.09	*2.33*
West Bank and Gaza	9.31	19.90	29.36	21.69	46.82	21.87	13.17	10.16	6.12	7.58	40.37	40.28	34.58	23.81	20.58	*23.04*
Zambia	1.80	3.01	12.95	7.66	3.59	3.74	2.63	5.23	1.79	2.26	2.21	1.34	3.23	2.56	6.48	*4.03*
Zimbabwe	0.16	0.30	0.40	0.34	0.33	0.56	0.34	1.12	1.26	0.81	0.83	0.72	0.74	0.61	0.68	*0.61*
**Upper-middle-income countries (52)**																

Albania	3.58	2.82	2.25	1.54	4.32	7.54	5.22	6.58	1.95	1.61	2.86	6.40	20.38	23.33	73.58	*10.93*
Argentina	1.09	1.29	1.87	19.46	16.52	15.72	15.90	12.88	8.23	7.08	8.00	2.28	3.63	7.07	9.39	*8.69*
Armenia	3.91	3.28	3.60	9.34	24.07	8.18	7.21	2.63	2.78	5.74	5.19	4.71	52.71	10.45	12.17	*10.40*
Azerbaijan	0.09	0.95	0.32	2.40	5.41	0.69	0.85	0.50	0.42	0.61	66.03	2.34	0.80	2.31	0.66	*5.62*
Belarus	0.14	0.37	0.21	0.61	0.62	0.45	0.70	0.65	0.81	1.17	1.66	1.10	1.72	31.85	0.72	*2.85*
Bosnia and Herzegovina	0.81	4.04	2.94	1.85	3.76	3.25	3.97	4.10	1.74	2.15	3.22	5.64	28.34	5.35	31.95	*6.87*
Botswana	0.32	0.45	0.97	31.18	125.24	1.46	15.99	33.83	7.34	2.21	15.03	1.06	1.43	1.27	6.19	*16.27*
Brazil	0.65	0.31	1.60	6.59	6.17	3.44	1.94	2.51	1.40	2.73	2.84	2.34	2.89	2.00	0.85	*2.55*
China	0.06	0.08	0.09	0.11	0.10	0.10	0.10	0.11	0.14	0.22	0.24	0.41	0.40	0.42	0.52	*0.21*
Colombia	0.18	0.47	1.66	2.29	3.59	2.18	2.68	0.91	0.65	4.15	1.10	1.26	1.28	2.28	4.37	*1.94*
Costa Rica	0.34	1.41	1.38	1.62	1.95	2.77	2.59	1.57	1.25	1.31	1.77	2.09	2.39	2.95	3.12	*1.90*
Cuba	0.41	0.53	1.75	0.41	0.43	0.61	0.61	0.32	0.34	0.59	0.76	0.66	1.20	0.30	1.03	*0.67*
Dominica	0.05	21.96	16.85	38.79	30.42	12.79	24.88	9.32	33.08	33.13	16.66	31.67	101.37	65.96	6.98	*29.60*
Dominican Republic	6.25	6.33	8.41	9.47	5.43	4.32	1.61	1.70	3.11	1.84	1.46	1.64	0.96	3.60	0.92	*3.80*
Ecuador	0.34	1.14	6.34	1.77	1.07	1.10	2.43	1.38	0.70	13.57	8.92	11.47	10.13	1.29	4.28	*4.40*
Equatorial Guinea	5.14	0.51	6.93	14.62	2.00	1.31	0.66	0.67	0.37	0.56	0.43	0.49	16.41	0.39	0.75	*3.42*
Fiji	2.44	7.62	62.01	22.22	8.52	3.88	10.65	1.34	3.00	4.61	11.57	8.99	14.57	9.09	90.42	*17.40*
Gabon	0.77	0.66	0.15	0.37	4.49	3.86	1.97	1.89	0.91	0.40	98.13	13.85	25.09	13.35	0.81	*11.11*
Georgia	5.28	19.15	3.10	4.87	3.41	15.50	13.71	26.93	14.87	2.81	28.78	8.66	13.96	43.22	15.15	*14.63*
Grenada	0.05	32.70	52.68	39.46	5.73	0.11	1.02	38.70	13.31	14.84	9.67	2.32	37.01	61.95	0.36	*20.66*
Guatemala	0.44	1.57	5.08	1.60	0.60	5.43	5.18	6.10	1.22	5.56	3.00	5.74	6.52	4.94	2.81	*3.72*
Guyana	0.66	4.03	3.36	0.77	0.69	2.00	1.86	2.36	1.23	0.93	2.58	3.12	5.78	3.17	22.55	*3.67*
Iraq	3.33	0.57	1.73	0.77	0.60	0.15	0.51	1.96	3.28	3.89	4.53	5.35	7.01	3.64	2.91	*2.68*
Jamaica	1.52	9.41	11.11	49.43	8.27	1.07	5.55	18.21	16.26	2.02	3.71	2.32	7.66	0.90	0.99	*9.23*
Jordan	8.35	20.57	14.18	16.20	18.85	21.43	14.50	27.85	21.22	10.61	44.98	22.80	42.99	46.04	54.68	*25.68*
Kazakhstan	0.21	0.23	0.44	0.44	0.30	0.43	0.53	0.28	0.42	1.69	0.49	0.50	0.58	0.61	0.36	*0.50*
Kosovo	0.03	0.06	9.57	12.98	3.89	5.54	2.39	6.80	12.88	5.51	7.03	7.81	14.38	25.94	43.33	*10.54*
Lebanon	3.05	11.50	7.57	3.20	2.03	3.68	6.60	18.34	18.56	33.18	52.34	29.13	41.97	56.11	48.14	*22.36*
Libya	0.08	0.13	0.14	0.39	0.68	1.44	0.14	1.01	1.15	0.63	2.92	2.40	1.92	1.06	0.94	*1.00*

Malaysia	0.19	0.25	0.14	0.27	0.36	0.37	0.33	0.29	0.27	0.38	0.38	0.48	0.53	0.45	0.39	*0.34*
Maldives	8.74	5.21	1.80	6.55	4.21	3.25	2.37	2.59	3.75	3.84	5.15	2.34	3.45	47.96	0.60	*6.79*
Marshall Islands	6.56	5.91	0.42	17.03	21.60	12.44	15.32	5.10	6.27	6.90	14.69	3.33	54.31	1.91	61.34	*15.54*
Mauritius	10.61	43.49	70.08	69.85	39.99	26.62	79.62	78.70	13.58	14.14	0.67	0.41	0.38	0.36	110.33	*37.25*
Mexico	2.04	0.77	1.10	0.45	2.59	1.28	0.86	0.40	4.75	1.82	4.45	4.59	1.19	3.47	0.62	*2.02*
Moldova	26.05	20.66	11.20	57.54	35.22	28.39	3.72	23.19	4.72	7.44	6.45	10.73	7.62	50.38	29.16	*21.50*
Montenegro	0.88	3.86	4.04	3.77	2.97	4.13	11.62	6.46	113.68	3.47	2.34	125.44	4.52	41.73	38.85	*24.52*
Namibia	0.65	4.30	6.52	3.73	11.81	14.90	14.76	3.13	10.94	2.43	3.29	58.32	22.17	36.57	22.77	*14.42*
North Macedonia	2.50	19.43	13.54	2.09	4.11	5.34	7.49	7.53	5.53	3.42	8.24	5.38	10.33	48.56	42.26	*12.38*
Panama	6.38	2.96	3.21	1.21	0.85	0.70	8.47	16.18	0.82	1.34	1.05	4.44	1.38	18.85	1.14	*4.60*
Paraguay	1.33	1.67	2.62	4.61	1.65	1.05	0.95	1.51	1.00	2.33	6.76	6.37	14.39	4.48	9.14	*3.99*
Peru	1.03	1.36	1.49	1.39	1.52	1.04	1.49	3.24	4.58	4.75	2.89	6.34	8.44	11.31	24.23	*5.01*
Serbia	3.29	5.40	6.23	5.40	13.54	6.91	9.19	15.24	5.48	14.37	77.73	24.50	37.39	3.85	37.77	*17.75*
South Africa	0.19	0.63	0.67	0.59	0.72	0.95	1.37	0.64	1.35	1.49	1.20	1.36	2.03	1.14	2.54	*1.12*
St. Lucia	0.88	1.90	21.75	2.05	25.74	29.08	56.48	38.28	5.57	9.29	15.09	11.69	7.38	95.21	21.03	*22.76*
St. Vincent and the Grenadines	1.80	1.34	25.75	1.61	30.14	37.38	54.15	42.02	39.21	3.43	2.55	8.21	6.49	103.20	103.10	*30.69*
Suriname	60.19	0.11	26.13	4.26	0.73	0.12	0.28	0.35	12.86	0.20	0.44	40.62	27.92	0.70	16.73	*12.78*
Thailand	0.22	0.16	0.13	0.32	0.40	0.53	3.21	0.21	0.28	0.42	0.40	0.51	0.54	0.61	0.42	*0.56*
Tonga	25.11	27.31	2.07	17.23	32.03	34.55	126.69	7.69	65.41	61.25	35.56	27.98	10.20	17.28	61.36	*36.78*
Turkey	7.88	1.24	0.89	19.29	11.61	0.98	0.78	0.96	0.96	2.45	9.10	8.55	4.29	5.96	9.54	*5.63*
Turkmenistan	0.23	0.24	0.27	0.13	0.08	0.14	0.17	0.13	0.13	0.55	0.42	0.60	0.49	0.46	0.40	*0.30*
Tuvalu	9.19	97.92	94.05	90.16	60.20	355.31	259.56	237.79	494.07	71.89	327.95	1071.53	257.40	152.71	312.74	*259.50*
Venezuela	0.06	0.23	0.40	0.41	0.33	0.41	0.35	0.32	0.50	0.34	0.47	0.53	0.99	0.94	1.60	*0.53*

Figure S1. Flow chart of project search and review process

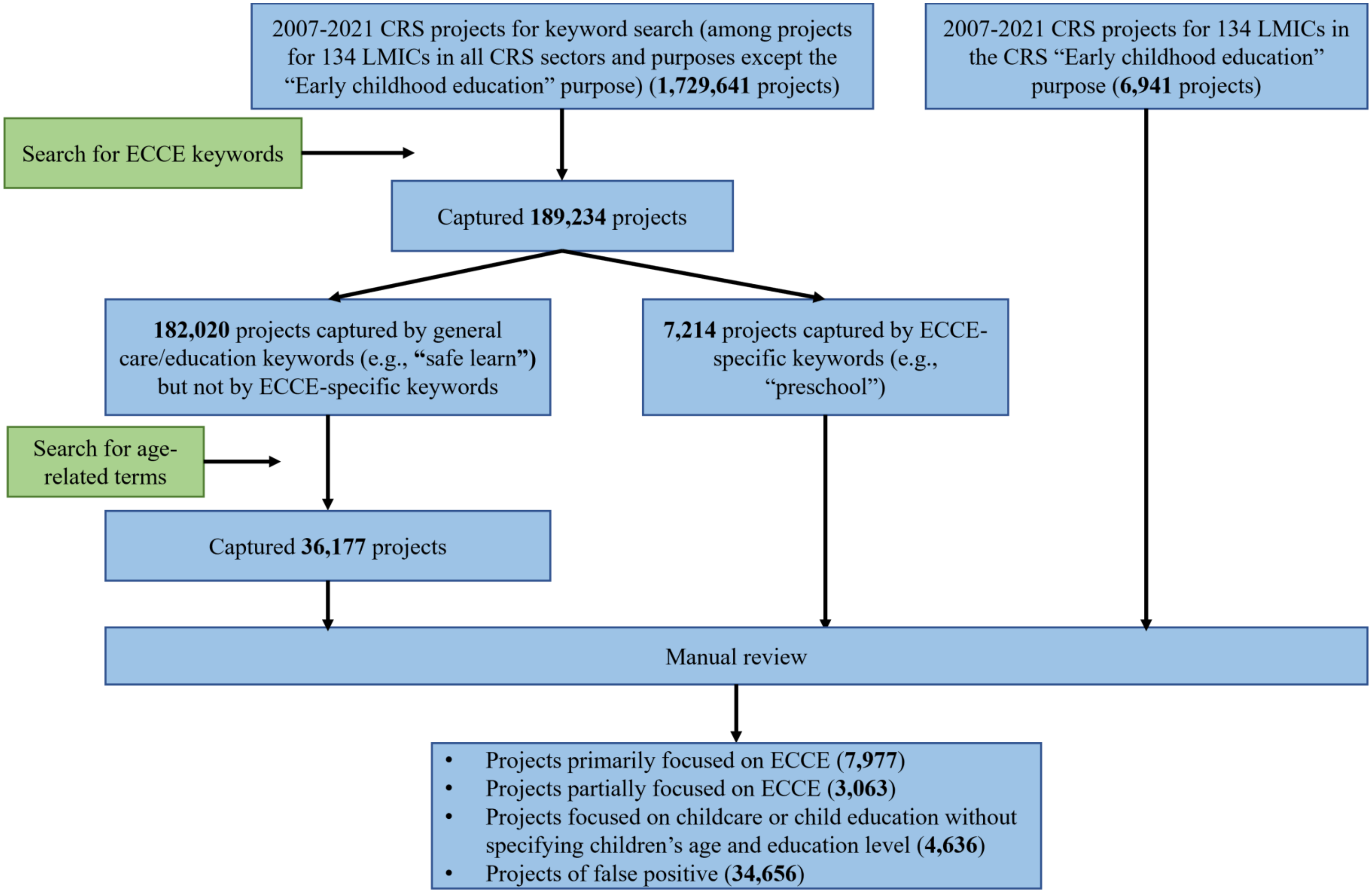

Figure S2. Lower-bound ECCE aid levels and trends by recipient countries' income groups, 2007-2021

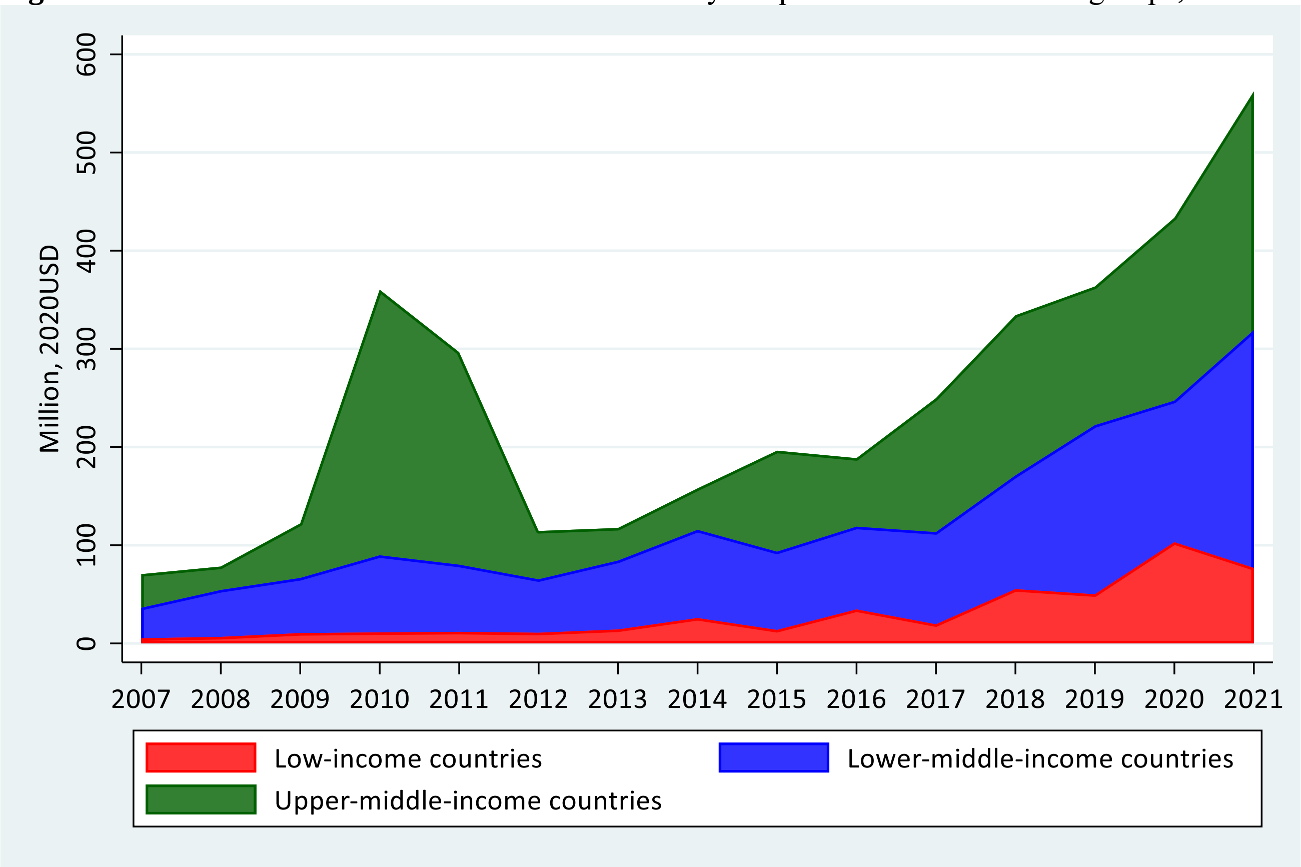

Figure S3. Lower-bound ECCE aid levels and trends by recipient countries' region, 2007-2021

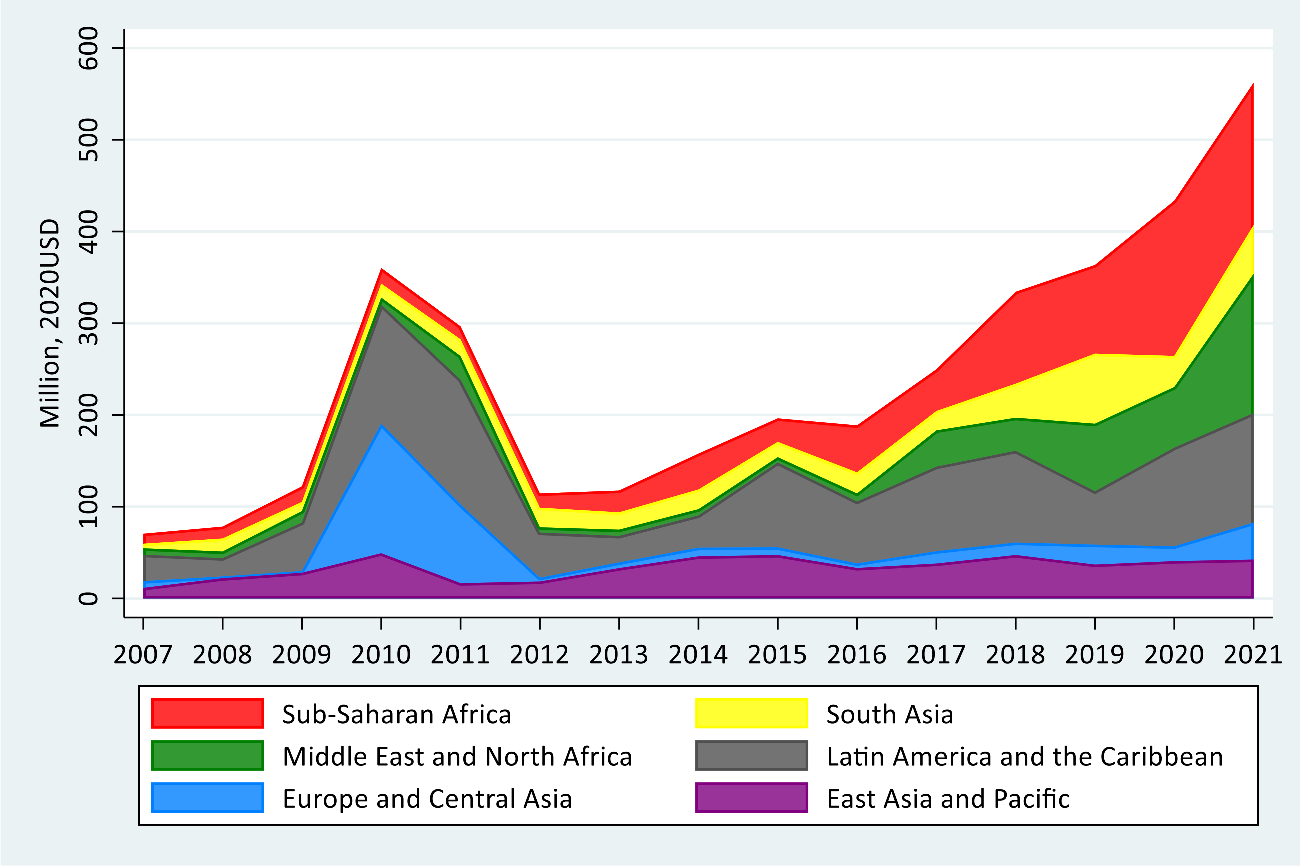

Figure S4. Lower-bound ECCE aid levels and trends by CRS sector, 2007-2021

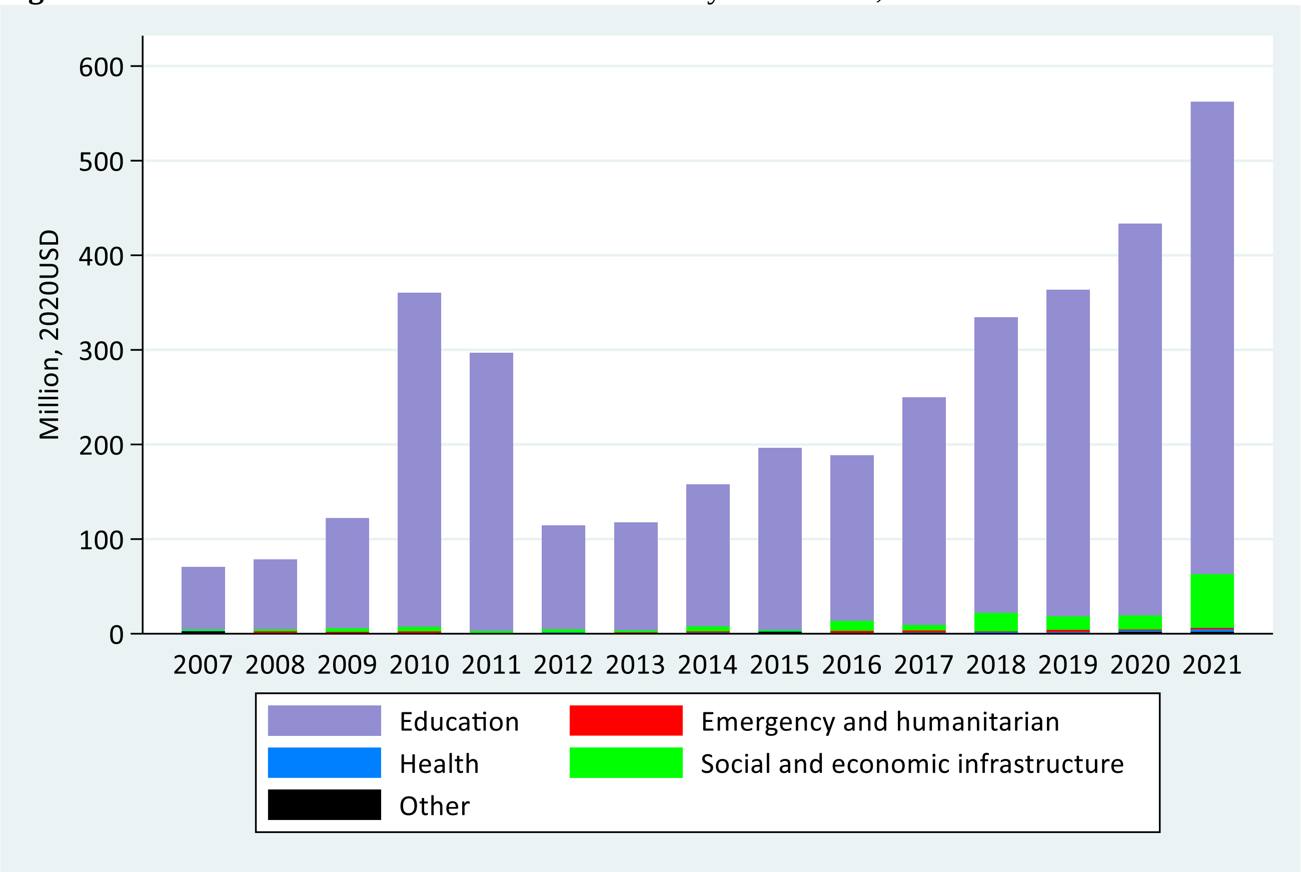

Note: In this figure, we combined the sectors "Basic education", "Secondary education" "Post-secondary education", "Education, level unspecified" as "Education"; combined "Emergency response", "Conflict, peace & security", "Refugees in donor countries", "Reconstruction relief & rehabilitation", and "Disaster prevention & preparedness" as "Emergency and humanitarian"; combined "Health, general", "Basic health", "Non-communicable disease (NCDs)", and "Population policies/programmes & reproductive health" as "Health"; combined "Government & civil society-general", "Other social infrastructure & services", "Water supply & sanitation", "Energy policy", "Energy generation, renewable sources", and "Banking & financial services" as "Social and economic infrastructure".

Figure S5. Lower-bound ECCE aid levels and trends by channel, 2007-2021

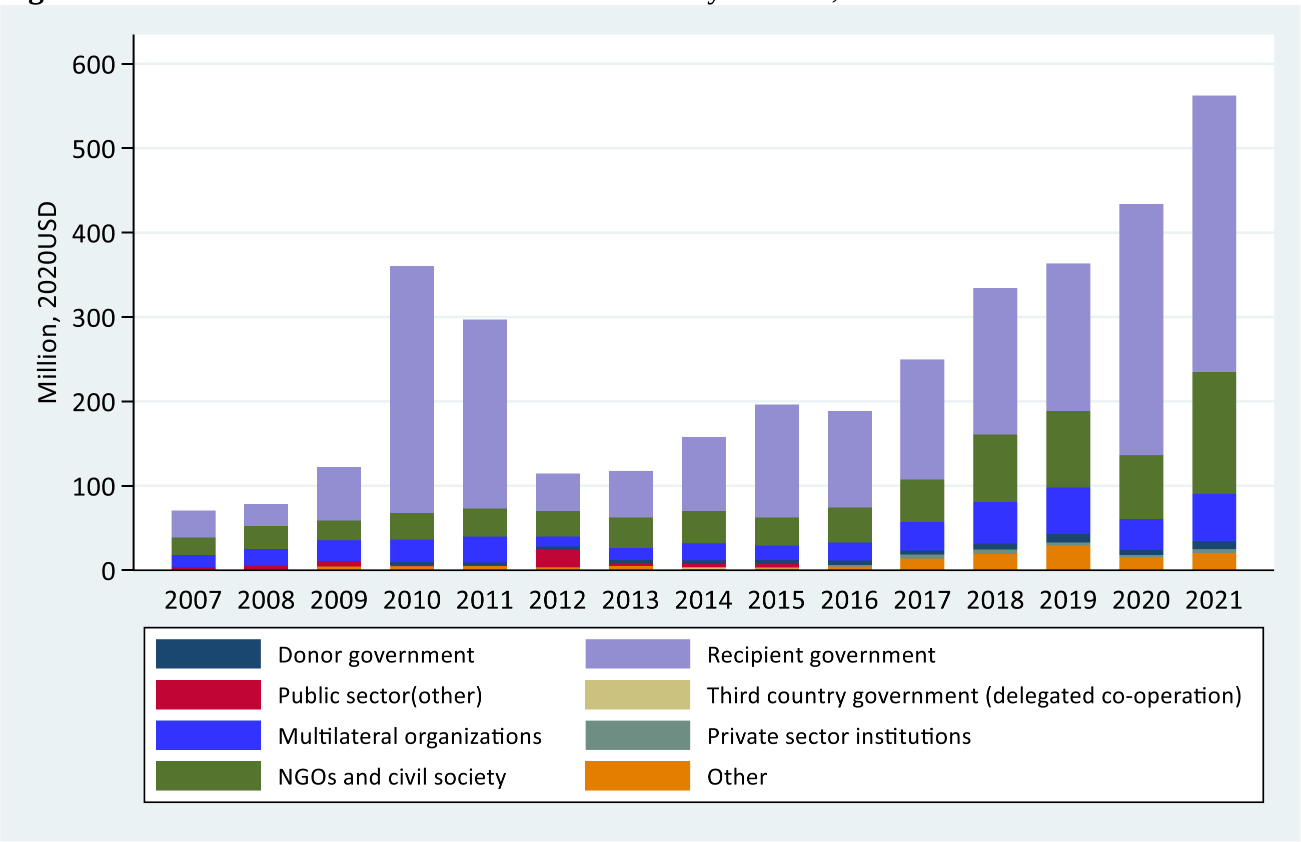

Figure S6. Lower-bound ECCE aid levels and trends by flow type, 2007-2021

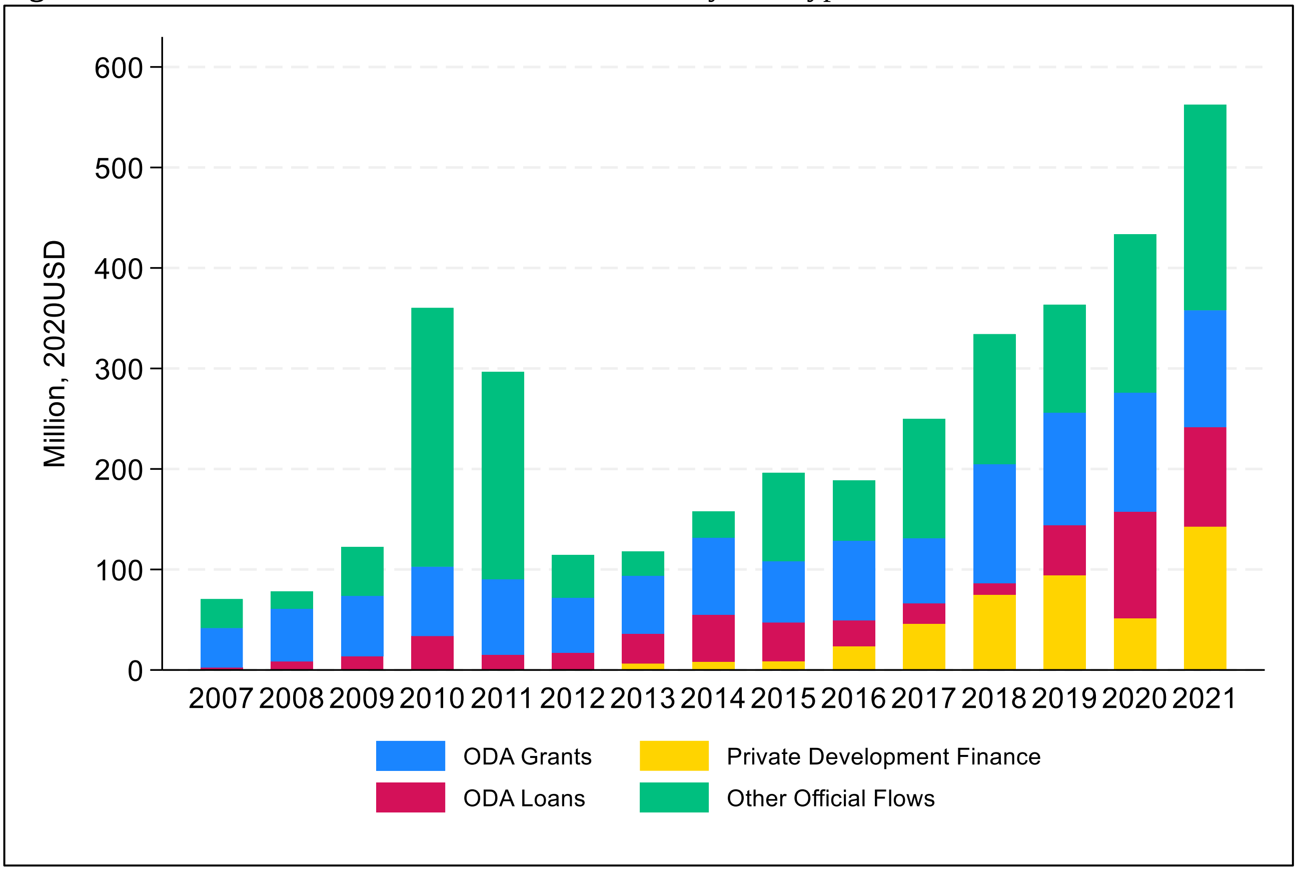

Figure S7. Donor's lower-bound ECCE aid as percentage of DAE disbursed during the SDG study years (2016-2021)

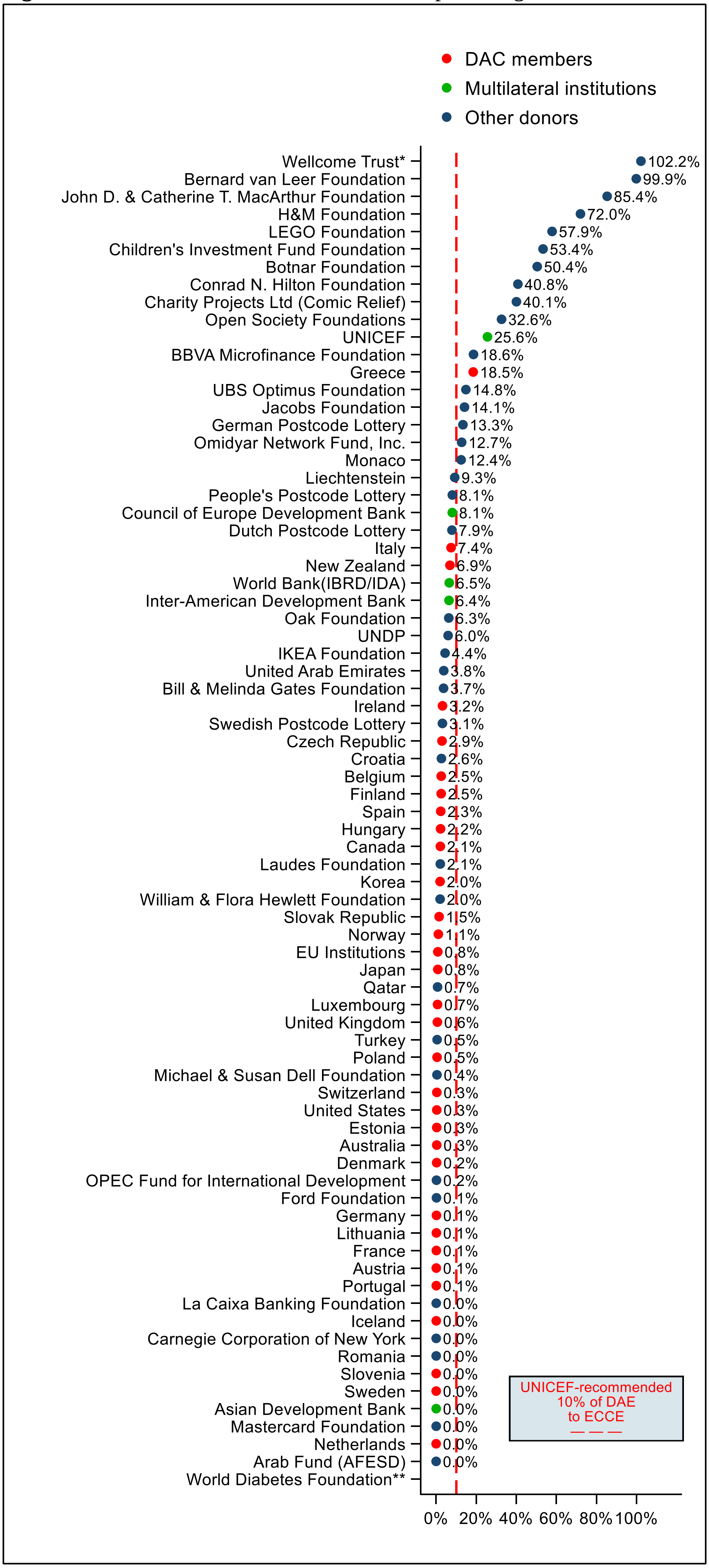

Note:

1. Percentages are calculated by dividing each donor's total ECCE aid disbursed during the SDG study years (2016-2021) by the donor's total project disbursements reported under the four CRS education sectors during the same years. The four CRS education sectors include "basic education", "secondary education", "post-secondary education", and "education, level unspecified".

2. The percentage may exceed 100% when donors reported ECCE aid projects under sectors other than the four CRS education sectors, and the aid value for these ECCE projects was larger than the total aid value under the four education sectors (e.g., Wellcome Trust*).

3. The percentage may be missing when donors reported ECCE aid projects under other than the four CRS education sectors but did not report any aid projects under the four education sectors. (e.g., World Diabetes Foundation**).

Figure S8. Lower-middle-income countries' match between ranks in lower-bound ECCE aid per child and enrolment rate

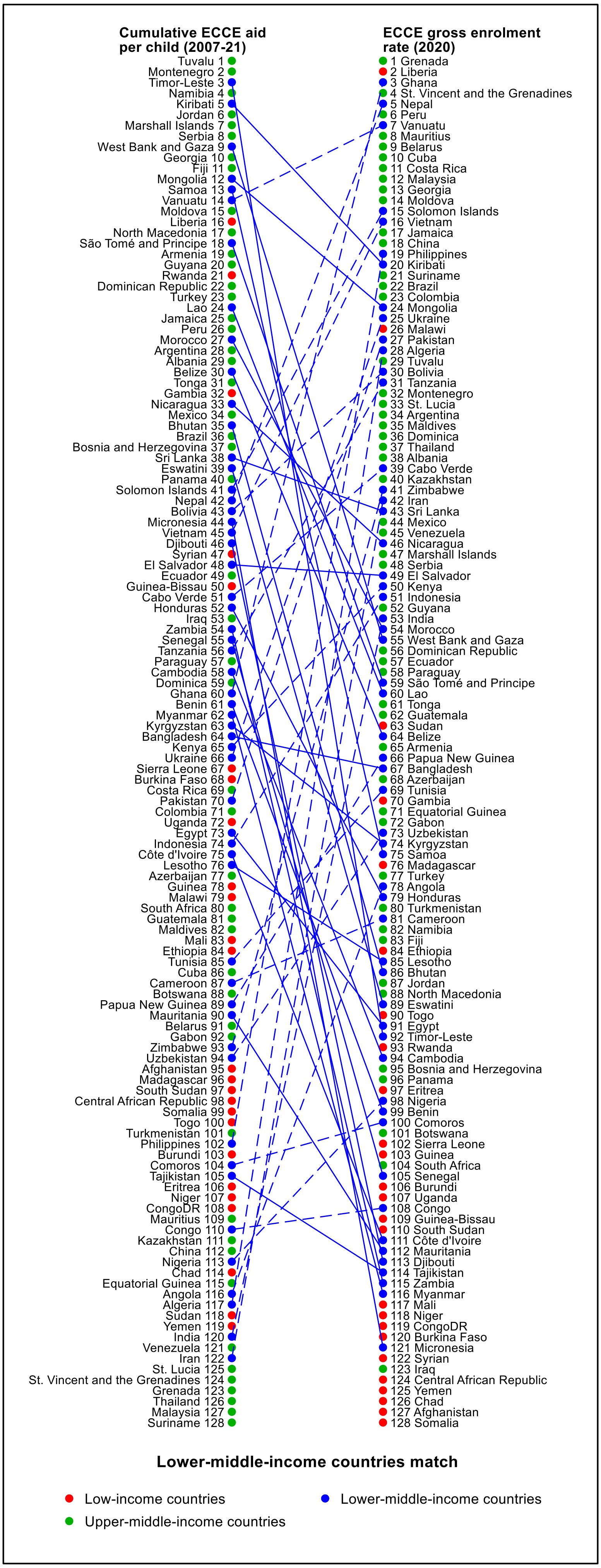
Cumulative ECCE aid per child (2007-21)
Tuvalu 1
Montenegro 2
Timor-Leste 3
Namibia 4
Kiribati 5
Jordan 6
Marshall Islands 7
Serbia 8
West Bank and Gaza 9
Georgia 10
Fiji 11
Mongolia 12
Samoa 13
Vanuatu 14
Moldova 15
Liberia 16
North Macedonia 17
São Tomé and Principe 18
Armenia 19
Guyana 20
Rwanda 21
Dominican Republic 22
Turkey 23
Lao 24
Jamaica 25
Peru 26
Morocco 27
Argentina 28
Albania 29
Belize 30
Tonga 31
Gambia 32
Nicaragua 33
Mexico 34
Bhutan 35
Brazil 36
Bosnia and Herzegovina 37
Sri Lanka 38
Eswatini 39
Panama 40
Solomon Islands 41
Nepal 42
Bolivia 43
Micronesia 44
Vietnam 45
Djibouti 46
Syrian 47
El Salvador 48
Ecuador 49
Guinea-Bissau 50
Cabo Verde 51
Honduras 52
Iraq 53
Zambia 54
Senegal 55
Tanzania 56
Paraguay 57
Cambodia 58
Dominica 59
Ghana 60
Benin 61
Myanmar 62
Kyrgyzstan 63
Bangladesh 64
Kenya 65
Ukraine 66
Sierra Leone 67
Burkina Faso 68
Costa Rica 69
Pakistan 70
Colombia 71
Uganda 72
Egypt 73
Indonesia 74
Côte d'Ivoire 75
Lesotho 76
Azerbaijan 77
Guinea 78
Malawi 79
South Africa 80
Guatemala 81
Maldives 82
Mali 83
Ethiopia 84
Tunisia 85
Cuba 86
Cameroon 87
Botswana 88
Papua New Guinea 89
Mauritania 90
Belarus 91
Gabon 92
Zimbabwe 93
Uzbekistan 94
Afghanistan 95
Madagascar 96
South Sudan 97
Central African Republic 98
Somalia 99
Togo 100
Turkmenistan 101
Philippines 102
Burundi 103
Comoros 104
Tajikistan 105
Eritrea 106
Niger 107
CongoDR 108
Mauritius 109
Congo 110
Kazakhstan 111
China 112
Nigeria 113
Chad 114
Equatorial Guinea 115
Angola 116
Algeria 117
Sudan 118
Yemen 119
India 120
Venezuela 121
Iran 122
St. Lucia 125
St. Vincent and the Grenadines 124
Grenada 123
Thailand 126
Malaysia 127
Suriname 128
ECCE gross enrolment rate (2020)
1 Grenada
2 Liberia
3 Ghana
4 St. Vincent and the Grenadines
5 Nepal
6 Peru
7 Vanuatu
8 Mauritius
9 Belarus
10 Cuba
11 Costa Rica
12 Malaysia
13 Georgia
14 Moldova
15 Solomon Islands
16 Vietnam
17 Jamaica
18 China
19 Philippines
20 Kiribati
21 Suriname
22 Brazil
23 Colombia
24 Mongolia
25 Ukraine
26 Malawi
27 Pakistan
28 Algeria
29 Tuvalu
30 Bolivia
31 Tanzania
32 Montenegro
33 St. Lucia
34 Argentina
35 Maldives
36 Dominica
37 Thailand
38 Albania
39 Cabo Verde
40 Kazakhstan
41 Zimbabwe
42 Iran
43 Sri Lanka
44 Mexico
45 Venezuela
46 Nicaragua
47 Marshall Islands
48 Serbia
49 El Salvador
50 Kenya
51 Indonesia
52 Guyana
53 India
54 Morocco
55 West Bank and Gaza
56 Dominican Republic
57 Ecuador
58 Paraguay
59 São Tomé and Principe
60 Lao
61 Tonga
62 Guatemala
63 Sudan
64 Belize
65 Armenia
66 Papua New Guinea
67 Bangladesh
68 Azerbaijan
69 Tunisia
70 Gambia
71 Equatorial Guinea
72 Gabon
73 Uzbekistan
74 Kyrgyzstan
75 Samoa
76 Madagascar
77 Turkey
78 Angola
79 Honduras
80 Turkmenistan
81 Cameroon
82 Namibia
83 Fiji
84 Ethiopia
85 Lesotho
86 Bhutan
87 Jordan
88 North Macedonia
89 Eswatini
90 Togo
91 Egypt
92 Timor-Leste
93 Rwanda
94 Cambodia
95 Bosnia and Herzegovina
96 Panama
97 Eritrea
98 Nigeria
99 Benin
100 Comoros
101 Botswana
102 Sierra Leone
103 Guinea
104 South Africa
105 Senegal
106 Burundi
107 Uganda
108 Congo
109 Guinea-Bissau
110 South Sudan
111 Côte d'Ivoire
112 Mauritania
113 Djibouti
114 Tajikistan
115 Zambia
116 Myanmar
117 Mali
118 Niger
119 CongoDR
120 Burkina Faso
121 Micronesia
122 Syrian
123 Iraq
124 Central African Republic
125 Yemen
126 Chad
127 Afghanistan
128 Somalia
Lower-middle-income countries match
Low-income countries
Lower-middle-income countries
Upper-middle-income countries

Figure S9. Upper-middle-income countries' match between ranks in lower-bound ECCE aid per child and enrolment rate

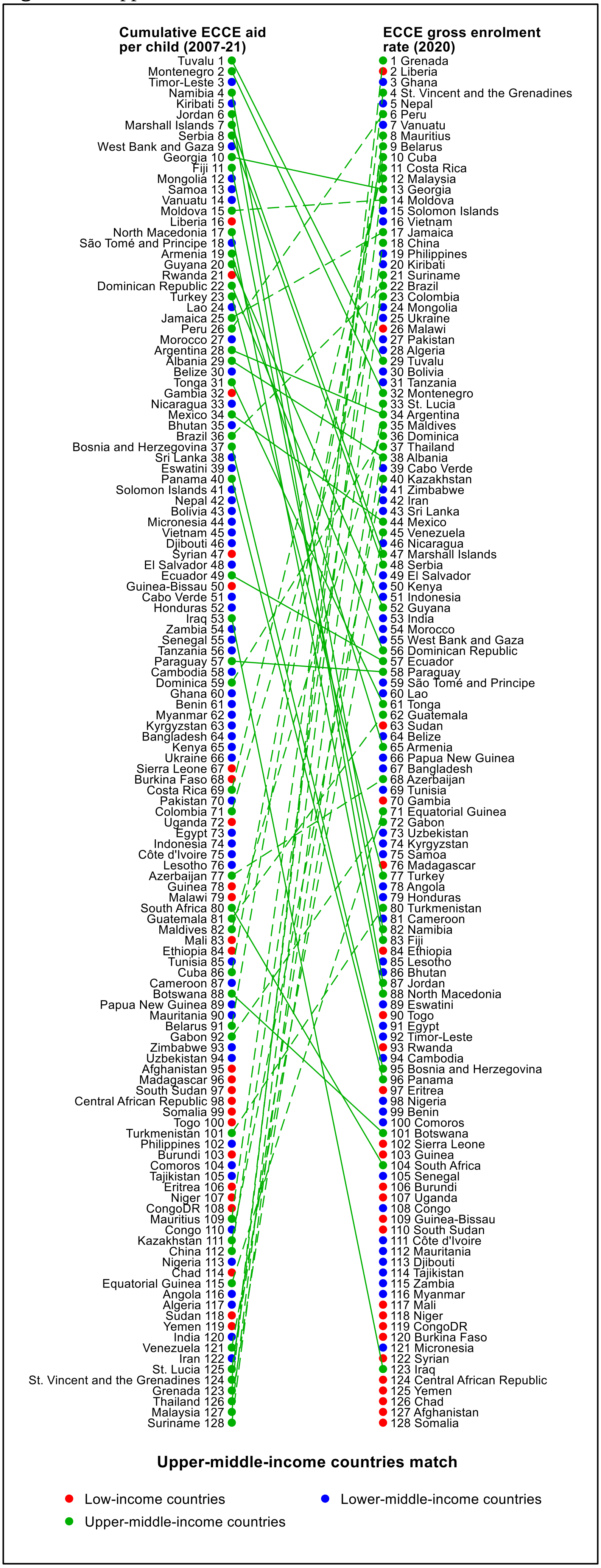

Figure S10. Average lower-bound ECCE aid per ECCE-age child among conflict-affected countries versus non-conflict-affected countries

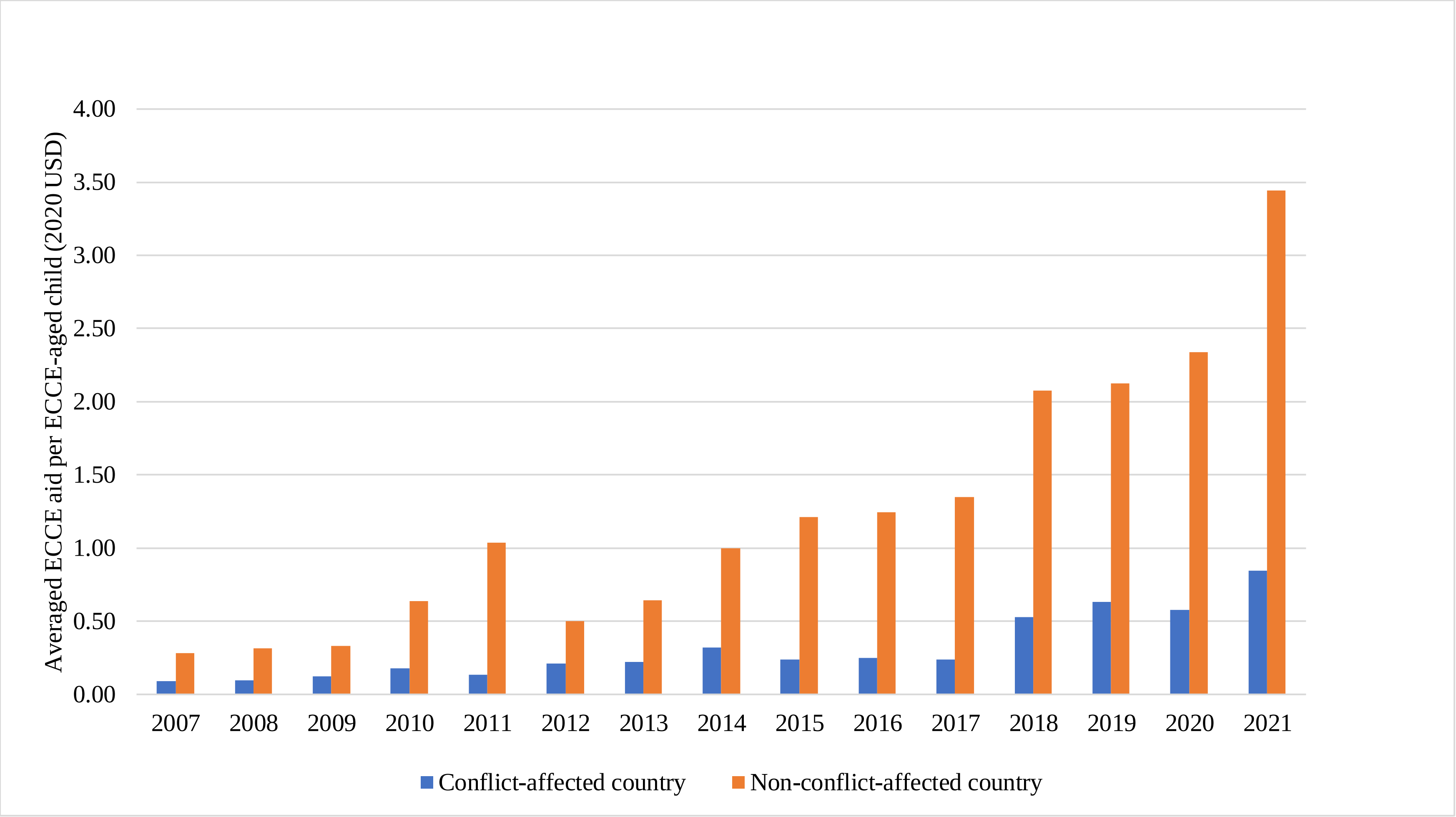

Figure S11. Upper-bound ECCE aid and as percentage of DAE, 2007-2021

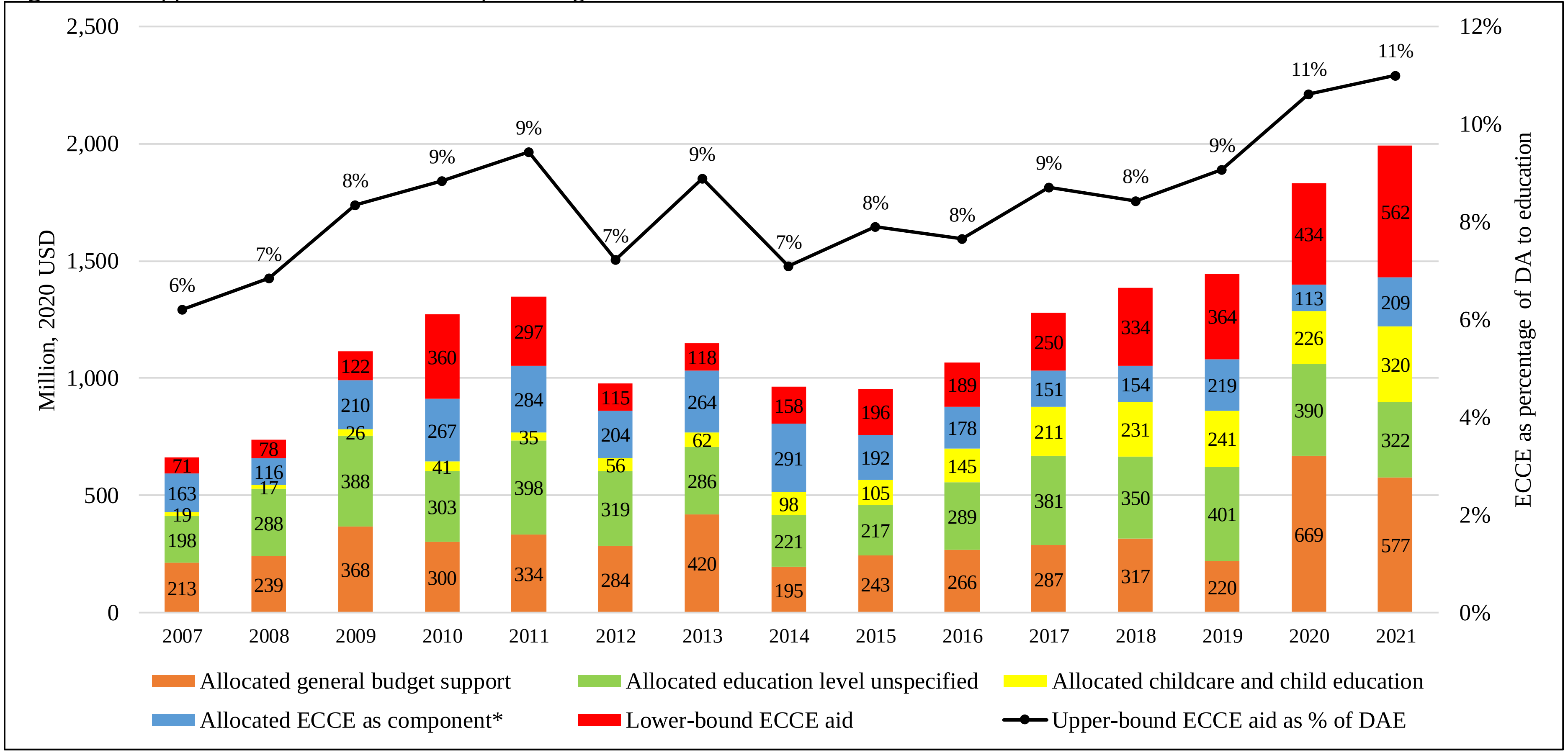

*. Allocation ECCE as component = allocated disbursements of the projects integrating ECCE and higher-than-ECCE educational activities at the country, regional, and bilateral levels + allocated disbursements of the projects integrating ECCE and non-educational activities at the regional and bilateral levels.

Figure S12. Annual upper-bound ECCE aid by World Bank income group, 2007-2021

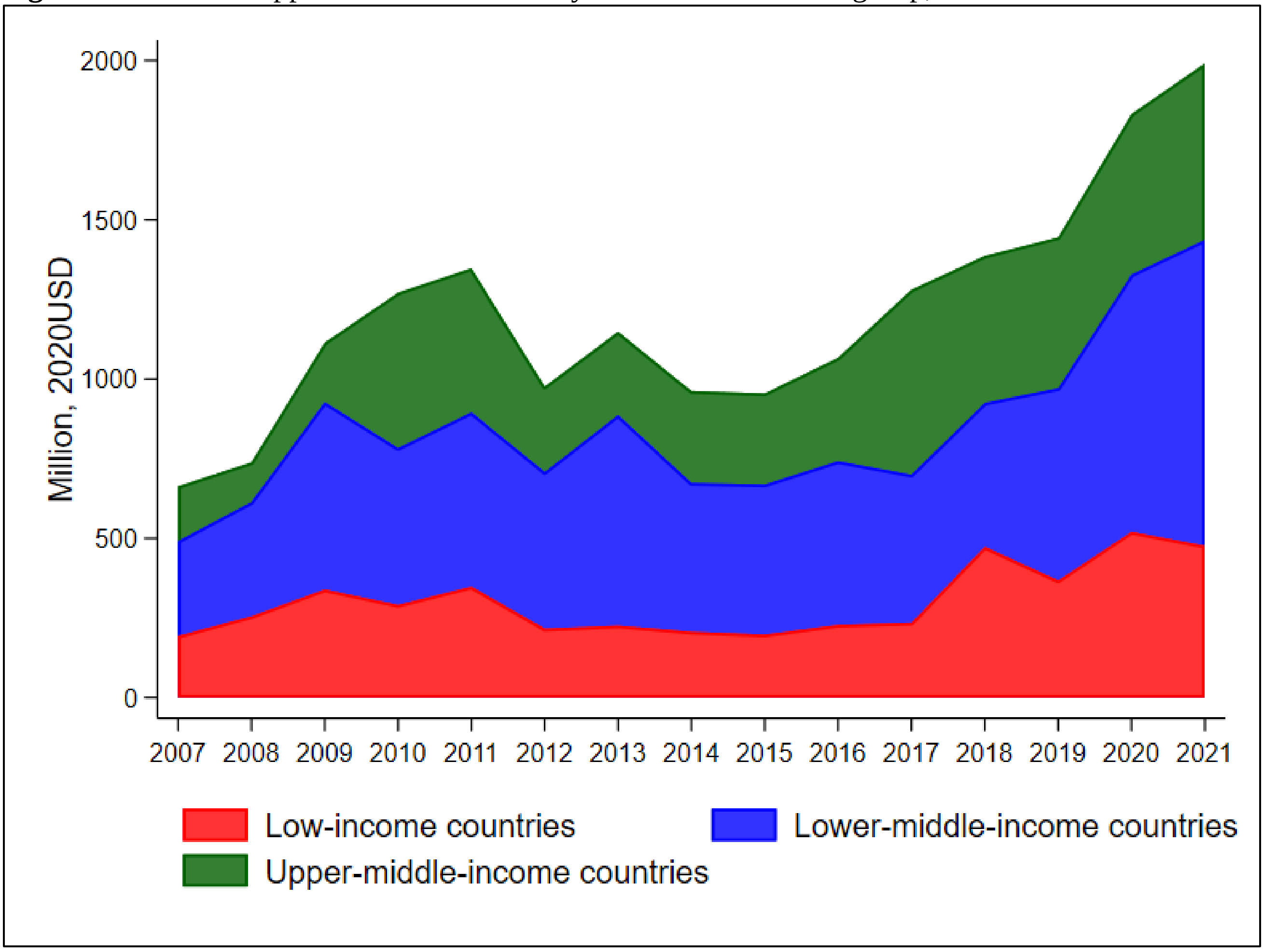

Figure S13. Annual upper-bound ECCE aid by World Bank region, 2007-2021

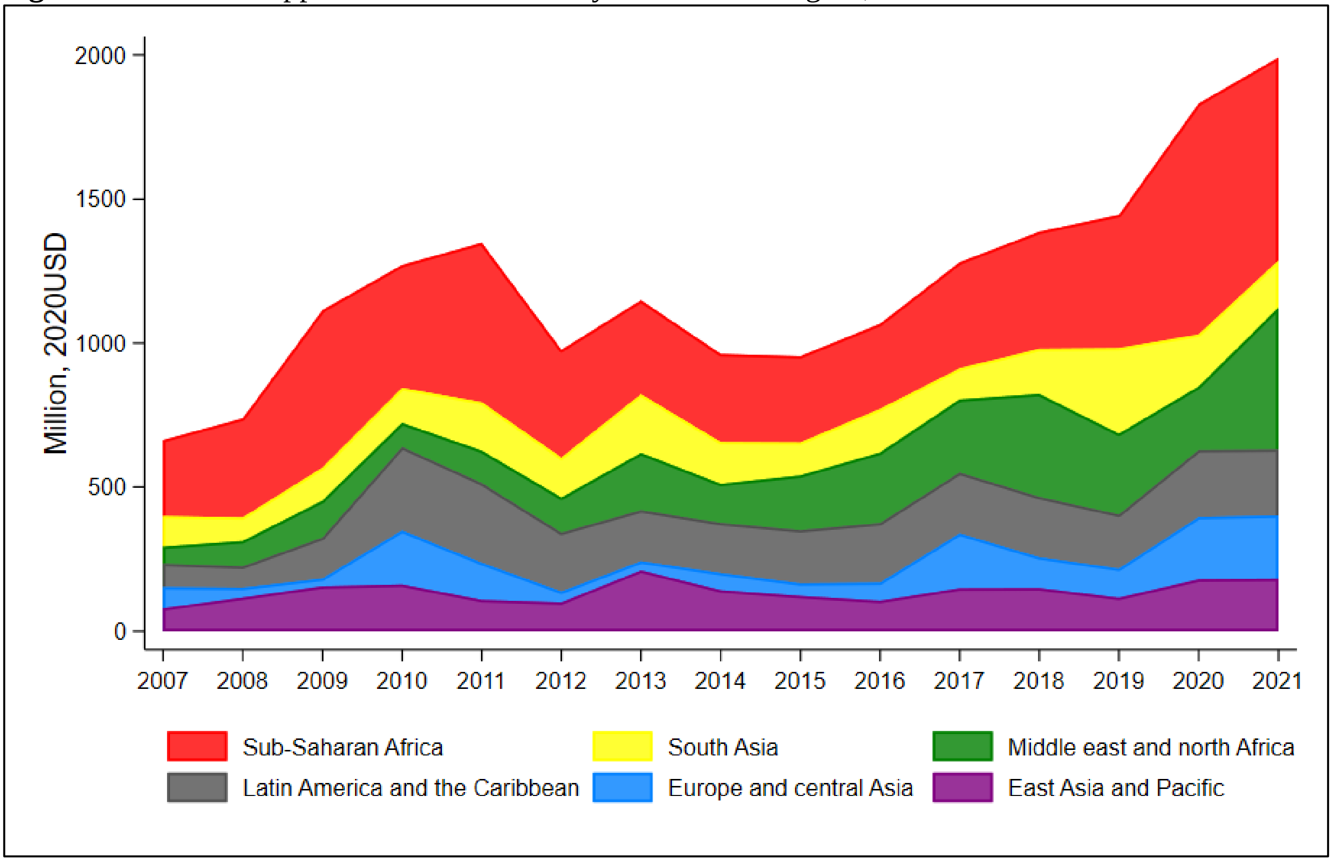

Figure S14. Upper-bound ECCE aid by sector, 2007-2021

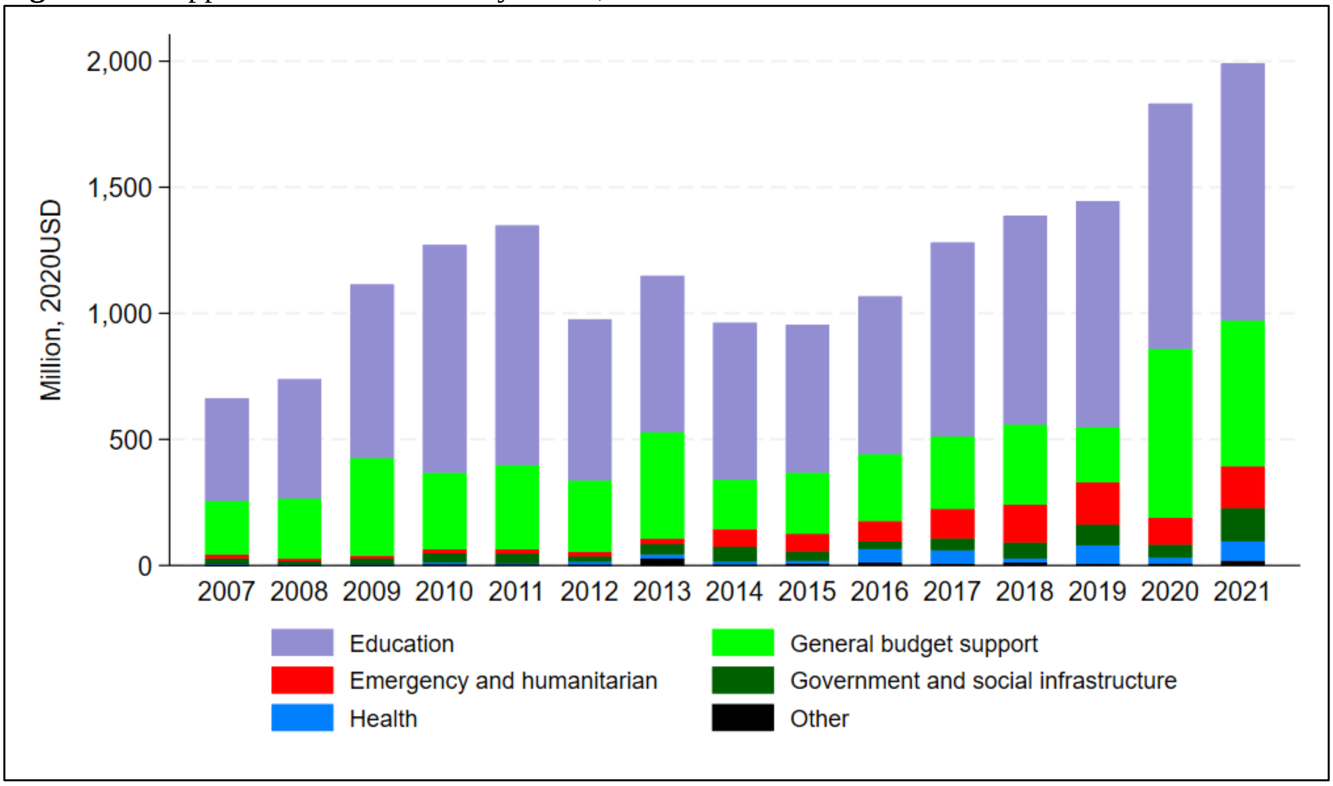

Note: In this figure, we combined the sectors "Basic education", "Secondary education" "Post-secondary education", "Education, level unspecified" as "Education"; combined "Emergency response", "Conflict, peace & security", "Refugees in donor countries", "Reconstruction relief & rehabilitation", and "Disaster prevention & preparedness" as "Emergency and humanitarian"; combined "Health, general", "Basic health", "Non-communicable disease (NCDs)", and "Population policies/programmes & reproductive health" as "Health"; combined "Government & civil society-general", "Other social infrastructure & services", "Water supply & sanitation", "Energy policy", "Energy generation, renewable sources", and "Banking & financial services" as "Social and economic infrastructure".

Figure S15. Upper-bound ECCE aid by channel, 2007-2021

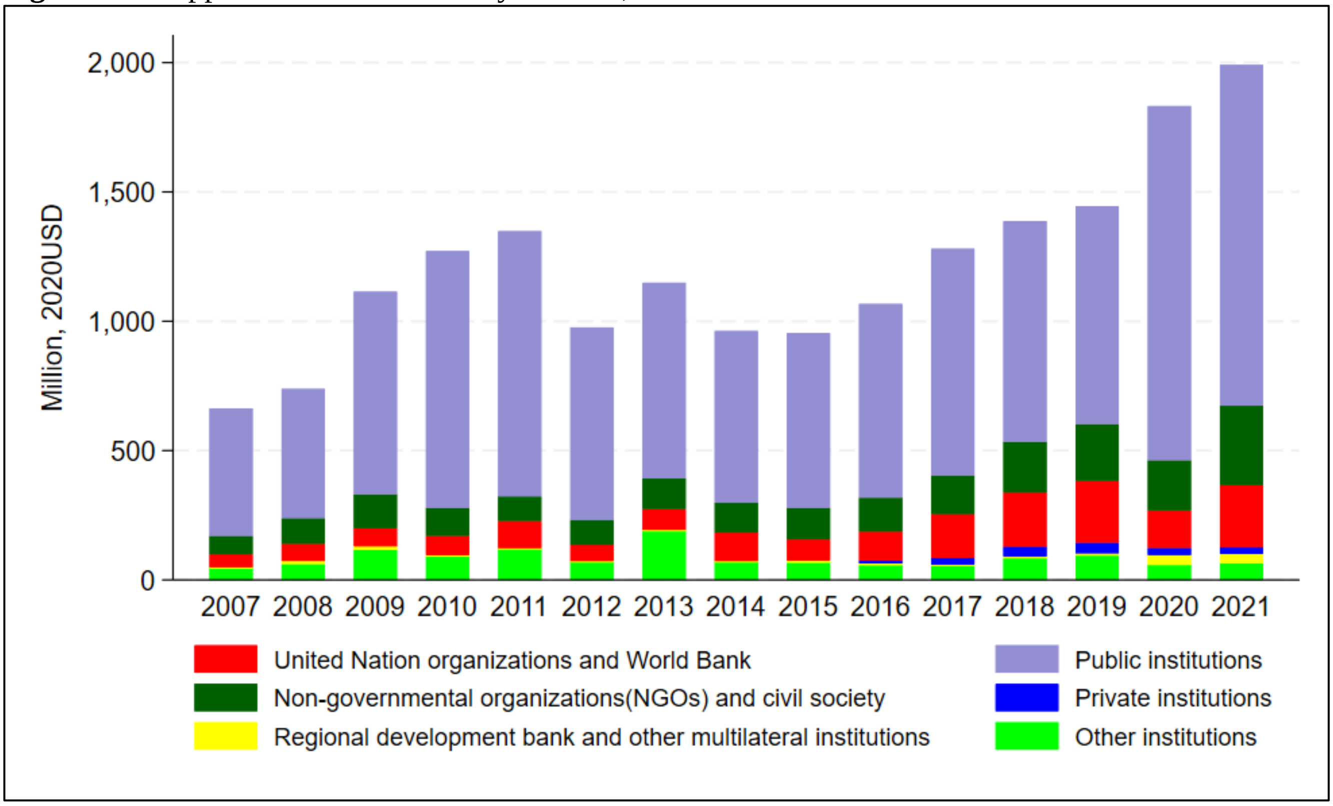

Figure S16. Top 10 and other donors of upper-bound ECCE aid, 2007-2021

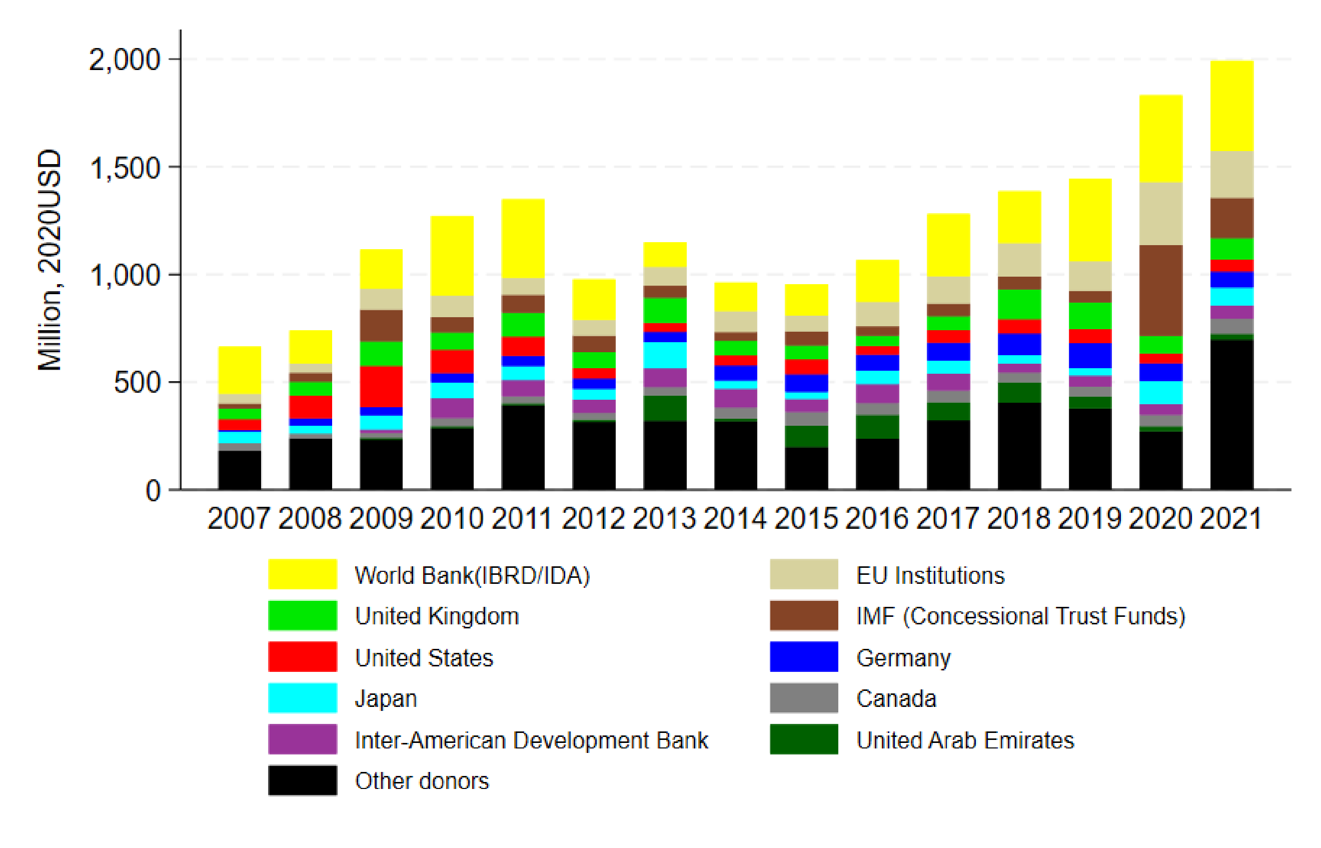

Figure S17. Top 10 and other recipient countries of upper-bound ECCE aid, 2007-2021

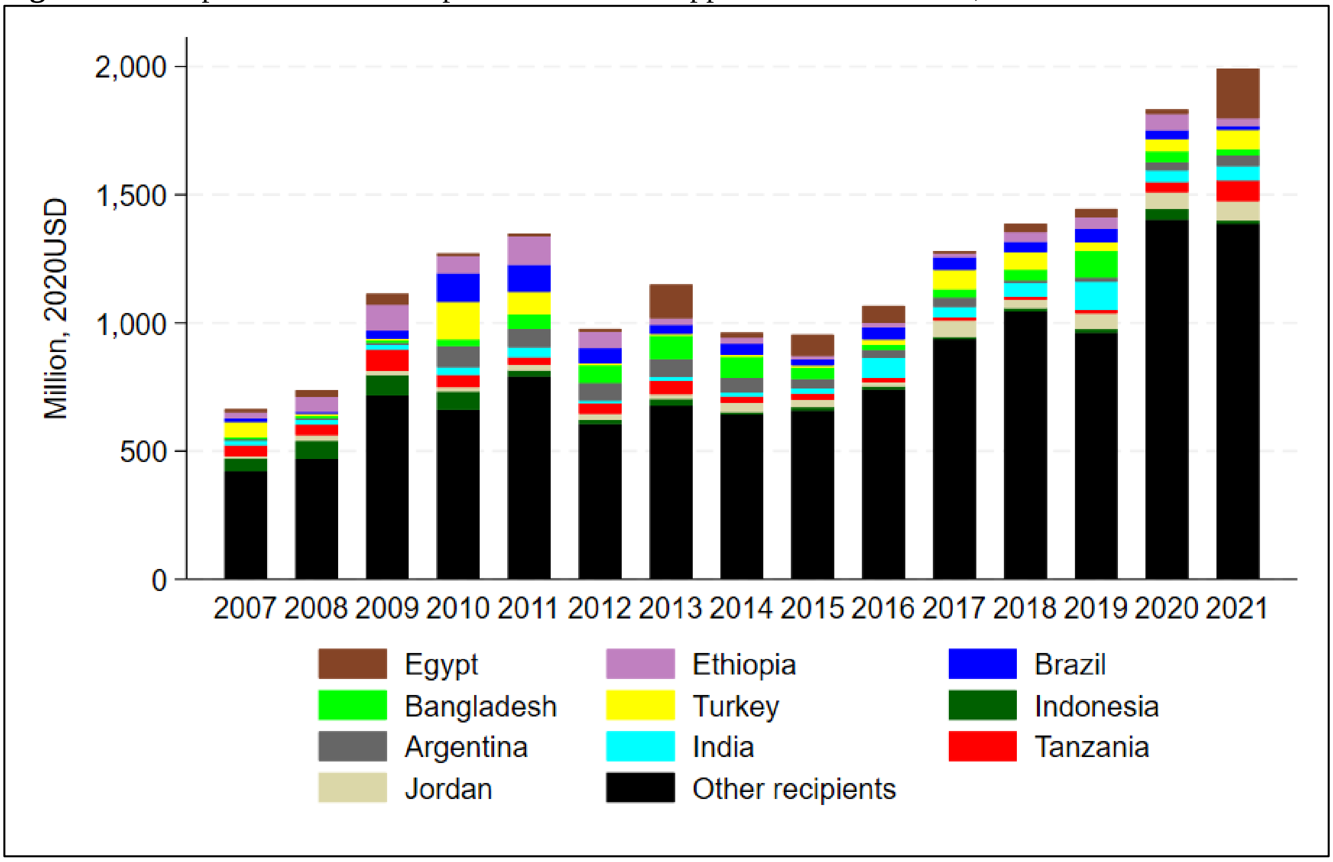

Figure S18. Average upper-bound ECCE aid per ECCE-age child among conflict-affected country versus non-conflict-affected country

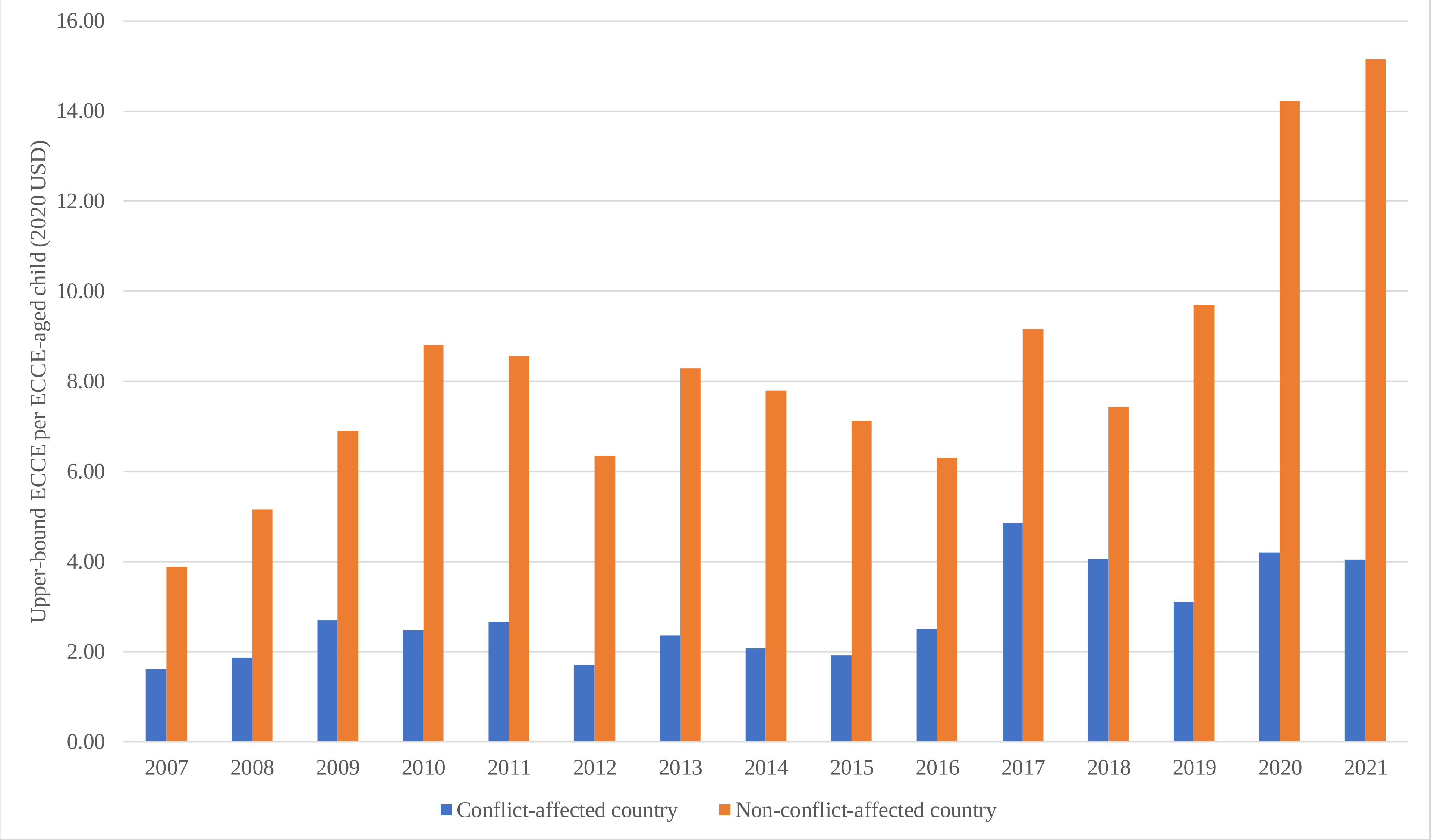

Text S1. Imputing missing data

1. *Imputing missing data for ECCE-age population*

We derived country-level ECCE-age population by summing up each country's number of eligible population for attending early childhood educational development program (ECED) and pre-primary education (PPE). Both population data were provided by UNESCO based on each country's regulations or laws on official entrance age and duration for ECED and PPE programs (see data sources in **Table S5 in Supplement**). Twenty-two countries have available population data for both ECED and PPE for each of the years between 2007 and 2021, while the rest of the 112 countries have missing population data within the study period (15 years). As the 112 countries are heterogeneous in data availability, we divided them into five groups and developed each group a strategy to impute their ECCE-age populations.

(1) Seventy-six countries do not have any population data for ECED but have available PPE population data for the 15 years

We imputed each country's ECED population data by evenly dividing each country's under-five population by five and replaced each country's missing with the country's population from age zero to its country-year-specific official PPE entrance age, with the assumption that all young children before the official PPE entrance age should be eligible for ECED programs. We obtained country-level annual data on under-five population from the United Nation Population Division (UNPD)[1] and country-year-specific PPE entrance age from UNESCO[2].

(2) Two countries have no population data for both ECED and PPE

Kosovo and Lebanon have neither population data for ECED and PPE nor data for PPE entrance age within the 15 years. We replaced their ECCE-age population with each country's under-five population derived from UNPD[1].

(3) Twenty-five countries have ECED population data for less than 15 years but have available PPE population data for 15 years

All these twenty-five countries have at least two years with available ECED population data, enabling us to follow previous studies in using linear interpolation or extrapolation to impute missing values[3]. We graphed the trend of each country's ECED population with available data points and visually examined data's linearity as it has been suggested as the condition under which the linear interpolation or extrapolation method would impute missing values with the most appropriateness[4]. The graph showed that most of the countries in most years followed approximately linear trends for their available ECED population data and the trends followed the patterns of most countries' under-five population well, validating our usage of the interpolation or extrapolation methods for imputing the missing data on ECED populations.

(4) Six countries have both ECED and PPE population data for less than 15 years

Of the six countries, Jamaica has only one available data point for its PPE population (in year 2007), where we imputed Jamaica's missing PPE population data by applying the same year-by-year growth rate of its under-five population. We imputed Jamaica's ECED and other five countries' ECED and PPE populations with the linear interpolation or extrapolation methods mentioned above, as these countries all have at least two available data points within the 15 years. After the imputation, we noticed that Bosnia and Herzegovina's imputed ECED population turned

to be negative owing to the country's steep upward slope in its available data, and thus we changed the imputation method for Bosnia and Herzegovina by applying the same year-by-year growth rate of its under-five population.

(5) Three countries have no ECED population data but have PPE population data for less than 15 years

Such countries include Maldives, Solomon Islands, and Vanuatu. Following practices in *(1)*, we imputed each country's ECED population data by evenly dividing each country's under-five population by five and replaced each country's missing with the country's population from age zero to its year-specific official PPE entrance age. We used linear interpolation and extrapolation to impute each country's missing PPE population as each country has at least two available PPE population data points within the 15 years.

Reference:
1. UNPD. Total population (both sexes combined) by five-year age group. https://population.un.org/wpp/Download/Standard/Population/ (Accessed date: August 28, 2024).
2. UNESCO. Official entrance age to primary education. http://data.uis.unesco.org/# (Accessed date: August 28, 2024)
3. Lu, C., Black, M. M., & Richter, L. M. (2016). Risk of poor development in young children in low-income and middle-income countries: an estimation and analysis at the global, regional, and country level. The Lancet Global Health, 4(12), e916-e922.
4. Armstrong, J. S. (1984). Forecasting by extrapolation: Conclusions from 25 years of research. Interfaces, 14(6), 52-66.

2. *Imputing missing population data for higher-than-ECCE educational levels*

The purpose of imputing missing population data for countries' higher-than-ECCE educational levels is to facilitate the fund allocation listed in **Table S5 in Supplement**.

(1) Primary education

Of the 134 LMICs, Bosnia and Herzegovina, Lebanon, and Kosovo do not have any years with available population data for primary education. We imputed missing values in the following ways: For Bosnia and Herzegovina, its entrance age for primary education remained at 6-years old while the education duration changed from four years in 2007-2009 (then attendees were of age 6-9) to five years in 2010-2021 (then attendees were of age 6-10)[1,2]. Thus, we imputed Bosnia and Herzegovina's missing population for primary education by 4*(5 to 9 aged population/5) for 2007-2009 and 4*(5 to 9 aged population/5) + (10 to 14 aged population/5) for 2010-2021. For Lebanon, its entrance age for primary education remained at 6-years old and education duration remained six years (then attendees were of age 6-12) from 2007 to 2021. Thus, we imputed Lebanon's population by 4*(5 to 9 aged population/5) + 3*(10 to 14 aged population/5). Kosovo does not have any data for entrance age and duration for its primary education, where we assumed that it starts at 6-years old and lasts for six years (then attendees were of age 6-11). Therefore, we imputed Kosovo's missing population by 4*(5 to 9 aged population/5) + 2*(10 to 14 aged population/5).

Besides, 10 countries have available population data for at least two years but less than 15 years for primary education, for which we visually examined their linearities for available data and used linear interpolation or extrapolation for imputations. Additionally, one country, Jamaica, has only one available data for its primary education population (for year 2007), where we imputed Jamaica's missing by applying the same year-by-year growth rate of its 5-9 aged population as

Jamaica's official entrance age for primary education is at year six.

(2) Secondary education

Of the 134 LMICs, Bosnia and Herzegovina, Lebanon, and Kosovo do not have any years with available population data for secondary education. We imputed their missing values in the following ways: For Bosnia and Herzegovina, its entrance age for secondary education changed from 10-years old for 2007-2009 to 11-years old for 2010-2021 while its education duration remained eight years (then attendees were of age 10-17 for 2007-2009 and age 11-18 for 2010-2021)[3,4]. Thus, we imputed Bosnia and Herzegovina's population by (10 to 14 aged population) + 3*(15 to 19 aged population/5) for 2007-2009 and 4*(10 to 14 aged population/5) + 4*(15 to 19 aged population/5) for 2010-2021. For Lebanon, its entrance age remained at 12-years old and education duration remained six years (then attendees were of age 12-17). Thus, we imputed Lebanon's population by 3*(10 to 14 aged population/5) + 3*(15 to 19 aged population/5). Kosovo does not have any data for entrance age and duration for its secondary education, where we assumed that it starts at 12-years old and lasts for six years (then attendees were of age 12-17). Therefore, we imputed Kosovo's missing population by 3*(10 to 14 aged population/5) + 3*(15 to 10 aged population/5).

Besides, seven countries have available population data for secondary education for at least two years but less than 15 years, for which we visually examined their linearities for available data and used linear interpolation or extrapolation for imputations. Further, we noticed that Honduras has available data from 2007 to 2014 with 2014 seeing a jump in population owing to its change in education duration from five-years to six-years long, and the linear interpolation or extrapolation would overestimate missing values after 2014 if we treated the missing values as lying on a linear trend constructed by 2014 and prior years. Therefore, rather than the linear interpolation or extrapolation, we imputed Honduras's missing (2015-2021) by applying the same year-by-year growth rate of its 10-14 aged population as Honduras's official entrance age for secondary education is at age of 12.

Additionally, one country, Jamaica, has only one year with available data (year 2007), where we imputed Jamaica's missing by applying the same year-by-year growth rate of its 10-14 aged population as Jamaica's official entrance age for secondary education is at the age of 12.

(3) Post-secondary non-tertiary education

We realized that the "post-secondary non-tertiary education" category is not applicable to every country-years, and a country's entrance age and duration variable for this education level would be attached with a "flag" in the UNESCO's database if the country did not provide "post-secondary non-tertiary education" in that year[5,6]. However, there are 16 country-years with available entrance age and duration values while population data missed, and these are the cases that we did imputations for. The 16 country-years include Bosnia and Herzegovina_2013-2018, Brazil_2020, Lebanon_2020, Lebanon_2021, Moldova_2008-2013, Solomon Islands_2020, and Vanuatu_2016. We used linear interpolation or extrapolation to impute these missing values except for Lebanon_2020 and Lebanon_2021 as Lebanon does not have any other available data before year 2021. As Lebanon sets its entrance age at 18-years old and a one-year-long duration, we replaced Lebanon_2020 and Lebanon_2021 with its 15-19 aged population size[7] after divided by five, respectively.

(4) Tertiary education

The UNESCO database does not have entrance age and duration data for tertiary education. Of the 134 LMICs, 37 countries have complete population data between 2007 to 2021 while the 95 countries have data for at least two years, and two countries (Kosovo and Lebanon) have no data for the 15 years. For the 95 countries, we visually examined their linearities for available data and used linear interpolation or extrapolation for imputations. For Kosovo and Lebanon, we assumed that they start tertiary education at the age of 17 and then imputed their missing values by 2*(15 to 19 population/5) + (20 to 24 population).

Reference

1. UNESCO. Official entrance age to primary education. http://data.uis.unesco.org/# (Accessed date: August 28, 2024).
2. UNESCO. Theoretical duration of primary education. http://data.uis.unesco.org/# (Accessed date: August 28, 2024).
3. UNESCO. Official entrance age to lower secondary education. http://data.uis.unesco.org/# (Accessed date: August 28, 2024).
4. UNESCO. Theoretical duration of secondary education. http://data.uis.unesco.org/# (Accessed date: August 28, 2024).
5. UNESCO. Official entrance age to post-secondary non-tertiary education. http://data.uis.unesco.org/# (Accessed date: August 28, 2024).
6. UNESCO. Theoretical duration of post-secondary non-tertiary education. http://data.uis.unesco.org/# (Accessed date: August 28, 2024).
7. UNPD. Total population (both sexes combined) by five-year age group. https://population.un.org/wpp/Download/Standard/Population/ (Accessed date: August 28, 2024).

3. *Imputing country's proportion of government spending on education*

The World Bank database has available proportion of government spending on education for 28 studied countries between 2007 to 2021[1]. For countries with missing values during the study period, we imputed their proportion of government spending on education using different methods based on their data availabilities.

First, there were 10 countries without any data on the proportion of government spending on education between 2007 and 2021. We replaced their missing values with the year-specific average proportions of government spending on education of their country-income groups. Second, two countries have available data for only one year, in which case we replaced their missing values with their available data points. Third, 94 countries have available data points more than two years but less than 15 years. For these countries, we used the year 2015 – the year when the United Nation's Sustainable Development Goals (SDGs) were initially launched – as the cut point and calculated each country's average proportion of government spending on education for the periods of before and since 2015. We then replaced countries' missing values before or since 2015 with the calculated average values in the respective period.

Reference

1. World Bank. Government expenditure on education, total (% of government expenditure). https://data.worldbank.org/indicator/SE.XPD.TOTL.GB.ZS?view=chart (Accessed date: August 28, 2024)

Text S2. Reflexivity Statement

(1) How does this study address local research and policy priorities?

This study is part of a wider multi-country collaboration to harness the power of global data to support young children's learning and development ("Harness Project" hereafter) in low- and middle-income countries (LMICs). The study provides new evidence on the levels and trends of global development assistance supporting early childhood care and education (ECCE) programs in 134 LMICs. The findings can assist the international community in identifying active donors and underfunded recipients, while providing local policymakers with essential data for evidence-based budgeting for their ECCE development. The need for this study arose from wide discussions with local partners and major international organizations that focus on ECCE in LMICs.

(2) How were local researchers involved in study design?

The Harness Project involves researchers from Colombia, China, South Africa, and local ECD networks including the Arab ECD Network, the African ECD Network, and the Asian-Pacific ECD Network. The conceptual framework of this study was presented in project meetings and received feedback from local researchers, implementers, and representatives of international organizations with expertise in coordinating research and policy efforts on ECCE development in LMICs.

(3) How has funding been used to support the local research team(s)?

The project was led by research teams from the University of Oxford in the UK and University of Witwatersrand in South Africa. Regional networks from Africa, and the Arab and Asia-Pacific regions (as mentioned above) received funding for their involvement in research and evidence dissemination.

(4) How are research staff who conducted data collection acknowledged?

This study used secondary data; it did not involve the collection of primary data.

(5) How have members of the research partnership been provided with access to study data?

The data used in the study is publicly accessible and can be assessed by all members.

(6) How were data used to develop analytical skills within the partnership?

The extracted data and early analysis of this study were presented to local and international researchers through several virtual and in-person workshops to enable all research partners to contribute to developing analytic skills in using these data.

(7) How have research partners collaborated in interpreting study data?

The study was presented in workshops or meetings, so all research partners had opportunities at several stages to provide their thoughts about the results and interpretation.

(8) How were research partners supported to develop writing skills?

The study was presented in workshops or meetings, so all research partners had opportunities to learn how the paper was written and how the presentation evolved based on feedback obtained.

(9) How will research products be shared to address local needs?

The aim is to publish the study results in open access journals so that the findings are available to everyone. We also envision presenting these results in relevant workshops and conferences after publication.

(10) How is the leadership, contribution and ownership of this work by LMIC researchers recognized within the authorship?

Depending on the nature of their contributions, the LMIC researchers were included as coauthors.

(11) How have early career researchers across the partnership been included within the authorship team?

We included early career researchers in all the stages of this study. They attended workshops, contributed to study design, data extraction, data analysis, evidence interpretation, and writing.

(12) How has gender balance been addressed within the authorship?

The study was designed and supervised by Dr. Chunling Lu, a female researcher. Anyone who made a significant intellectual contribution to the study was listed as a coauthor.

(13) How has the project contributed to training of LMIC researchers?

The LMIC researchers were involved in regular meetings and received training and mentorship from senior authors. When we conducted workshops or conferences in developing countries, local researchers were invited to participate in the meetings.

(14) How has the project contributed to improvements in local infrastructure?

This project encourages international donors to enhance the share of investments allocated to ECCE programs, including aid toward basic ECCE infrastructure and worker/teacher training for ECCE services. Additionally, this study informs international donors to equip ECCE programs with adequate facilities to improve preparedness against future global crises.

(15) What safeguarding procedures were used to protect local study participants and researchers?

No primary data was collected for this study; therefore, this question is not applicable.
